# Antiaging Strategies and Remedies: A Landscape of Research Progress and Promise

**DOI:** 10.1021/acschemneuro.3c00532

**Published:** 2024-01-12

**Authors:** Rumiana Tenchov, Janet M. Sasso, Xinmei Wang, Qiongqiong Angela Zhou

**Affiliations:** CAS, a Division of the American Chemical Society, 2540 Olentangy River Road, Columbus, Ohio 43202, United States

**Keywords:** Antiaging strategy, parabiosis, senotherapy, hormesis, caloric restriction, physical exercise, diet, antioxidant

## Abstract

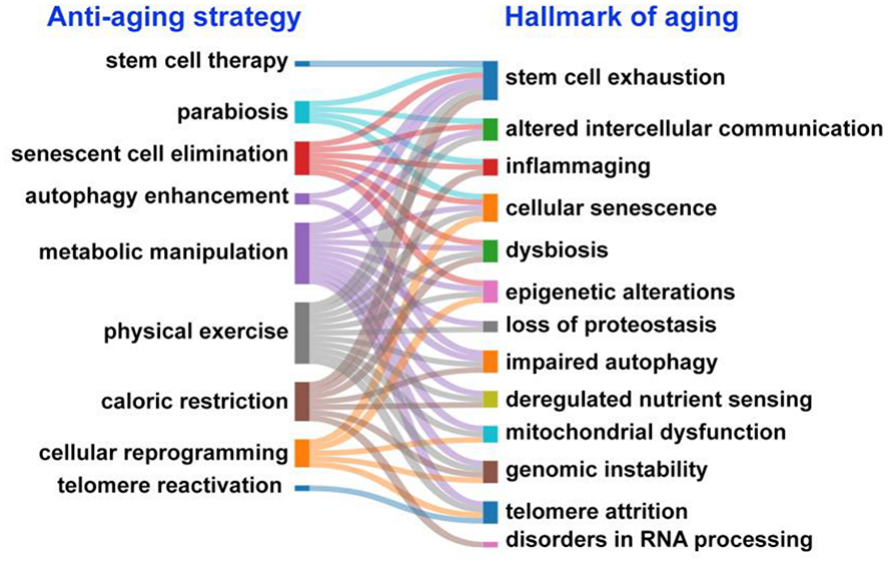

Aging is typified by a gradual loss of physiological fitness and accumulation of cellular damage, leading to deteriorated functions and enhanced vulnerability to diseases. Antiaging research has a long history throughout civilization, with many efforts put forth to understand and prevent the effects of aging. Multiple strategies aiming to promote healthy aging and extend the lifespan have been developed including lifestyle adjustments, medical treatments, and social programs. A multitude of antiaging medicines and remedies have also been explored. Here, we use data from the CAS Content Collection to analyze the publication landscape of recent research related to antiaging strategies and treatments. We review the recent advances and delineate trends in research headway of antiaging knowledge and practice across time, geography, and development pipelines. We further assess the state-of-the-art antiaging approaches and explore their correlations with age-related diseases. The landscape of antiaging drugs has been outlined and explored. Well-recognized and novel, currently evaluated antiaging agents have also been summarized. Finally, we review clinical applications of antiaging products with their development pipelines. The objective of this review is to summarize current knowledge on preventive strategies and treatment remedies in the field of aging, to outline challenges and evaluate growth opportunities, in order to further efforts to solve the problems that remain.

## 1 Introduction

The growing social and economic concern of an aging world population has catapulted aging-related research into the spotlight. Over the past decades, progress in medicine has powered a significant increase in life expectancy worldwide. More than 2 billion individuals are expected to be older than the age of 60 by 2050.^1^ This demographic milepost will come with a major increase in age-related diseases, such as Alzheimer’s disease, cardiovascular disorders, and cancer, which effectively double in incidence every 5 years passing the age of 60.^2^ The relationship between aging and these diseases has triggered fundamental research into the aging mechanisms and approaches to attenuate its effect.

**Figure 1:**
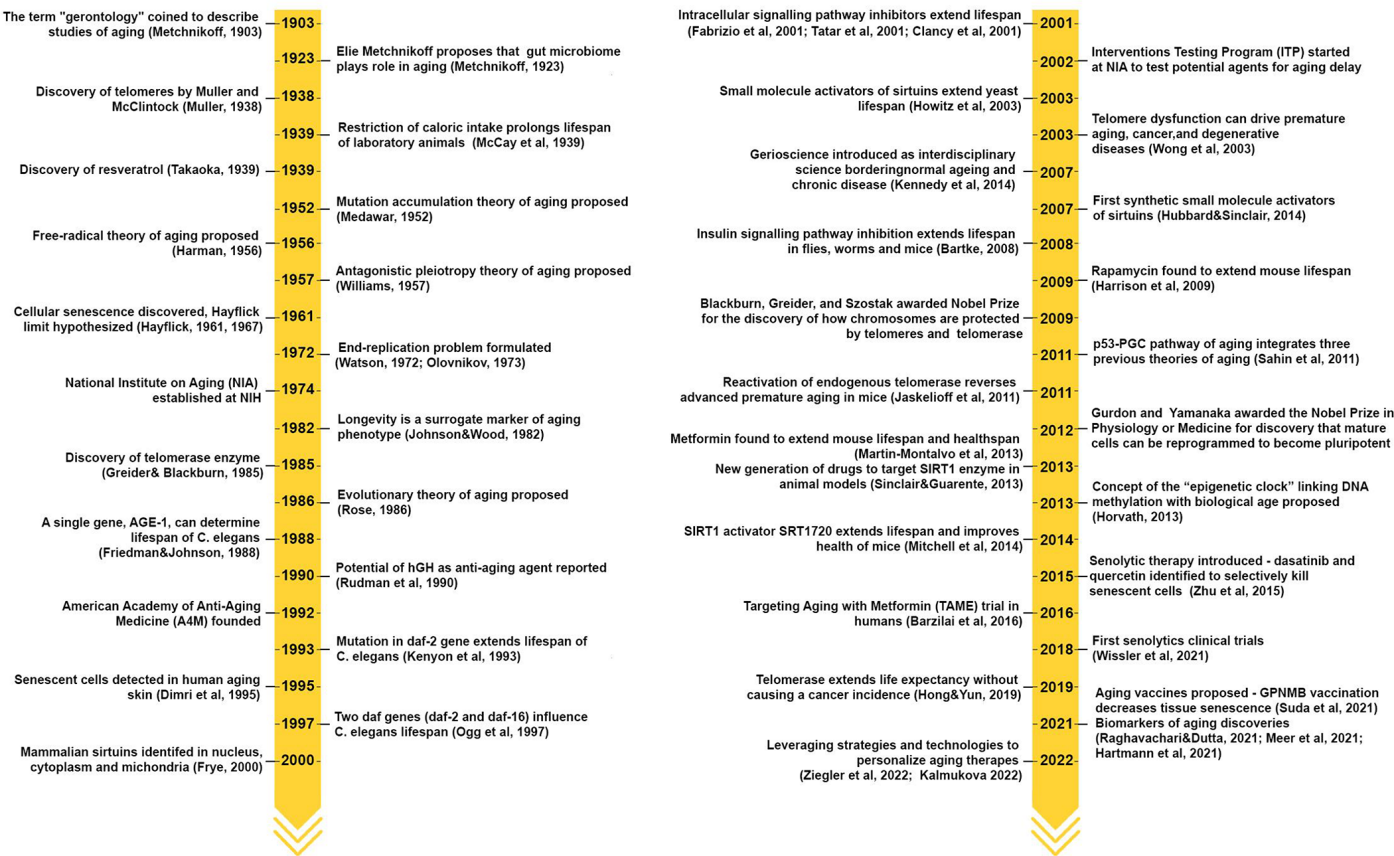
Timeline of key events and discoveries in antiaging research.^7−57^

The efforts to understand and prevent the effects of aging date back centuries, so antiaging research has a long history (Figure 1). In ancient times many cultures developed traditional remedies and practices aimed at promoting longevity and slowing the aging process. For example, ancient Chinese medicine includes herbal remedies and acupuncture techniques designed to promote health and longevity. In the 16th century Italian physician and philosopher Girolamo Cardano wrote a treatise on aging and longevity, in which he discussed the physical and mental changes that occur with age and proposed strategies for promoting health and extending lifespan.^3^ In the 19th century, French physiologist Claude Bernard proposed that aging is caused by changes in the internal ambiance of the body, including changes in metabolism and the accumulation of toxins.^4^ In the early 20th century, scientists began to study the effects of diet and lifestyle on aging, with researchers like Elie Metchnikoff proposing that the gut microbiome plays a role in aging.^5^ Also, Denham Harman proposed the free radical theory of aging, suggesting aging is caused by damage to cells and tissues by unstable molecules called free radicals.^6^

An early step in the field of aging exploration has been the observation that restriction of caloric intake increases lifespan, first demonstrated in mice and rats^9^ and later in other species as well, including primates.^58,59^ Moreover, it was detected that dietary restrictions enhanced not only the lifetime but also the healthspan, suppressing the development of age-related diseases.^60^ A concept emerged that correlation exists between lifespan and healthspan, described as the portion of lifespan free from disease.^61^ A mutation accumulation theory that aging is a result of decline in the power of natural selection after reproduction was proposed in 1952.^11^ Over 30 years later, landmark research in the nematode *Caenorhabditis elegans* demonstrated that the mutant nematode strain exhibits a 40–60% extended lifespan, showing that a single gene, the AGE-1 gene, can control the lifespan of an organism.^22^

The close link between telomeres and aging originates in the discovery of a unique structure at the last portions of the *Zea mays* and *Drosophila melanogaster* chromosomes, hypothesized to play a significant role in preventing chromosome end fusion.^8,62^ In the early 1960s, Hayflick noted that human cells in tissue culture cease dividing after a certain number of cell divisions by a process termed replicative senescence.^13,14^ It was shown later that human fetal cells exhibited finite replication potential of 50–60 doublings, labeled as replicative senescence or the “Hayflick limit”.^13,14^ The link between the ending of cell division and replication of telomeres was revealed in the early 1970s.^15,16^ It was established that telomere attrition takes place in parallel with the replication lifespan of human primary cells, demonstrating that shortened telomeres cause the Hayflick limit.^63^ It took nearly two decades for the suggested causative relationship between replication senescence and telomere shortening to be established.^64,65^ The aging of cells could be associated with alterations in telomere length on genomic DNA.^66,67^ Further on, via animal models, the function of telomeres in aging was authenticated and identified as an essential signaling pathway guiding the aging process.^68^ Telomere dysfunction was shown to accelerate signs and symptoms of aging.^69,70^ Later, transcriptomic studies revealed the p53 aging pathway promoting apoptosis, thus integrating three previously distinct theories of aging: genotoxicity (telomere dysfunction), oxidative damage, and mitochondrial decay.^41,71,72^ Reactivation of endogenous telomerase, an enzyme that could extend telomere sequence, was demonstrated to reverse advanced premature aging in mice.^42^ Of note, adenoviral delivery of telomerase in aged mice was demonstrated to enhance cardiac function after acute myocardial infarction, improve muscle coordination and kidney and liver performance, reduce insulin resistance and subcutaneous fat reduction, increase bone mineral density, and extend life expectancy without triggering a growth in cancer frequency.^51,68^

In the early 1990s, the potential of human growth hormone (hGH) as an antiaging agent acting to increase lean body mass, decrease adipose tissue mass, and increase bone mineral density was reported.^23^ hGH supplements became available, sparking controversy questioning their safety and effectiveness. Later on, aging research focused on the genetic pathways of aging, revealing a complex system of intracellular signaling pathways and higher-order processes.^61,73^

Throughout the 1990s, researchers identified genes linked to aging, including the SIRT1 gene. Substantial attention was given to sirtuins after it was reported that overexpression of the SIR2 gene can prolong yeast lifespan by as much as 70%.^74^ In the 2000s, advances in biotechnology and genetics led to the development of new antiaging therapies, including stem cell therapy, gene therapy, and telomerase activation.^32,33,35,37,38^ In the 2010s and 2020s, the focus of antiaging research shifted toward advanced understanding of the mechanisms of aging and developing interventions that can slow or reverse the aging process, with ongoing studies exploring a range of potential interventions, including senolytics (drugs that clear out senescent cells), metformin (a diabetes drug that may have antiaging properties), and various forms of gene therapy.^41,42,46−57^ Advances in gene editing technology, such as CRISPR/Cas9, allow for more precise manipulation of genes involved in the aging process.^75^ It is important to note that while antiaging research has made significant strides over the years, most of this research is still conducted in non-human systems and there is still much that is not yet understood about the aging process and how to effectively prevent or reverse its effects.

The antiaging research area is still moderate in size, by an order of magnitude less for the number of related publications relative to more advanced areas, including those focused specifically on major age-associated diseases such as cancer, cardiovascular, and Alzheimer’s disease. However, understanding that advanced age is the major risk factor for all of these disorders has brought the rapidly growing field of aging research to the forefront.

**Figure 2:**
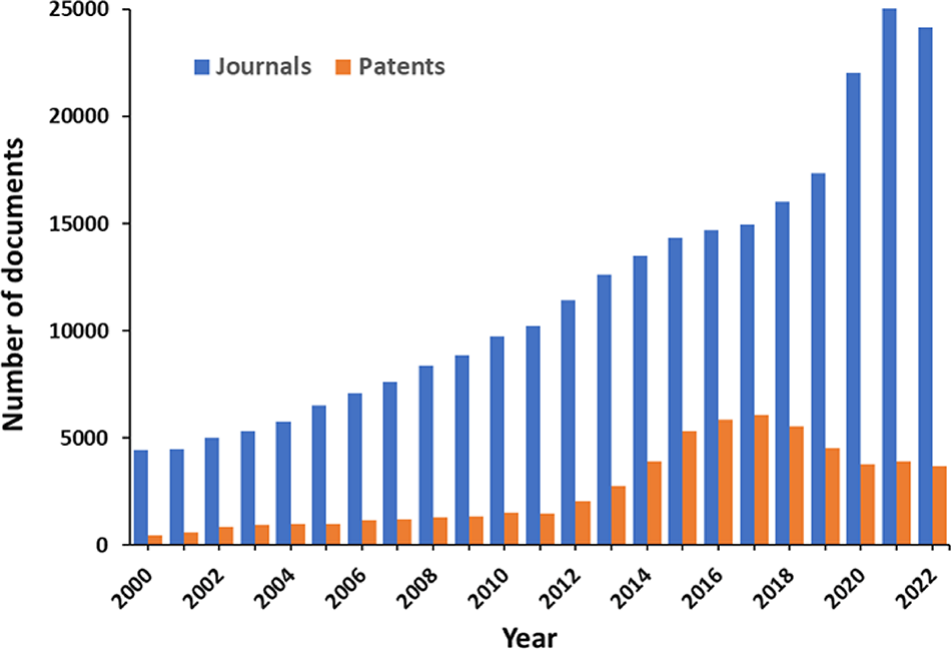
Yearly growth of the number of documents (journal articles and patents) in the CAS Content Collection related to the antiaging strategies and treatments.

The research focused on aging mechanisms and attributes, and the antiaging strategies and medical interventions, has undergone a steady growth, especially intense in the past decade (Figure 2). The desire for enjoying a lifespan in a healthy, youthful condition is a universal human appeal. A characteristic feature of many recent antiaging strategies is that they are focused on prevention, trying to avoid disease via health preservation rather than cure disease after it occurs. Its basic approach is replacement therapy.^76^ Since appropriate knowledge and expertise allowing to slow or halt the homeostatic decline in basic informational, regulatory, and protective molecules upon aging has not yet been established, replacement of those products such as hormones, cofactors, antioxidants, and others is routinely employed. Such a proactive approach meant to slow down or escape the development of age-associated disease is more rational than a reactive, symptomatic approach.^76^

In this paper, we review the advances in the research on antiaging strategies and perspectives. We examine data from the CAS Content Collection,^77^ the largest human-compiled collection of published scientific information, and analyze the publication landscape of recent research in this area in an effort to provide insights into the research advances and developments. We review the current concepts related to the major antiaging approaches to combat aging on the molecular, cellular, and organismic level. We further assess the state-of-the-art antiaging strategies and explore their correlations with age-related diseases, based on the data from the CAS Content Collection. Well-known and currently examined antiaging agents have been specified. Finally, we inspect clinical applications of antiaging products with their development pipelines.

The objective of this review is to provide a broad overview of the evolving landscape of current knowledge on preventive strategies and treatments in the field of aging, to outline challenges and evaluate growth opportunities, in order to further efforts to solve the problems that remain. The novelty and merit of the article stem from the extensive, wide-ranging coverage of the most up-to-date scientific information accumulated in the CAS Content Collection allowing unique, unmatched breadth of landscape analysis and in-depth insights.

## 2 Antiaging Strategies

Aging is typified by a gradual loss of physiological fitness, leading to deteriorated functions and enhanced vulnerability. Numerous age-related factors and attributes have been identified as hallmarks of aging, and the potential mechanisms of aging are extensively examined. At the molecular level, aging attributes include DNA damage, epigenetic modifications, telomere shortening, protein aggregation, and accumulation of aberrant mitochondria and lysosomes.^78−80^ At the cellular and organismic level, aging hallmarks comprise cellular senescence, stem cell exhaustion, impaired nutrient sensing, and chronic low-level inflammation.^78−80^

The modern concept of antiaging strategies is focused on promoting healthy aging and extending healthy lifespan. Rather than trying to reverse the aging process, antiaging strategies aim to reduce the risk of age-related diseases and disabilities, maintain physical and cognitive function, and enhance overall well-being later in life.^81,82^ Key features of the modern concept of antiaging strategies include the following:

- A focus on prevention: Antiaging strategies are centered on prevention, with an emphasis on reducing the risk of age-related diseases and disabilities before they occur. This may involve lifestyle changes such as healthy eating, exercise, and stress management, as well as preventive medical interventions such as vaccines and screening tests.
- An integrated approach: Antiaging strategies recognize that aging is a multidimensional process that affects many aspects of our lives. This may involve interventions that target multiple aspects of aging, such as physical activity to maintain muscle mass and cognitive training to maintain mental function.
- A personalized approach: Antiaging strategies are based on the understanding that there is no “one size fits all” approach to promoting healthy aging and that interventions may need to be tailored to individual needs and circumstances. This may involve the use of personalized medicine, genomics, and other technologies to identify individuals at higher risk of age-related diseases and tailor interventions accordingly.
- An emphasis on quality of life: Antiaging strategies are focused on promoting not just longevity but also quality of life in later years. This may involve interventions that promote physical and cognitive function, as well as social and emotional well-being.
- A multidisciplinary approach: Antiaging strategies involve a wide range of disciplines, including medicine, biology, psychology, sociology, and public health. Researchers and practitioners from these different fields work together to identify effective interventions and promote healthy aging at the individual, community, and societal levels.

**Figure 3:**
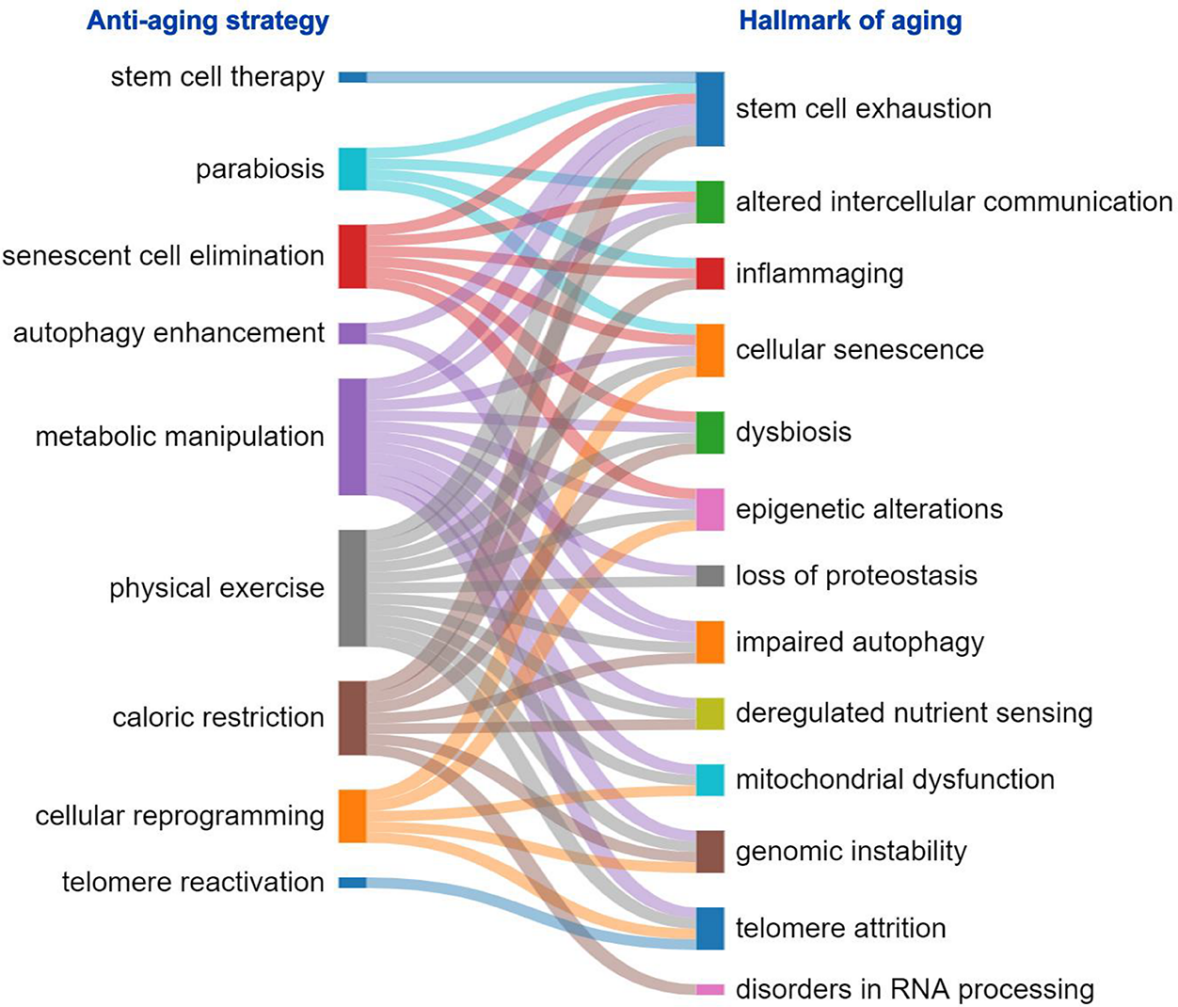
Relationship between antiaging strategies and the hallmarks of aging they counteract.

A variety of antiaging strategies targeting the hallmarks of aging have been explored largely, including parabiosis (blood exchange), metabolic manipulation, senescent cell elimination, and cellular reprogramming. Most of them are targeted to multiple aging hallmarks (Figure 3).

### 2.1 Parabiosis (Blood Exchange)

Blood transfusion/exchange has been believed to exhibit rejuvenation effect since ancient times. Parabiosis is a procedure of placing young blood into an old animal (heterochronic parabiosis) by joining the circulatory systems of the two animals so that they share their blood circulations.^83,84^ The procedure has been reported to bring about an antiaging effect by specifically activating molecular signaling pathways in liver, muscle, or neural stem cells of the old parabiont resulting in increased tissue regeneration.^85,86^ In the search for the physiological background of such rejuvenating effects, certain soluble blood factors have been identified as in part responsible, including the chemokine CCL11, the growth differentiation factor 11 (GDF11), a member of the TGF-β superfamily, and oxytocin.^86−89^ It has been implied that parabiosis reverses age-related decline by targeting several aging hallmarks including stem cell exhaustion, cellular senescence, altered intercellular communication, and inflammaging (Figure 3).^84^ Still, the mechanisms of action of the factors identified as responsible for the rejuvenating effects in parabiosis animal studies remain largely unclear, with many unresolved issues. Further, a study of the lifespans of old and young rat pairs demonstrated that older partners lived for 4–5 months longer than controls, indicating that circulation of young blood might affect longevity.^90^ A start-up company, Alkahest (San Carlos, CA, USA), initiated a clinical trial assessing the safety, tolerability, and feasibility of infusion of plasma from young donors to treat patients with mild-to-moderate Alzheimer’s disease.^91^ The trials implied that the treatment with young fresh frozen plasma has been safe, well tolerated, and feasible.^92^ Certain limitations of the study were the small sample size, short duration, and variation in study design, but the conclusion was that the results demand further examination in larger, double-blinded placebo-controlled clinical trials.

### 2.2 Metabolic Manipulation (mTOR Inhibitors)

The mammalian target of rapamycin (mTOR) signaling pathway has been identified as significant participant of cellular metabolism relating nutrient sensing to critical cellular processes that energize cell growth and proliferation.^93^ mTOR belongs to a family of phosphatidylinositol kinase-related kinases, which are known to mediate cellular responses to stress factors such as DNA impairment and nutrient deficiency. In model studies on organisms such as *Saccharomyces cerevisiae*, *Caenorhabditis elegans*, and *Drosophila melanogaster*, mTOR has been identified as a negative regulator of life span.^93^ The mTOR is involved in various distinctive aging pathways, such as nutrient sensing deregulation, proteostasis loss, autophagy impairment, mitochondrial dysfunction, cellular senescence, and stem cell function decline.^94,95^ It has been reported to also manipulate gut microbiota and its metabolites.^96^

One of the foremost pharmacological actions prolonging life span in certain model organisms is rapamycin.^93^ Rapamycin is a natural product isolated from *Streptomyces hygroscopicus*, which exhibits antifungal, immunosuppressant, and antitumor proprieties, mediated by the inhibition of its target, mTOR.^97,98^

Consistent with its central role in cellular metabolism, in synchronizing protein synthesis, energy metabolism, and autophagy, mTOR has been identified as an attractive target to ameliorate age-related pathologies. Inhibition of the mTOR pathway has profound effects on longevity and age-associated phenotypes across a wide variety of organisms.^93^ However, the inherent mechanisms are still largely unclear and extensive research is still needed to fill many deficiencies in the available knowledge related to the mTOR function in the context of aging.

### 2.3 Senotherapy

Senotherapy involves development of potential therapeutic agents and approaches to explicitly target cellular senescence, a cell condition associated with aging and age-associated pathologies.^99^ There are multiple senotherapeutic strategies including geroprotection which refers to strategies preventing or reversing cell senescence by preventing DNA damage, oxidative stress, proteotoxicity, telomere shortening, and other senescence promoters.^100^ Senescence-associated secretory phenotype (SASP) inhibition is another strategy that is achieved with using agents restricting proinflammatory SASP production^101,102^ such as glucocorticoids,^103^ statins (simvastatin),^104^ JAK1/2 inhibitors (ruxolitinib),^105,106^ NF-κB and p38 inhibitors, and IL-1α blockers. Senescent cell elimination is another strategy that utilizes small molecule senolytic agents to transiently disable their survival pathways and antiapoptotic systems.^48,107,108^ Unlike SASP inhibitors, senolytics can be successful following intermittent rather than continuous administration.^109^ On the other hand, senescent cell modulation uses a wide range of agents called senomorphics suppressing senescent phenotypes without killing cells.^110^ Gene therapy is another approach that edits cells’ genes to enhance their resistance to aging and senile diseases and prolong the lifespan of an organism.^111,112^ Lastly, the Klotho gene has been identified as a gene involved with aging,^113^ so senotherapeutic strategies have been developed to modulate Klotho expression, including administration of exogenous Klotho, Klotho agonists as well as indirect approaches, via regulation of the foodome and gut microbiota.^114^

### 2.4 Cellular Reprogramming

Cellular reprogramming includes the conversion of terminally differentiated mature cells into induced pluripotent stem cells (IPSCs). This process involves complete dedifferentiation; i.e., the somatic cell identity has been erased as cells are converted to a pluripotent state. Reprogramming-induced rejuvenation strategy termed epigenetic rejuvenation implicates using a cocktail of four factors known as Yamanaka factors, a finding for which a Nobel prize was awarded in 2012.^43^ It was shown that overexpression of four transcription factors (Oct3/4, Sox2, Klf4, and c-Myc, referred to as “OSKM” factors or the “Yamanaka factors”) reorganizes the epigenetic landscape and converts somatic cells to a IPSCs.^115,116^ The process of rejuvenation by cellular reprogramming results in the amelioration of certain aging hallmarks such as mitochondrial dysfunction, telomere attrition, changes in epigenetic alterations, genomic instability, and senescence.^84^

Furthermore, it has been recognized that cellular identity is determined by epigenetic changes rather than by genomic DNA alterations.^117^ The process of generating iPSCs has been improved with time and has also been accomplished via chemical induction rather than gene expression.^118−120^ IPSCs have effectively unlimited regenerative ability and offer the potential for tissue replacement to counteract age-induced decline. Therefore, iPSCs induction offers the potential of directed, personalized regenerative therapy for currently incurable diseases, such as certain neurodegenerative diseases, heart infarction, diabetes, and others.^121^ Partial cellular reprogramming in mice has demonstrated promising results in alleviating age-associated symptoms without increasing the risk of cancer.^122^

### 2.5 Telomere Reactivation

Telomere shortening takes place during aging and is related to certain age-associated diseases, including osteoarthritis, atherosclerosis, coronary heart disease, and atrial fibrillation.^123−125^ It has been reported that aging can be impeded by telomerase overexpression; however, that can stimulate tumorigenesis.^126−128^ Recently, that adverse effect has been eliminated by developing antiaging strategies based on a telomerase activation, telomerase expression activation, and telomerase gene therapy. It has been found that an extract of the plant *Astragalus membranaceus*, TA-65, is a telomerase activator, which can restore telomere length without provoking cancer and enhance age-related markers, including glucose tolerance, bone health, and skin quality.^129^ Further, TERT transcription activator and sex hormones have been reported to be directly engaged in telomerase activation, which avoided telomere shortening and enhanced lifespan.^130,131^ Reactivation of telomerase expression by using a gene therapy strategy has been reported as a successful approach for aging delay and lifespan extension of mice without cancer incidence.^132^

### 2.6 Hormesis

Hormesis is defined as a dose-responsive phenomenon typified by low-dose stimulation and high-dose inhibition.^133^ It has been identified as an overcompensation response for mild environmental stress related to the body’s intrinsic ability of self-maintenance and repair. In fact, the beneficial effects of mild stress on aging and longevity have been examined for a long time.^134^ The term “hormetins” has been introduced to denote conditions and compounds that can induce health-beneficial physiological hormesis.^135^

Beneficial hormetic effects of repeated mild heat stress on aging human cell cultures in terms of their structural and functional integrity have been demonstrated.^136^ Other mild stresses have also been reported to defer aging and stimulate longevity in experimental animals. These include mild hyperthermia, irradiation, hypergravity, exercise, and caloric restriction, as well as chemical agents such as heavy metals, pro-oxidants, acetaldehyde, alcohols, and resveratrol.^137−140^ In experimental animals, mild dietary stress without malnutrition postpones the majority of age-related physiological changes, prevents aging disease, and extends lifespan.^133^

An area of interest regarding the dose–response model of hormesis in the brain is newly developing.^141,142^ Cellular models of neurodegenerative diseases have implied that neurohormesis impacts cognitive performance as well as oxidative stress-modulated neurodegenerative responses.^141,142^ The brain cells exhibit survival response networks that are regulated by genes involved in preserving cellular homeostasis during stressful conditions (vitagenes).^143^ These genes recognize stressful incidents and work toward cell survival under stress conditions.^144^

### 2.7 Hormonal Replacement

Hormone levels decline with age, as a result of the reduced gland secretion.^145^ Diminished hormone levels are related to decline in bone mineral density, muscle mass, sexual appeal, erectile activity, and intellectual potential.^146−149^ Therefore, hormone supplementation has been widely applied to help improve the quality of life in the elderly.^150^

Perimenopause women suffer from discomforting symptoms such as hot flashes or vaginal dryness, so hormone replacement has been applied to lessen or eliminate them. Estrogens and progesterone have been beneficial in osteoporosis treatment.^151^ During the recent decades, compounded bioidentical hormone therapy (cBHT) has become popular.^152^ The term bioidentical refers to the chemical structure of menopausal hormone therapy being identical to that of endogenous hormones. Therapies include estriol alone and in combination with estradiol (biest) and estrone (triest), estradiol or estrone alone, progesterone, and androgens.^153^ However, evidence of safety issues has progressively accumulated, such as irregularity of cBHT content, potential increase in endometrial cancer risk, lack of bioavailability data, and incomplete adverse event reporting.^153−157^

Elderly men typically exhibit low testosterone levels which have been associated with certain age-related pathologies.^158^ Sarcopenia and osteoporosis are more common in older men. Low plasma testosterone levels have been associated with sarcopenia and osteoporosis, as well as mild cognitive impairment and Alzheimer’s disease.^159,160^ Therefore, testosterone replacement therapy has been found advantageous as it can augment muscle mass, physical strength, and body-mass index in elderly men.^157,161^ However, adverse effects of the testosterone therapy such as polycythemia and risk of aggravating prostate cancer have been reported as well.^150,162^ Selective androgen receptor modulators (SARMs) are nonsteroidal compounds with favorable oral bioavailability that were developed in the early 2000s in an attempt to overcome the pharmacological limitations of steroidal androgen receptor agonists (i.e., testosterone and DHT), which have known associations with liver and heart disease.^163^ Recent systematic review on their safety indicates that their use may be associated with drug-induced liver injury, rhabdomyolysis, tendon rupture, and adverse cardiovascular outcomes.^164^

Dehydroepiandrosterone (DHEA) is a precursor for sex hormones and is transformed into androgen or estrogen, respectively. The decrease of plasma DHEA levels in the elderly has been associated with certain age-related pathologies. In some reports, DHEA supplementation has been reported beneficial for alleviating vasomotor symptoms, preserving the integrity of the immune system, reducing bone loss, and increasing muscle mass and strength, enhancing physical performance, improving body mass index, as well as for mood and sexual function, however there is generally little evidence to support antiaging claims.^165−171^ The adverse effects of DHEA are minimal.^172,173^

### 2.8 Prebiotic/Probiotics and Fecal Transplantation

Microbiota plays such an essential role in human physiology and pathology that it is considered as an essential organ.^174−176^ Recent reports on the involvement of microbiota in regulating health status and lifespan^177,178^ have triggered significant growth in the field of life sciences research and industry.^179^ Numerous studies have provided proof that microbiota-targeted interventions can have a therapeutic power not only for age-related diseases but also for delaying aging and promoting longevity. Longevity has been correlated to *Firmicutes* bacteria rearrangement and *Proteobacteria* enrichment and also a substantial decrease in *Faecalibacterium prausnitzii* and elevation of *Eubacterium limosum* levels within the gut microbiota.^180^

Microbiota composition can be strongly modulated by certain factors including diet, probiotics/prebiotics/synbiotics, physical activity, drugs, and psychological stress. An imbalance in the microbiota called dysbiosis is a state characterized by distinct alterations in the microbiome resulting in modifications in their functional composition and metabolic performance. One microbiota-aimed intervention for dysbiosis is probiotic supplementation containing mainly *Bifidobacterium* and *Lactobacillus* species.^181,182^ Another microbiota-aimed intervention is fecal microbiota transplantation which is a procedure used to restore the intestinal ecosystem through transferring of feces filtrate from a healthy donor into the gastrointestinal tract of the recipient.^183^ Recently, its potential benefit and safety in pathologies commonly associated with aging, type 2 diabetes mellitus, metabolic syndrome, atherosclerosis, and neurodegenerative diseases have been demonstrated.^184,185^

### 2.9 Caloric Restriction/Intermittent Fasting

One of the first antiaging approaches was developed from the observation that caloric intake control can increase lifespan, first demonstrated in mice and rats.^9^ The reduction of caloric intake by some 10–30% as compared to *ad libitum* food intake has been demonstrated to extend the longevity of various species. Furthermore, among all antiaging interventions, dietary interventions have demonstrated the greatest promise. Recently, two related dietary interventions, caloric restriction and intermittent fasting, have been reported to effectively prolong the healthy lifetime of the nervous system by affecting basic metabolic and cellular signaling pathways that regulate longevity.^186^ Multiple interactive pathways and molecular mechanisms exist by which caloric restriction and intermittent fasting benefit neurons. Indications exist that the advantages of caloric restriction are associated with modification of the metabolic rate. The key physiological routes that have been inferred as potential mechanisms by which caloric restriction stimulates longevity include (i) activation of AMP protein kinase,^187^ (ii) sirtuins activation,^188,189^ (iii) insulin-like growth factor-1 signaling inhibition,^190^ and (iv) mammalian target of rapamycin inhibition.^191,192^ These pathways promote the protein chaperones, neurotrophic factors, and antioxidant enzymes production, which facilitate cells managing stress and fight disease.

Although calorie restriction has exhibited the greatest efficiency of all antiaging interventions, it is a difficult strategy to successfully apply in humans, since it requires a high level of determination and self-control. As an alternate route, compounds that emulate the outcome of caloric restriction on health and longevity without an actual restriction named “calorie restriction mimetics” have been discovered (reviewed further in the paper).^193^

### 2.10 Physical Exercise

Physical exercise has been well validated as an effective antiaging intervention. Regular physical activity of the elderly plays a vital role at a multisystem level, avoiding muscle atrophy, mending or sustaining cardiorespiratory health and cognitive performance, and enhancing metabolic activity.^194−196^ Recommendations predicated on the most recent American College of Sports Medicine Guidelines advise that physical exercises for elderly need to involve aerobic exercise, muscle strengthening, and endurance training, as well as flexibility and neuromotor exercises.^197^ The prospective benefits of the recommended physical activities include the following:

- enhancing neurogenesis and reducing neurodegeneration and cognitive decline;
- reducing blood pressure and augmenting various cardiovascular activities, e.g., maximum cardiac output, blood flow, endothelial performance, vagal tone and heart rate adaptability, and heart preconditioning;
- advancing respiratory activity by enhancing ventilation and gas exchange;
- augmenting metabolic activity in enhancing the resting metabolic rate, protein synthesis in muscles, and lipid oxidation;
- boosting muscle performance and body composition by enhancing muscle strength and stamina, maintaining or restoring balance, motor control, and joint flexibility and mobility, as well as decreasing weight and local adiposity, and enhancing muscle mass and bone density.^195,196^

Furthermore, physical exercise exhibits a significant antiaging impact at a cellular level, related to each and every aging hallmark.^194^ Exercise plays a role in maintaining genomic stability. Data analysis comprising hundreds of genetic elements from a large number of exercising elderly individuals found a reduction in DNA methylation percentage in a dominating number of genes, specifically in genes associated with a cancer-defeating miRNA gene network.^198^ Exercise exhibits a beneficial impact on telomere length as well, antagonizing the regular age-provoked telomere attrition. Possible mechanisms have been debated correlating exercise and telomere length declines to alterations in telomerase activity, inflammation, oxidative stress, and reduced skeletal muscle satellite cell content.^199^ It has been documented that acute exercise protocols are correlated with increased heat-shock proteins transcription, which indicates potential positive impact of physical activity on proteostasis.^200^

### 2.11 Stem Cell Therapy

Stem cell therapies are widely used in the regenerative medicine due to their intrinsic biological characteristics, including plasticity, self-renewal, and multiway differentiation ability. Stem cell treatment includes human autograft or allograft cultured stem cells locally injected into specific parts of the body or administered by intravenous infusion.^201,202^ Bringing active stem cells into the body can rejuvenate existing cells and allow the body to age more gently and even reverse some impacts of aging. Currently, neural stem cells, bone marrow mesenchymal stem cells, umbilical cord mesenchymal stem cells, adipose stem cells, embryonic stem cells, and human induced pluripotent stem cells are the most closely related antiaging agents and exhibit direct (cellular replacement) and indirect (paracrine) effects.^202^

Pluripotent stem cells naturally differentiate when culture conditions no longer support their pluripotency.^203^ Hence, pluripotent stem cells can be directed toward preferred cell identities when specific stimuli are supplemented, such as those available during embryonic differentiation. There are many examples of pluripotent stem cells differentiation. The differentiation of pluripotent stem cells into renal podocytes, hematopoietic progenitor cells, neurons, endothelial cells, cardiac muscle cells, retinal progenitor cells, pancreatic β islet cells, or ciliated epithelial cells infers no limits to human tissue modeling *in vitro*.^204−211^ The reported progress in organoids development also demonstrates the advanced knowledge in cell manipulations. Three-dimensional cultures of pluripotent cells let them organize and differentiate into spheroid structures, with several cohabitant cell types. The most sophisticated current organoid models relate to the brain, intestine, kidney, heart, or retina.^212−221^ With the emergence of cell 3D-printing technologies using pluripotent stem cells or differentiated cells as inks, impressive progress has been successful in the formation of heterogeneous tissues. One such development, ear cartilage regeneration, utilizes this technology.^222−224^

Stem cells may be capable of deferring aging in several ways:

- Tissue regeneration: Stem cells can differentiate into various cell types and can thus replace damaged tissue, potentially reversing the impacts of aging.
- Augmenting body’s repair mechanisms: Stem cells stimulate production of growth factors and other signaling molecules, thus enhancing the repair mechanisms of the body, allowing it to maintain healthy tissue and postpone age-related alterations.
- Immune modulation: Stem cells may act as immunomodulators, affording a way to maintain a healthful immune system and avoid age-related immune dysfunction.
- Lowering inflammation: Stem cells may have anti-inflammatory properties, thus reducing the chronic aging-related inflammation.
- Oxidation protection: Stem cells may safeguard against oxidative stress, a process that can result in cell damage throughout aging.
- Mitochondrial support: Stem cells can preserve mitochondrial health by intercellular communication via tunneling nanotubes physically transferring mitochondria from stem cells to unhealthy aging cells.

### 2.12 Dietary Supplementation

Dietary supplements, such as vitamins, minerals, amino acids, essential fatty acids, flavonoids, plants and herbs, and accessory food ingredients, are considered valuable and safe materials for prevention and treatment of chronic and acute diseases.^225^

Interest in the therapeutic capacity of vitamin supplements, specifically vitamin D, for stimulating human longevity and reducing risk of aging-related pathologies is currently a hot topic in clinical studies.^226^ It has been reported that vitamin D and its metabolites can delay age-associated diseases by lowering oxidative stress, strengthening innate immunity, preventing DNA damage and supporting DNA repair pathways, controlling mitochondrial and glucose metabolism, defeating cellular senescence, and enhancing telomerase activity.^225,227−229^ These research findings imply that vitamin D consumption exhibits antiaging properties. A recent systematic review has also reported that vitamin D intake notably lowers the risk of acute respiratory infections.^230^ Other vitamins such as B vitamins, K vitamins, and others have been examined as dietary supplements that assist healthy aging.^225^ Vitamin B12 in combinations with bacopa, lycopene, and astaxanthin successfully alleviated cognitive delay associated with brain aging.^231^ Improving vitamin interventions by combining them with other health-supporting supplements might result in successful clinical utilization.

Minerals are vital for health and well-being and are typically obtained from diet. Deficiencies in some minerals have been associated with age-related diseases such as dysregulated calcium levels being correlated to accelerated cellular aging. Calcium-related changes upon aging are also regarded as a contributing factor for neurological degenerative disorders such as Parkinson’s disease. There is evidence that aging individuals are at risk of calcium deficit, so appropriate calcium dietary intake can lower the risk of osteoporosis and bone fractures.^232,233^ Zinc is another mineral that promotes healthy aging and supports the healthspan. Zinc has numerous biological roles, such as antioxidant, anti-inflammatory, immune modulation, DNA damage prevention, and others. Zinc insufficiency is often reported upon aging and contributes to age-associated disorders. Zinc supplementation is considered an efficient choice for controlling age-related health pathologies, including neurological disorders, infectious diseases, age-related macular degeneration.^234,235^

Long-chain polyunsaturated fatty acid supplementation is believed to protect human health by affecting biological activities, notably aging processes.^236,237^ Recent reports suggest that higher levels of ω3 fatty acids in circulation correlate with lower risk of premature death from age-associated diseases such as cardiovascular disease and cancer.^238^ Dietary ω3 fatty acid consumption has been related to cognitive improvements in aging populations.^239^ The ω3 fatty acids have been shown to exhibit anti-inflammatory, antiapoptotic, antioxidant, and endothelial vasodilator effects.^240^

### 2.13 Autophagy Enhancement

Autophagy is a natural degradation process for removing unnecessary or dysfunctional cellular components via a lysosome-dependent mechanism.^81,241,242^ Dysfunctional autophagy is known to contribute to neurodegeneration,^243^ while improvement of autophagy has been believed to be useful for treating a variety of disorders, including metabolic disorders, neurodegenerative and infectious diseases, and cancers.^241^ An unharmed autophagy performance in neurons exhibits a neuroprotective effect since autophagy expedites the elimination of pathogenic kinds of proteins such as α-synuclein involved in Parkinson’s disease^244^ or tau-protein in Alzheimer’s disease.^245,246^ Therapies involving autophagy enhancers such as rapamycin and lithium have been reported to engender favorable effects in animal models of Parkinson’s disease, such as reduced α-synuclein aggregation, oxidative stress, mitochondrial dysfunction, and neurodegeneration.^244^ Delaying aging and stimulating longevity achieved through food deprivation and caloric restriction are facilitated through upregulation of autophagy because reducing autophagy diminishes the antiaging outcomes of caloric restriction.^241,247,248^ Furthermore, studies have indicated that enhancing autophagy could reinstate the regenerative capacity of aging stem cells.^249,250^ Therefore, autophagy enhancing interventions would likely facilitate successful aging and increased longevity.

### 2.14 Brain Antiaging Strategies

The brain is extremely sensitive to the effects of aging, manifested mainly as changes in cognitive capacity, as well as enhanced risk for developing certain neurological disorders.^251,252^ Taking care of brain health requires the maintenance of brain functions in several aspects including cognitive ability, motor function, emotional health, as well as tactile function.^253^

It has been evidenced recently that one of the best strategies for healthy brain aging is regular aerobic exercise.^254^ It is suggested that exercise likely remains the most effective intervention for healthy brain aging because it stimulates strategic energy-sensing pathways that modulate multiple hallmarks of aging:

- Dysregulated energy metabolism is a major hallmark of brain aging. Upon aging, fasting glucose levels increase, which is correlated with accelerated brain aging and inferior cognitive function.^255,256^ Exercise is a kind of hormesis, an energetic stress (reduced cellular energy levels) to which the brain reacts with multiple metabolic, mitochondrial, and cellular responses. Essential examples include upregulation of glucose transporters (GLUT) and enhanced signaling through the low-energy sensors AMP protein kinase, sirtuin 1, and the mammalian target of rapamycin (mTOR) pathway.^195,256^
- Mitochondrial dysfunction is another universal hallmark of aging,^79^ and it is specifically enhanced with brain aging.^256^ It has been reported however that hippocampal gene expression patterns in older adults who are physically active exhibit enhanced mitochondrial energy production.^257^
- Curiously, exercise has been related to oxidative stress since enhanced metabolism upon exercise increases ROS generation. However, this effect is likely hormetic because exercise-associated oxidative stress results in greater long-term antioxidant ability.^195^
- Animal studies have demonstrated that exercise induces neurogenesis in the hippocampal dentate gyrus and is associated with increased neurogenesis, superior neuronal structure, and improved memory.^258,259^
- Telomere maintenance, which promotes the neuronal stem cells production, may protect against neurodegenerative diseases. It has been reported that regular exercise is associated with longer telomeres and reduced cellular senescence in both mice and humans.^79,195,256^

## 3 Landscape View of the Antiaging Strategies Research: Insights the from CAS Content Collection

The CAS Content Collection^77^ is the largest human-compiled collection of published scientific information, which represents a valuable resource to access and keep up to date on the scientific literature all over the world across disciplines including chemistry, biomedical sciences, engineering, materials science, agricultural science, and many more, allowing quantitative analysis of global research publications against various parameters including time, geography, scientific area, medical application, disease, and chemical composition. Currently, there are over 500 000 scientific publications (mainly journal articles and patents) in the CAS Content Collection related to aging physiology and antiaging strategies. There is a steady growth of these documents over time, especially intense in the past decade (Figure 2).

**Figure 4:**
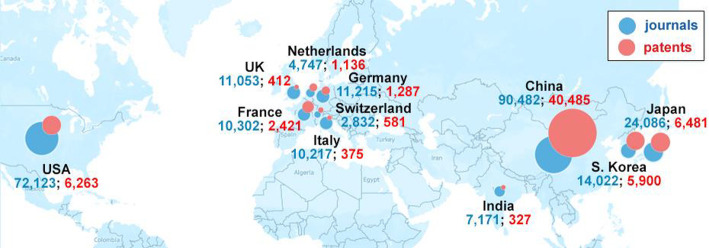
Top countries with respect to the number of journal articles and patents related to antiaging strategies and treatments.

Authors from China, the United States, Japan, and South Korea have published the highest number of journal articles and patents related to antiaging strategies (Figure 4).

**Figure 5:**
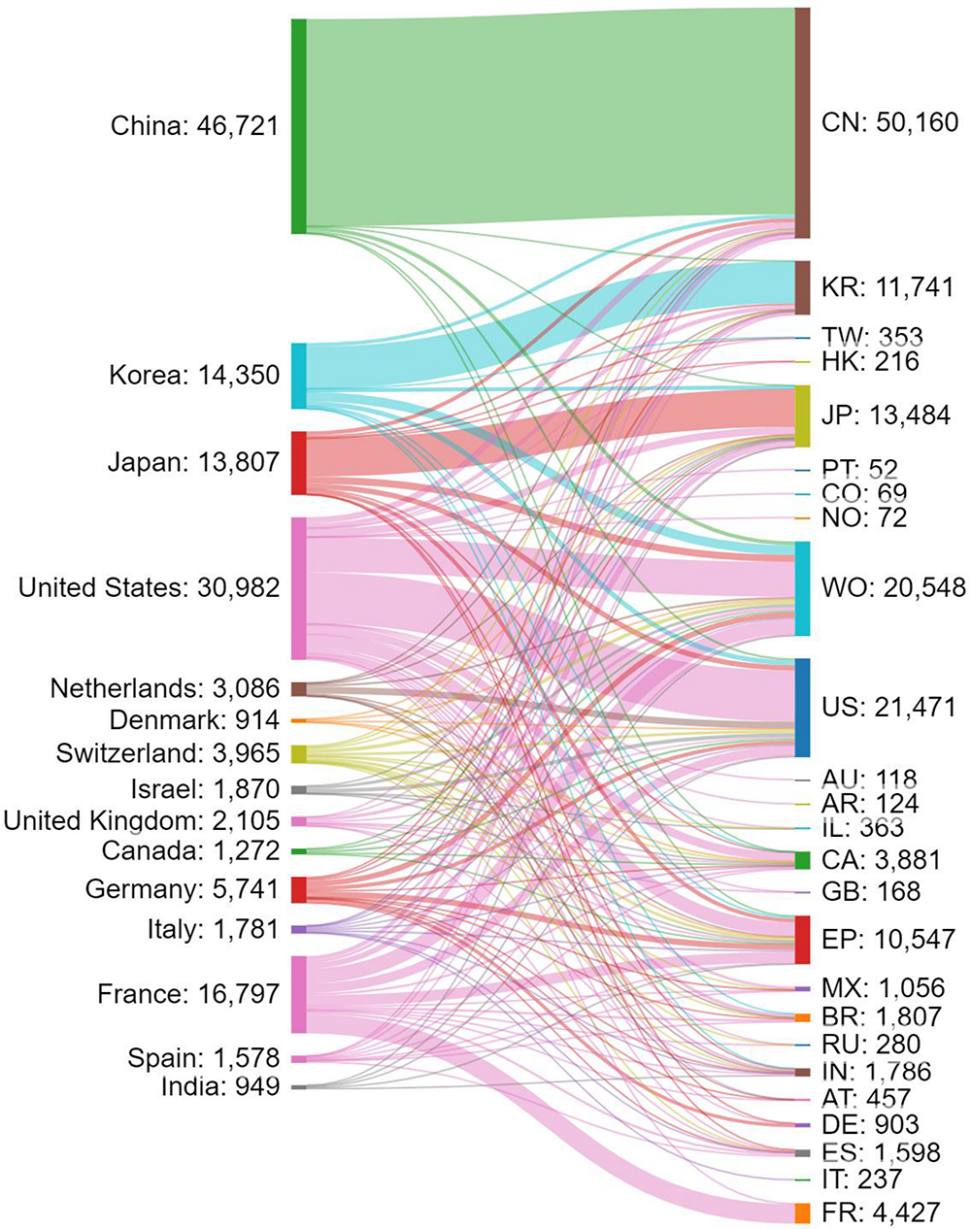
Flow of patent filings related to aging mechanisms and antiaging strategies from different patent assignee locations (left) to various patent offices of filing (right). The abbreviations on the right indicate the patent offices of China (CN), Korea (KR), Israel (IL), Hong Kong (HK), Argentina (AR), Australia (AU), Russian Federation (RU), United States (US), World Intellectual Property Organization (WO), Taiwan (TW), Mexico (MX), Japan (JP), Canada (CA), Brazil (BR), Austria (AT), European Patent Office (EP), India (IN), Great Britain (GB), Spain (ES), France (FR), Germany (DE), and Italy (IT).

Patent protection is territorial, and therefore the same invention can be filed for patent protection in several jurisdictions. We thus searched for all related filings on aging mechanisms and antiaging strategies. Certain patent families might be counted multiple times when they filed in multiple patent offices. Figure 5 presents the flow of patent filings from various applicant locations to a variety of patent offices. There are various patent filing strategies: some patent assignees, such as those from China, for example, file exclusively in their home country patent office (CN), with only a small portion filing through the World International Patent Office WIPO (WO) or other jurisdictions. Others, for instance, the United States and France-based applicants, have a comparable numbers of their home country and WO filings and a sizable number of filings at other patent offices such as the European Patent Office (EP).

**Figure 6:**
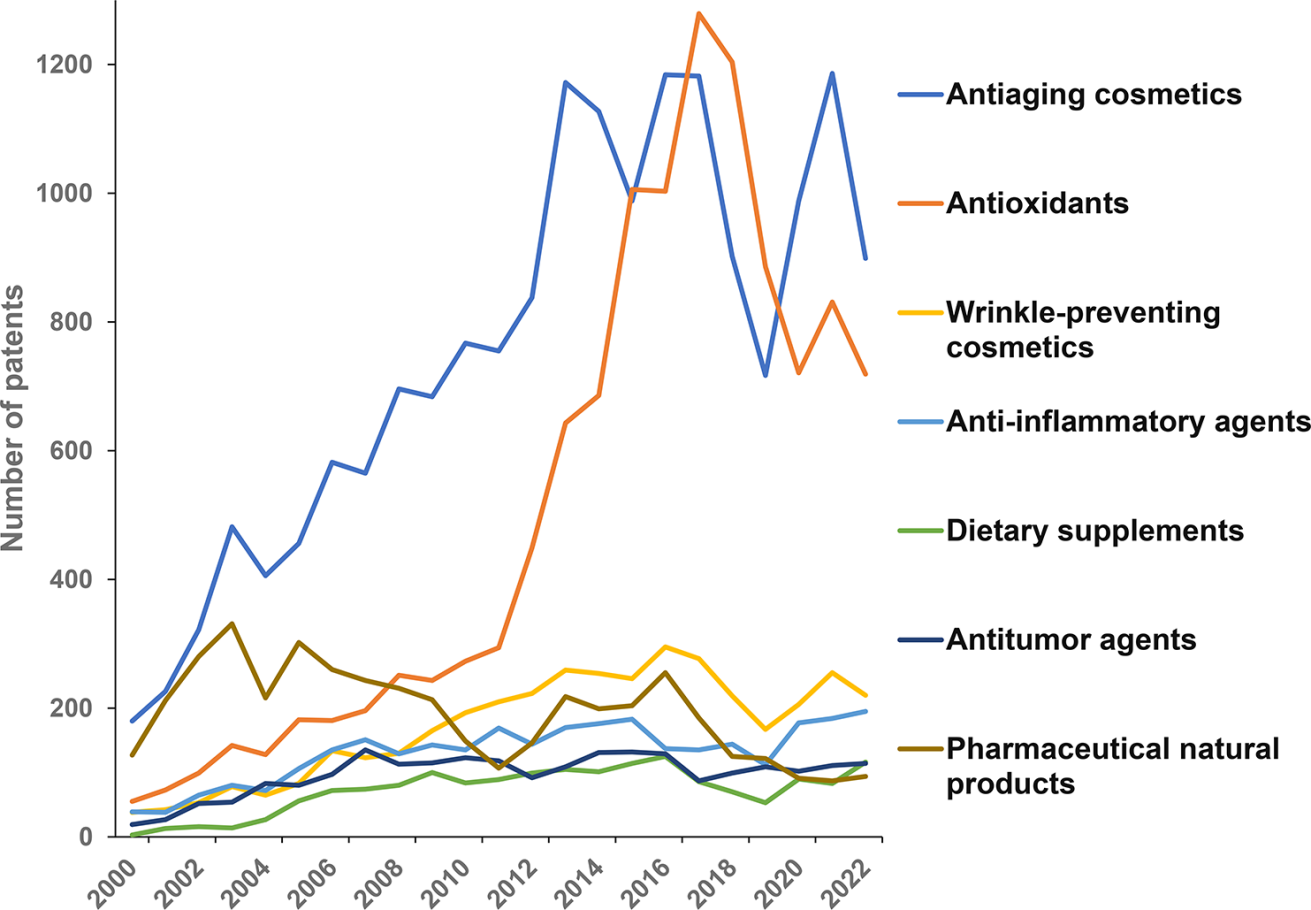
Popular concepts in patents related to aging mechanisms and antiaging strategies according to the CAS Content Collection.

Figure 6 presents the most popular antiaging related concepts in the overall patent landscape of the CAS Content Collection. The concepts, antiaging cosmetics, and antioxidants are the distinct leaders.

**Figure 7:**
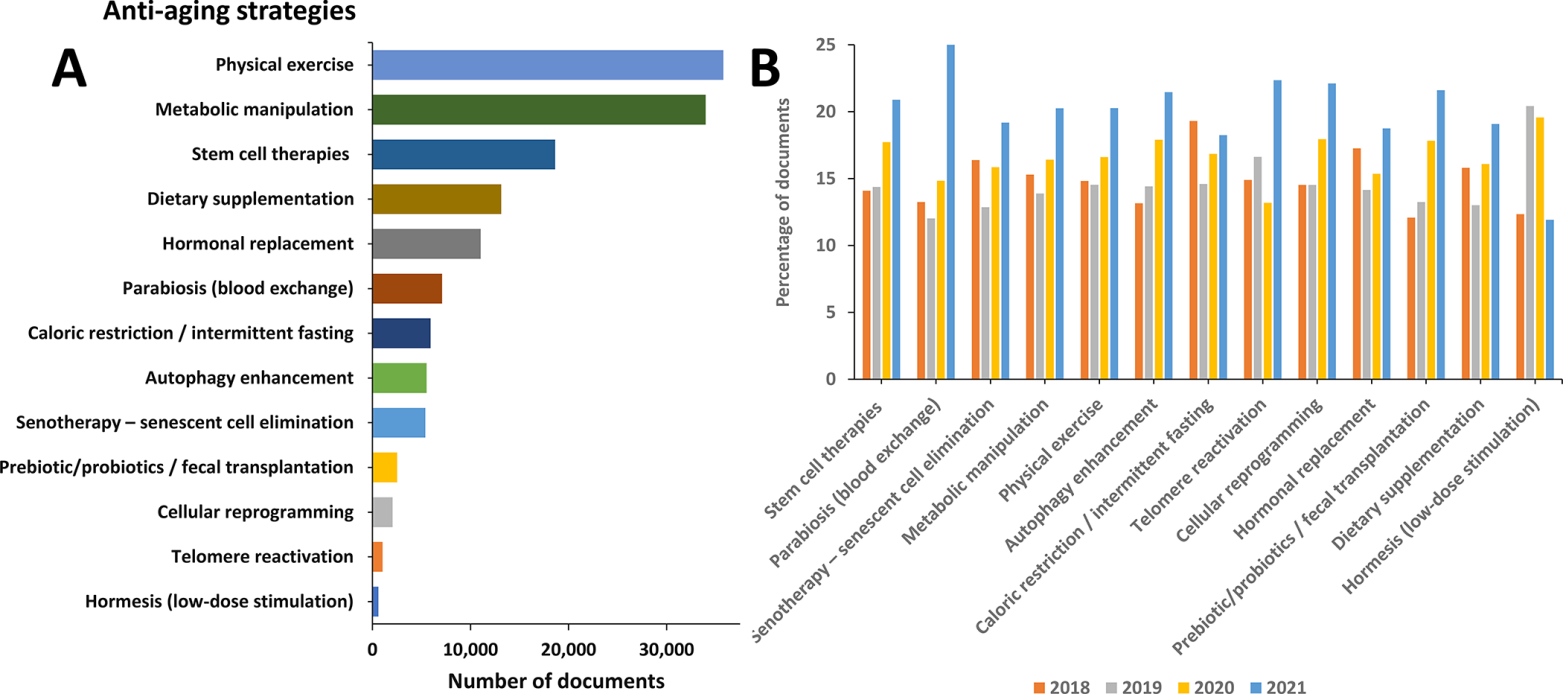
Antiaging strategies explored in the scientific publications: (A) number of publications exploring antiaging strategies; (B) trends in number of publications exploring antiaging strategies during the years 2018–2021.

We examined also the distribution and trends in the published documents dealing with prominent antiaging strategies (Figure 7). Physical exercise comes up as the most studied approach, along with the metabolic manipulation (Figure 7A). This can be well anticipated considering the fact that these strategies are the ones related with the highest number of aging attributes (Figure 3). Physical exercise has been well justified as one of the particularly effective and highly recommended antiaging practice. Regular physical activity of the elderly has a proven vital role at a multisystem level, avoiding muscle atrophy, mending or maintaining cardiorespiratory health and cognitive performance, and boosting metabolic activity. Next, the inhibition of the mTOR pathway has been shown to exhibit profound effects on longevity and age-related phenotypes across a wide variety of organisms.^93^

With respect to the annual trends in the antiaging strategies related publications, parabiosis (blood exchange) attracts substantial recent attention (Figure 7B). It has been suggested that blood exchange reverses the age-associated decline by targeting multiple attributes of aging including stem cell exhaustion, cellular senescence, altered intercellular communication, and chronic inflammation (Figure 3). A steady growth in the number of recent publications has been documented with respect to the prebiotic/probiotics and fecal transplantation as well (Figure 7B). Multiple studies have conveyed evidence that microbiota-targeted interventions can have a strong therapeutic power not only for age-associated diseases but also for delaying aging and stimulating longevity. Longevity has been correlated to certain bacteria phyla alterations: *Firmicutes* rearrangement and *Proteobacteria* enrichment, decrease in *Faecalibacterium prausnitzii*, and elevation of *Eubacterium limosum*.^180^ Furthermore, microbiota composition can be intensely modulated by activities and interventions including diet, probiotics/prebiotics/synbiotics, physical activity, drugs, and psychological stress.^181,182^ Fecal transplantation is another recently emerging intervention being looked at for a wide range of conditions including pathologies commonly associated with aging such as diabetes, metabolic syndrome, atherosclerosis, and neurodegenerative diseases.^184,185^ Remarkably, recent animal studies showed that, similar to blood exchange, transfer of young donor microbiota into older animals can reverse age-related central nervous system and retinal inflammations and cytokine signaling, effects which are coincident with increased intestinal barrier permeability.^260^ Microbial modulation emerges as therapeutically beneficial in preventing inflammation-associated tissue degradation in later life.^260^

**Figure 8:**
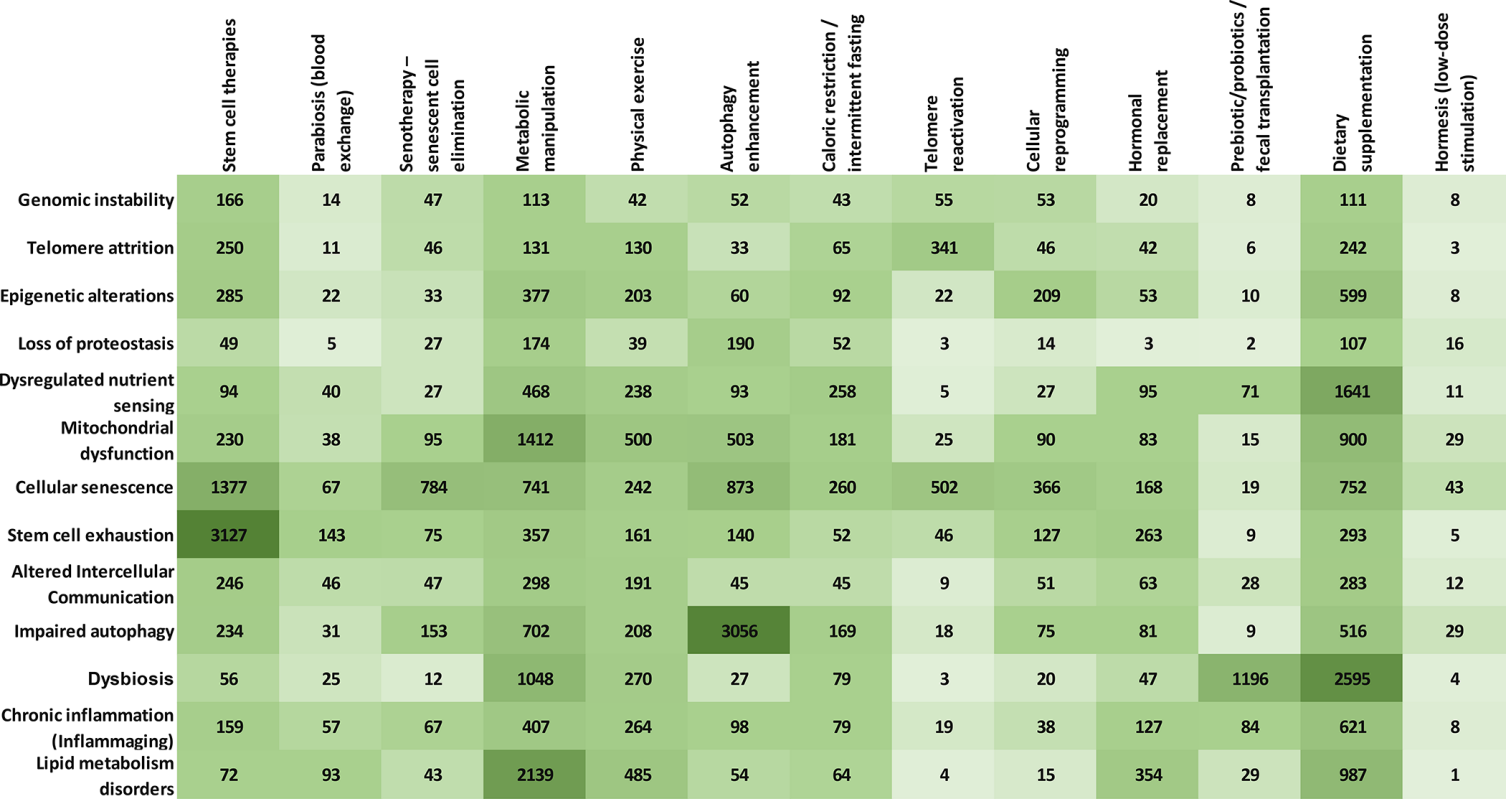
Correlation of the number of documents related to the hallmarks of aging with the antiaging strategies.

Next, we explored the correlations between the hallmarks of aging and the antiaging strategies as reflected by the number of documents in the CAS Content Collection (Figure 8). Along with some foreseeable strong correlations such as stem cell exhaustion–stem cell therapies, lipid metabolic disorders–metabolic manipulations, impaired autophagy–autophagy enhancement, cellular senescence–senotherapy, and dysbiosis–prebiotic/probiotic/fecal transplantation, there are some that are less anticipated and instructive:

- Metabolic manipulations appear closely aligned with the mitochondrial dysfunction and dysbiosis.

Metabolic manipulation have been recognized as a noteworthy strategy in mitochondrial medicine.^261^ Cellular alterations triggered by mitochondrial dysfunction include enhanced reactive oxygen species production, enhanced lipid peroxidation, and modified cellular calcium homeostasis. Therefore, metabolic manipulation is aimed to prevent oxidative damage by ROS, adjustment of lipid peroxidation, amendment of altered membrane potential, and restoration of calcium homeostasis.^261^

- Autophagy enhancement associates with cellular senescence.

Autophagy has been initially reported to inhibit mesenchymal stem cells senescence by eliminating damaged cytoplasmic organelles and macromolecules, yet recent studies found that autophagy can in fact promote mesenchymal stem cells senescence by triggering the production of senescence-associated secretory proteins (SASP).^262^

- Epigenetic alterations and mitochondrial dysfunction are well linked to dietary supplementation.

Nutritional epigenetics is a novel subfield of epigenetics dealing with the specific effects of bioactive food constituents on epigenetics and their relations to phenotypes. Elucidating the epigenetic features prompted by bioactive food components might set the stage for personalized nutritional therapeutics and enhance the understanding of how organisms respond to specific diets or nutrients.^263^ A recent study reported that fruit and juice epigenetic effects as assessed by DNA methylation are related to independent immunoregulatory routes, indicating the distinct health benefits of fruits and juices. The discovery of such differences among foods is the first step toward personalized nutrition.^264^ Further, adequate nutrient levels are important for mitochondrial function as certain particular micronutrients play essential roles in energy metabolism and ATP production.^265^

- Stem cell therapies strongly affect cellular senescence.

One of the advantages of mesenchymal stem cell-based therapies is that they have demonstrated effectiveness by targeting multiple pathological pathways.^266^ Recently, a study has indicated that mesenchymal stem cells could alleviate renal cellular senescence.^267^

- Lipid metabolism disorders appear impacted by dietary supplementation.

Recently, there is remarkable interest in the health benefits of food constituents against chronic diseases in which elevated lipids are a major issue.^268^ The nutritional regulation of lipid metabolism has become an essential tool to prevent or reverse the development of lipid metabolism disorders.

- Dietary supplementation emerges as well correlated with virtually all aging attributes and especially with dysbiosis.

**Figure 9:**
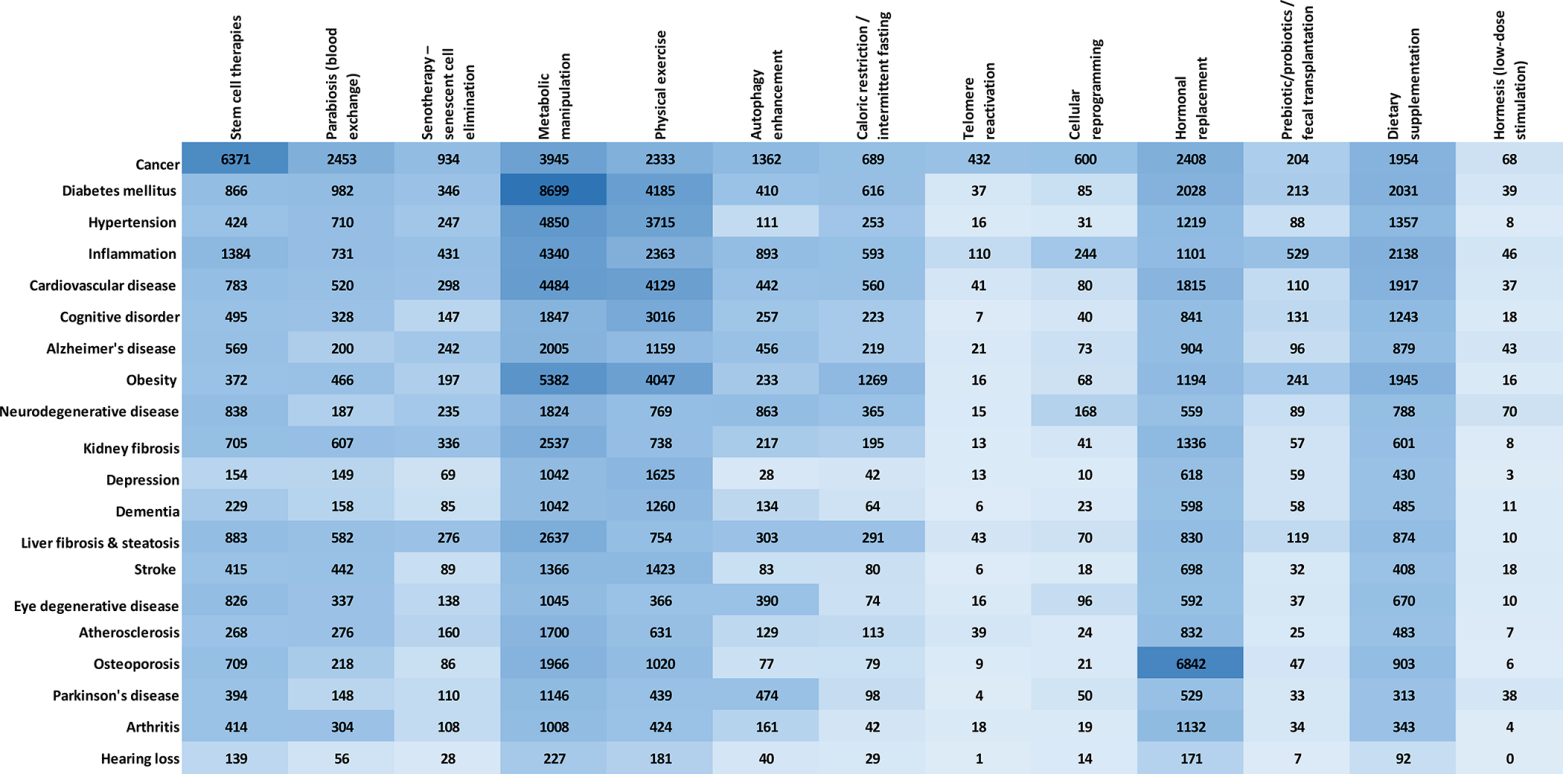
Correlation of the number of documents related to the age-related diseases with the antiaging strategies.

We explored the correlations between the age-related diseases and the antiaging strategies as reflected in the number of documents in the CAS Content Collection (Figure 9). Generally, metabolic manipulations, physical exercise, hormonal replacement, dietary supplementation, and stem cell therapies appear as well researched approaches against multiple pathologies.

Some particular correlations are noteworthy:

- Stem cell therapies exhibit strong correlation with cancer.

Stem cell transplants are procedures that restore the hematopoietic stem cells in patients receiving high doses of chemotherapy or radiotherapy used to treat certain cancers.^269−271^ Stem cell transplants do not typically work against cancer directly but rather help recover the body’s ability to produce stem cells after such high-dose interventions. Still, in multiple myeloma and some leukemias, a stem cell transplant may work against cancer directly, due to the graft-versus-tumor effect taking place after allogeneic transplants, when leukocytes from the donor attack the remaining cancer cells after high-dose treatments. This effect enhances the success of the treatments.^269^ Growing evidence is also indicating that cancer stem cells can differentiate into various cell types, including noncancerous cells. Scientists are taking advantage of this observation through a treatment called differentiation therapy.^272^

- Metabolic manipulation approach is well correlated with virtually all age-associated diseases but specifically with diabetes and obesity.

Indeed, mTOR dysregulation is known to result in a number of metabolic pathologies, including obesity and type 2 diabetes.^273^

- Physical exercise seems like another potent approach against multitude of age-related diseases but especially against diabetes, cardiovascular disease, and obesity.

There is undeniable evidence of the efficacy of regular physical activity in the prevention of multiple chronic diseases, including age-associated disorders, e.g., cardiovascular disease, diabetes, cancer, hypertension, obesity, depression, and osteoporosis.^274^ Physical exercise can enhance the cognitive power, prevent cognitive impairment, and attenuate the development and progression of Alzheimer’s disease.^275,276^ Physical activity interventions successfully reduce the incidence of type 2 diabetes.^277^

- Dietary supplementation is also a prominent approach against multiple age-related diseases, especially diabetes, inflammation, cancer, cardiovascular disease, obesity, hypertension, and cognitive disorders.
- Hormonal replacement exhibits strong correlation with osteoporosis

Estrogen deficiency is known as the major factor in the pathogenesis of postmenopausal osteoporosis.^278^ Hormone replacement therapy, either estrogen alone or a combination of estrogen and progestin, has been approved for the prophylactics and therapy of osteoporosis in women and has been reported to rapidly normalize turnover, preserving bone mineral density at all skeletal sites.^279,280^

## 4 Antiaging Drugs

The search for remedies that can prevent, slow, or reverse aging has a long history (Figure 1) and is currently attracting a lot of attention. There is a steady growth of the number of journal articles related to the antiaging strategies and treatments over time, rather explosive in the past three years (Figure 2). The number of patents rapidly grew until 2016–2017, possibly correlating with the initial accumulation of knowledge and its transfer into patentable applications. Later on, the patent number is at a steady level, after a peak in 2017, perhaps awaiting the forthcoming breakthroughs in the antiaging drug awareness.

A new domain of geriatric medicine termed geroscience has recently emerged aiming to develop new tools to enhance healthspan.^281^ The key claim of geroscience is that aging can be controlled to delay or prevent the beginning of aging-related disorders by targeting the global aging process rather than treating aging disorders once they occur. Applying geroscience-based strategies in healthcare and clinical practice could open an opportunity to increase the proportion of healthy individuals and reduce morbidity to a restricted period near the end of life, which would bring significant economic and social benefits.

The extreme complexity of interactions among factors and pathways is implicated in the process of aging, and looking for pharmacological strategies targeting aging is certainly a very difficult task. Still, substantial progress has been made in this research field, recently. Significant antiaging capacity has been revealed in some natural compounds as well as in certain classes of chemically synthesized substances. These include calorie restriction mimetics such as metformin, rapamycin, and resveratrol.^282^ Using several model organisms, such as yeast, worms, flies, and rodents, these potential drugs have been reported to extend life expectancy by up to 25–30%.^283^ Great expectations are currently related to the development of senolytics, drugs that can selectively eliminate senescent cells.^284^ Another hopeful class of pharmacological agents involve substances targeting the epigenetic control of gene expression such as histone deacetylase inhibitors, including sodium butyrate, suberoylanilide hydroxamic acid, and trichostatin A.^285^

Another strategy of searching for antiaging agents involves assessing the healthspan-promoting ability of drugs that have been already approved by the regulatory authorities to treat certain chronic pathological conditions. These include common medications such as aspirin, metformin, melatonin, certain statins, vitamins, and antioxidants.^286^ A benefit of repurposing such drugs is that their long-term safety has been repeatedly examined and approved, and their possible side effects have been well-known through various clinical trials and examinations. Evidence has been accumulated that such common drugs may indeed enhance health and well-being in elderly individuals suffering from a variety of age-related chronic pathologies.^281,286^

The collection of currently explored antiaging agents is dominated by natural compounds, either pure substances or extracts. Vital nutrients such as certain vitamins, minerals (as micronutrients), amino acids, polyunsaturated fatty acids, probiotics, and plant metabolites, such as polyphenols and terpenoids, are recognized for their ability to prevent aging and promote healthy aging. Natural antiaging sources are present in a wide variety of foods including meat, fish, poultry, fruits, vegetables, herbs, cereals, nuts, grains, legumes, dairy products, cocoa/chocolate, as well as in beverages such as juice, tea, coffee, and wine. Natural extracts from plants and herbs such as green tea, turmeric, yerba mate, thyme, licorice, mulberry, and grape are also known for their antiaging, mainly antioxidant potencies.^287−291^

### 4.1 Calorie Restriction Mimetics

Drug candidates and dietary supplements categorized as caloric restriction mimetics delay aging and extend healthspan and lifespan by modifying aging-associated pathways similarly to caloric restriction and intermittent fasting strategies. The term has been coined in a pivotal 1998 paper.^292^ Because caloric restriction requires continuous effort and firm discipline, identifying active agents that produce similar but effortless effects have attracted significant attention. Such compounds would open the possibility of enhancing physiological functions, expanding longevity, and lowering risk of chronic diseases.^282^ Various organic compounds have been shown to modulate antiaging pathways in a manner similar to caloric restriction and intermittent fasting. Examples of the most widely examined caloric restriction mimetics include rapamycin, metformin, resveratrol, acarbose, aspirin, glucosamine, nicotinamide riboside, and spermidine.^193,282,293^

**Rapamycin** (CAS RN 53123-88-9) represents one of the best-known caloric restriction mimetics.^282^ It is a macrolide compound isolated from *Streptomyces* bacteria. Rapamycin is an mTOR inhibitor and historically used to avoid immunosuppressive organ transplant rejection.^294^ Inhibition of mTOR is known to activate autophagy, a cellular process recognized as a powerful antiaging approach, believed to also mediate the rapamycin effect.^295^ Rapamycin treatment has been reported to extend lifespan and to enhance health markers in model organisms.^294^ Certain reported adverse side effects such as cataract risks, infections, as well as insulin resistance restrain the use of rapamycin to delay aging and stimulate active searches for analogous mTOR inhibitors, so-called rapalogs, in an effort to find potential drugs with a better safety profile.^296^ One of the first-generation rapalogs, everolimus (CAS RN 159351-69-6), found within the 95% similarity limit of rapamycin by CAS SciFinder^n^ ^297^ has been approved to prevent organ rejection and for cancer treatment. Other compounds found within the 95% similarity limit of rapamycin by CAS SciFinder^n^^297^ are listed in Table 1. Later generation rapalog compounds are currently being examined in preclinical and clinical studies.^296^

**Table 1:** Top Compounds Found within the 95% Similarity Limit of Rapamycin by Using CAS SciFinder^n^

substance	CAS RN
7-*epi*-Rapamycin	157182-37-1
32-Desmethoxyrapamycin	83482-58-0
7-Demethoxyrapamycin	157054-88-1
40-*O*-(3-Hydroxy)propylrapamycin	159351-72-1
Rapamycin, 42-*O*-(2-methoxyethyl)-	169288-19-1
32-Desmethylrapamycin	141392-23-6
(27*R*)-27-Deoxo-27-ethoxyrapamycin	2250063-46-6
(27*R*)-27-Deoxo-27-methoxyrapamycin	2250062-73-6
SAR 943	186752-78-3
(31S)-Rapamycin	253431-35-5
Rapamycin, 27-deoxo-27-hydroxy-, (27*R*)-	221895-97-2
31-*O*-Methylrapamycin	159351-88-9
Novolimus	151519-50-5

**Metformin** (dimethylbiguanide hydrochloride, CAS RN 657-24-9), an antidiabetic drug widely used for treatment of type 2 diabetes, is also considered a caloric restriction mimetic providing antiaging benefits, believed to be mediated by the activation of AMPK in *C. elegans* and rats.^293,298^ It has been demonstrated that metformin administration prolongs the lifespan in animal models, including mammals.^44^ In humans, metformin is shown to be beneficial against certain age-associated diseases, such as cancer, metabolic syndrome, as well as cognitive deficits and cardiovascular disorders.^299−301^ However, metformin may cause adverse side effects, such as vitamin B deficiency and cognitive decline in older adults, as well as lowered testosterone levels, possibly resulting into erectile dysfunction.^302,303^

**Resveratrol** (3,5,40-trihydroxystilbene, CAS RN 501-36-0), a SIRT activator, which is a natural polyphenolic phytoalexin mostly abundant in red wine and grape skins but also in berries and peanuts, has been widely investigated.^304^ Resveratrol has been reported to prolong lifespan and delay the onset of aging-related markers in model organisms.^305,306^ It also demonstrates ability to protect against an assortment of age-related disorders including type 2 diabetes, Alzheimer’s disease, and cancer.^307^ A widespread opinion is that antiaging and longevity effects of resveratrol are mediated by sirtuins activation.^308^ Resveratrol has been shown to target certain stress-related cellular mechanisms, such as AMPK and SIRT1, with both targets, AMPK and SIRT1, required for resveratrol-induced health promotion. Stimulation of SIRT1 brings about protein deacetylation and autophagy induction.^309−313^ Other similar small-molecule SIRT1 activators have been examined including **SRT-1720** (CAS RN 925434-55-5) and **SRT2104** (CAS RN 1093403-33-8) and have been demonstrated to prolong lifespan, reducing inflammation and protecting from neurodegeneration in model organisms.^36^

**Spermidine** (CAS RN 124-20-9) is a polyamine known to induce autophagy in various model organisms, which is considered causal for some of the reported beneficial effects such as cardioprotection.^314^ The autophagy induction is believed to be result of the inhibition of certain acetyltransferase activity by spermidine.^315^ Furthermore, spermidine is able to promote mitophagy.^316^

**Aspirin** (acetylsalicylic acid, CAS RN 50-78-2), a nonsteroidal anti-inflammatory drug, has been in widespread medical use for a long time. It is known to quickly metabolize into salicylate *in vivo*, which inhibits EP300 by competing with acetyl-CoA, thus activating autophagy and exhibiting lifespan-prolonging effect in model organisms.^317^

**Acarbose** (CAS RN 56180-94-0) is a widely used antidiabetic drug known for its ability to decrease plasma glucose and cholesterol levels by inhibiting intestinal α-glucosidase and pancreatic α-amylase.^318^ It was recently reported to exhibit promise as an antiaging drug by increasing lifespan in mice^319,320^ and relieve certain age-related pathologies.^321^ Acarbose has been hypothesized to extend life span by controlling gut microbiota, thus reducing inflammatory reactions and therefore diminishing the risk of mitochondrial disorders and telomere attrition.^322^

### 4.2 Senolytic Drugs

The small-molecule drugs known as senolytics selectively eliminate senescent cells. Senescent cells accumulate upon aging and cause multiple age-associated disorders. Furthermore, senescent cells are known to develop a senescence-associated secretory phenotype (SASP) to provoke immune clearance, which boosts chronic inflammation and plays a key role in aging and age-related diseases.^284,323^ It is believed that chronic inflammation activated by senescent cells is among the main grounds of aging-associated pathologies.^283^ Senolytic drugs are intended to delay, prevent, relieve, or treat such age-related diseases. As expected for therapeutics targeting one of the very fundamental aging mechanisms, the prospective uses of senolytics are variable, hopefully alleviating multiple conditions, opening a new route for curing age-related dysfunction and diseases.

**Figure 10:**
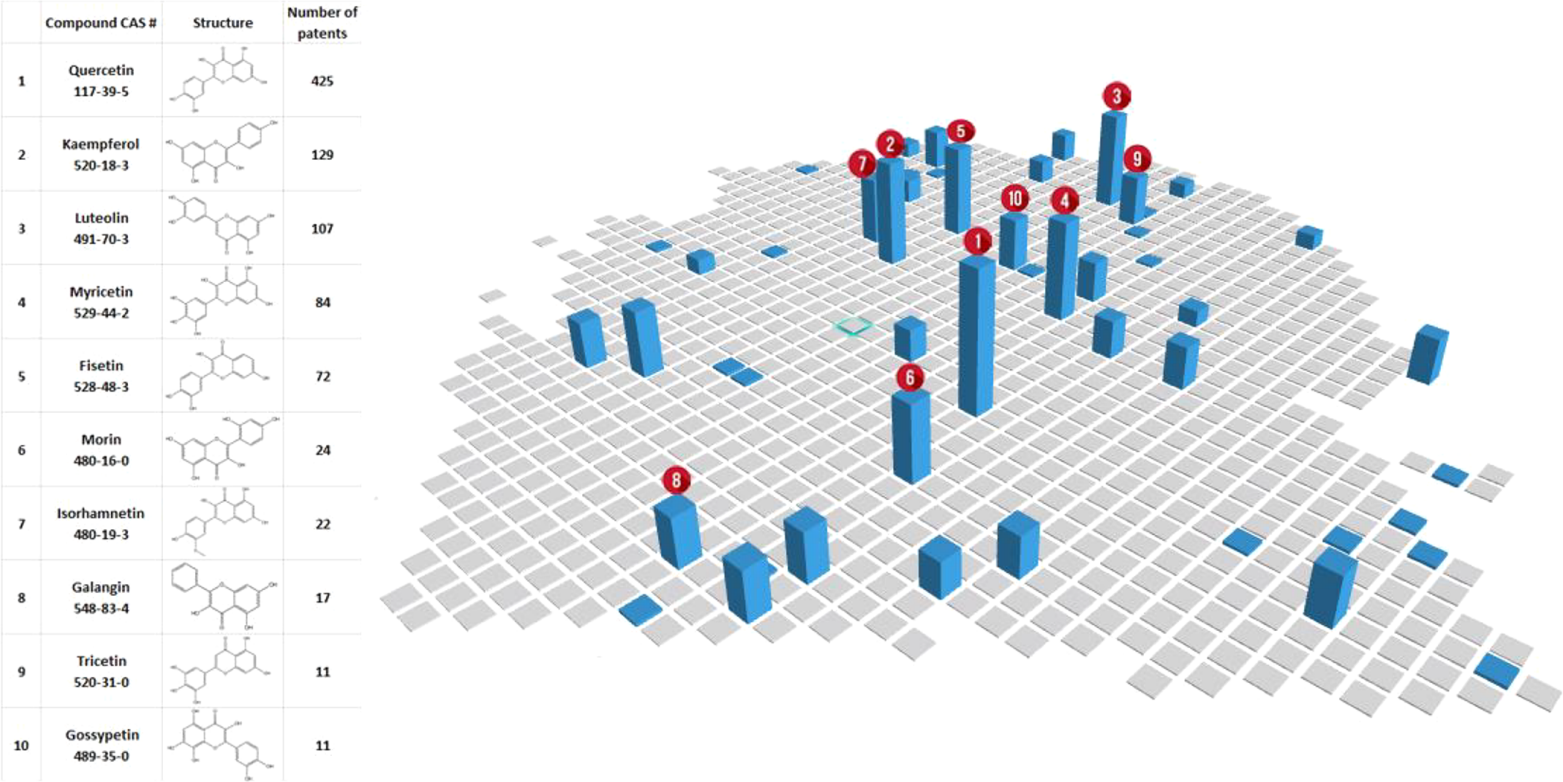
A search in SciFinder^n^ for compounds within the 90% similarity limit to quercetin used as an antiaging drug gave 51 compounds. Each column in the figure reflects a single compound, with the top 10 indicated by numbers. The distance between two columns reflects the similarity between these two compounds. The bar height reflects the number of patents related to a given compound. The figure was created using the ChemScape analysis tool of SciFinder^n^. The table on the left lists the top 10 compounds of this collection.

The initially identified potential senolytic drugs have been discovered using a hypothesis-driven approach,^284^ based on the observation that senescent cells resist apoptosis.^324^ The basic drug discovery hypotheses suggested that (i) senescent cells resist apoptotic stimuli and (ii) senescent cells are in certain aspects similar to cancer cells that do not divide.^284^ Further senolytic drug identification was bioinformatics-based^325^ and initially included the natural flavonoid **quercetin** (CAS RN 117-39-5) targeting senescent umbilical vein endothelial cells (HUVECs) and the tyrosine kinase inhibitor **dasatinib** (CAS RN 302962-49-8) targeting senescent primary adipocyte progenitor cells.^284^ Other members of this first generation senolytics class included **fisetin, luteolin, curcumin, navitoclax**, and others.^283,284,323,326^Figure 10 illustrates the use of the ChemScape tool within SciFinder^n^ ^297^ to search for compounds of similar chemical structure to quercetin as potential antiaging drugs. The collection included kaempferol, luteolin, myricetin, fisetin, morin, isorhamnetin, galangin, tricetin, gossypetin, and others (Figure 10). Currently examined senolytics include certain BCL-2 family inhibitors (e.g., ABT-737, navitoclax), HSP90 inhibitors (e.g., 17-DMAG (alvespimycin)), geldanamycin, 17-AAG (tanespimycin), STA-9090 (ganetespib), and p53-targeting compounds (e.g., FOXO4-DRI, UBX0101).^327^

Recently, a specific bioactive fraction of *Rhodiola* complexed with a marine lipoprotein extract from *Trachurus* sp. has been shown to markedly stimulate cell proliferation rate in cells under oxidative stress, imitating what has been observed in the aging process.^328^ This phytomarine complex with notable senolytic activity also considerably upregulates the vitagenes SIRT-1 and MMP2^328^ and might offer an innovative approach to avoid oxidative stress damage and prevent cell aging.^329^

The efficacy of the suggested senolytic drugs has been demonstrated in model organisms for age- and cellular senescence-associated physical disabilities, insulin resistance, cognitive decline, osteoporosis, osteoarthritis, and cancers.^284^ Senolytic agents have the potential to delay, prevent, or treat senescence- and age-related disorders, based on successful forthcoming human clinical trials. Other promising senolytic drugs include **acarbose**, **17-α-estradiol**, and **nordihydroguaiaretic** acid (NGDA).^319^ Recently, new structurally diverse compounds with promising senolytic activity have been identified using deep learning neural networks simulations.^330^ Lately, focus has been placed on natural senolytic compounds, which even though less potent than targeted senolytics, have the advantage of low toxicity.^331^ The natural polyphenolic compounds oleuropein and its metabolite hydroxytyrosol, epigallocatechin-gallate (EGCG), fisetin, piperlongumine, and wogonin are just a few of the known natural senolytics.^331,332^

### 4.3 Telomerase Activators

Therapeutic targeting of telomerase activity is another prospective antiaging approach. Since age-associated telomere shortening is known to play a key role in senescence, proper maintenance of telomeres is decisive for genome stability.^333,334^ Telomerase is the reverse transcriptase enzyme, which is able to sustain telomere length via telomeric repeat addition onto the chromosomes ends.^335^ Telomerase activation has been proposed to be an antiaging moderator, which can play a role in the aging-associated diseases therapy.

Several telomerase activator formulations have been examined as antiaging agents. Preliminary experimental data suggested that **TA-65** (cycloastragenol, CAS RN 78574-94-4), an extract of *Astragalus membranaceus* roots, can perform as a telomerase activator,^129^ but so far no sound clinical data have been reported. Another telomerase activator, **metadichol** (CAS RN 1627854-29-8), has been used to defeat organ failure by enriching cells with telomerase.^336^ Another study reported that natural formulations including *Centella asiatica* extract (08AGTLF) comprising >95% triterpenes, Astragalus extract, TA-65, oleanolic acid (CAS RN 508-02-1), maslinic acid (CAS RN 4373-41-5), and certain multinutrient formulations trigger significant increase in telomerase activity.^337^

### 4.4 Epigenetic Drugs

Epigenetic dysregulations associated with aging implicate intense health concerns for numerous pathologies including metabolic and cardiovascular diseases, psychiatric and neurodegenerative disorders, and cancer. Epigenetic drugs-based therapy has emerged as a potential strategy for treating certain aging-associated diseases.^338^ Epigenetic modifications are known to be reversible, which makes them suitable targets for pharmacological intervention.

An assortment of therapeutics have been developed recently targeting epigenetic regulation, including DNA methyltransferase modulators, histone deacetylase modulators, histone acetyltransferase (HAT) modulators, and noncoding miRNAs, exhibiting possible effects against age-related disorders.^339,340^ Among DNA methyltransferase modulators, **5-azacytidine** (azacytidine, CAS RN 320-67-2) and **5-aza-2′-deoxycytidine** (decitabine, CAS RN 2353-33-5) are the most thoroughly examined and demonstrate therapeutic potential against certain leukemias.^341,342^ Histone deacetylase inhibitors include several chemical groups: cyclic peptides, hydroxamic acids, short chain fatty acids, and benzamides.^343^ Experimental evidence shows significant life-extending potential of the histone deacetylase inhibitors such as 4-phenylbutyrate (PBA), trichostatin A, sodium butyrate, and suberoylanilide hydroxamic acid (SAHA).^344,345^ A wide range of histone deacetylase inhibitors are emerging as potential anticancer medications, including belinostat, panobinostat, SAHA and FK228,^346^ trichostatin A, sodium butyrate, vorinostat, valproic acid, and romidepsinor^347^ demonstrating considerable activity in hematological and solid tumors.

### 4.5 Antioxidants

According to the oxidative damage theory of aging,^6^ free radicals and other reactive oxygen species (ROS), developed throughout mitochondrial metabolism, may end up producing impaired molecules including carbonylated proteins, lipid peroxides, and oxidized DNA,^348−353^ the accumulation of which is supposedly the primary cause of cellular senescence, age-related telomere attrition and diseases.^351,354^ Accumulated ROS excess can be allegedly destroyed by the endogenous physiological antioxidative systems including the enzymes superoxide dismutase (SOD), glutathione reductase, catalase, and glutathione peroxidase, which help to keep the balance between oxidative and antioxidative processes. In terms of chronic oxidative stress, however, the endogenous antioxidant systems happen to be insufficiently effective, and it has been believed that administration of certain exogenous antioxidants such as vitamins E and C, curcumin, melatonin, β-carotene, lipoic acid, coenzyme Q10, glutathione, polyphenols, phytoestrogens, as well as certain minerals including zinc, manganese and selenium, can be a factor in maintaining homeostasis.^283^

**Vitamin E** (CAS RN 1406-18-4) comprises a group of several fat-soluble compounds including tocopherols and tocotrienols, of which **α-tocopherol** is the most potent antioxidant form of vitamin E. Due to their lipophilic features, they can be found in lipoproteins, cellular membranes, and fat deposits. Vitamin E is the key defender against free radical effects. It is stored in the liver and fat cells and protects cellular components and especially cellular membranes from damage.^355,356^ Vegetable oils such as sunflower, soybean, and safflower oils are some of the best sources of vitamin E.

**Vitamin C** (l-ascorbic acid, CAS RN 50-81-7), a water-soluble vitamin naturally present in citrus and other fruits and vegetables, is an electron donor and a versatile free radical scavenger. It has been found to regenerate other antioxidants, including α-tocopherol (vitamin E) from the tocopheryl radical.^357^ It is a particularly efficient antioxidant because of its high electron-donation power and ready conversion back to the active reduced form. Vitamin C is necessary for the biosynthesis of collagen, l-carnitine, and some neurotransmitters. It is also a participant in protein metabolism^358,359^ and plays an significant role in immune function.^357^

**Curcumin** (CAS RN 458-37-7) is the key active component of turmeric (*Curcuma longa*) root. It has been shown to exhibit antioxidant, anti-inflammatory, antineurodegenerative, and antitumor activities.^360,361^ Recently it has been implied that curcumin upregulates SIRT3 expression in skeletal muscle tissues,^362^ as well as in the brain after stroke.^363^ Numerous other dietary **polyphenols** such as **resveratrol** and **(−)-epigallocatechin-3-gallate** (EGCG) have been shown able to mitigate age-produced cellular damage via metabolic formation of ROS, via specific cell-signaling actions that may stimulate SIRT1 activity. Furthermore, polyphenolic compounds have proven inhibitory activity against chronic vascular inflammation related to atherosclerosis.^364^

**Melatonin** (*N*-acetyl-5-methoxy tryptamine, CAS RN 73-31-4) is a hormone secreted by the pineal gland and regulates sleep/wake cycles. It is a well-recognized antioxidant able to support healthy aging. The endogenous melatonin levels have been found to decrease upon aging and even stronger in certain neurodegenerative disorders, especially Alzheimer’s disease, as well as in type 2 diabetes.^365^ It has been reported relevant to the attenuation of inflammaging in the brain.^365^ Studies have documented the ability of melatonin to increase SIRT levels against brain aging.^366,367^

**β-Carotene** (CAS RN 7235-40-7) is a carotenoid, a micronutrient with numerous physiological functions. It is an antioxidant, which has been shown to inhibit the incidence and development of cancer. It also has an anti-inflammatory effect in various animal and cell models.^368^ Its antiaging efficacy has been documented *in vitro*, using mesenchymal stem cells, as well as *in vivo*, in model animals.^369^ It was implied to inhibit aging by regulating the KAT7-P15 signaling axis.^369^ Other carotenoids, including α- and γ-carotenes, lycopene, lutein, β-cryptoxanthin, and zeaxanthin, are also known as effective natural antioxidants. Synergistic action of vitamins C and E, and carotenoids, has been reported to successfully prevent lipid peroxidation.^370^

**α-Lipoic acid** (6,8-thioctic acid, CAS RN 1200-22-2) is a dithiol that acts as a coenzyme factor for certain redox reactions. It is synthesized in animals naturally and is essential for aerobic metabolism. Its efficiency in diseases associated with aging-provoked oxidative stress has been documented, so its dietary supplementation has been found beneficial.^371−373^ Lipoic acid has been also shown to activate SIRT1 and SIRT3 in peripheral tissues, thus improving mitochondrial function and protecting against cardiac hypertrophy.^374,375^

**Coenzyme Q10** (CoQ10, CAS RN 303-98-0), a powerful antioxidant known to offer a variety of benefits to support healthy aging, is naturally produced in the body and is participating in energy production. It has attracted large-scale interest due to its crucial role in mitochondrial bioenergy, antioxidation, antiaging, and immune system regulation. Upon aging, the production of CoQ10 declines, so it is necessary to supply additional amounts through foods such as meats, fatty fish (e.g., trout) and nuts (e.g., pistachios) or through dietary supplements. It has been reported to exert positive effects in enhancing age-produced deterioration of oocyte quality.^376^ CoQ10 and selenium supplementation have been reported to increase serum sirtuin1 levels^377^ and to improve heart function in the elderly.^378^

l**-Glutathione** (GSH, CAS RN 70-18-8) is an abundant antioxidant playing an essential role in protecting cells against oxidative stress-caused cellular damage. Declined glutathione levels are related to the typical characteristics of aging, as well as of a wide variety of pathological conditions, including neurodegenerative disorders. Glutathione depletion seems to be important for the onset of Parkinson’s disease.^379^ Furthermore, glutathione is important for body detoxification, and it is also a successful immunostimulant and skin rejuvenator.^380^

**Fermented Papaya Preparation** (FPP), a product resulting from yeast fermentation of *Carica Papaya*, is a powerful antioxidant and nutraceutical adjuvant.^381^ Its major reported actions are as a free radical regulator,^382^ immunomodulator,^383,384^ and antioxidant.^385,386^ An animal model study has indicated that daily FPP assumption caused an increase in telomeres length in bone marrow and ovary, along with an increase in the plasmatic levels of telomerase activity and the antioxidant levels, accompanied by a decrease of ROS.^381^

#### Antioxidant Intake Controversy

The reported inverse correlation between systemic levels of antioxidants and certain age-associated diseases has resulted in the perception that antioxidant supplementation is an effective prophylactic and therapeutic intervention for such aging pathologies. However, the therapeutic results of this strategy in clinical trials have been frequently disappointing.^387^ The interplay of both endogenous and exogenous antioxidants is complex and still not well understood. To successfully maintain the redox homeostasis, exogenous and endogenous antioxidants need to act synergistically.^388^ A fine-tuned equilibrium between the oxidative and antioxidative processes in the organism is vital in preserving homeostatic stability. Excessive antioxidant supplementation might turn out to be damaging for the delicate homeostatic control mechanisms resulting in health decline.^389^ Similarly to the hormesis-causing agents, antioxidants perform beneficially in certain concentration range, and their higher concentrations are typically toxic to organisms. The excessive levels of exogenous antioxidants may perturb the endogenous signaling pathways and thereby be harmful.^390^ In view of these contradictions, it is not surprising that conflicting data have been reported on the health outcomes of long-term antioxidant intake. This ambiguity is often referred to as “antioxidant paradox”.^283^ While benefits of dietary antioxidant supplementation appear to be clear in cases of high oxidative stress and endogenous antioxidant insufficiency, further research is necessary to clarify the potential risks and advantages linked to the supplement of antioxidants by healthy people.

### 4.6 Antiaging Peptides

Peptides have been mainly used in antiaging cosmetics and cosmeceuticals to repair skin aging signs like wrinkles and sagging. They have been found effective also at hair regrowth stimulation as well as weight loss.^391−393^ Furthermore, peptides were reported useful for treating rheumatoid arthritis^394^ and as analgesics.^395^ Peptide supplementation modified energy metabolism and oxidative stress, improved endurance, and decreased fatigue in experimental animals.^396^ Because of their hydrophilicity, peptides for use in skin cosmetics are commonly lipidated by esterification with an alkyl (most often palmitoyl) chain to enhance their penetration through the highly lipophilic stratum corneum. Exemplary antiaging peptides are shown in Table 2.

**Table 2:**
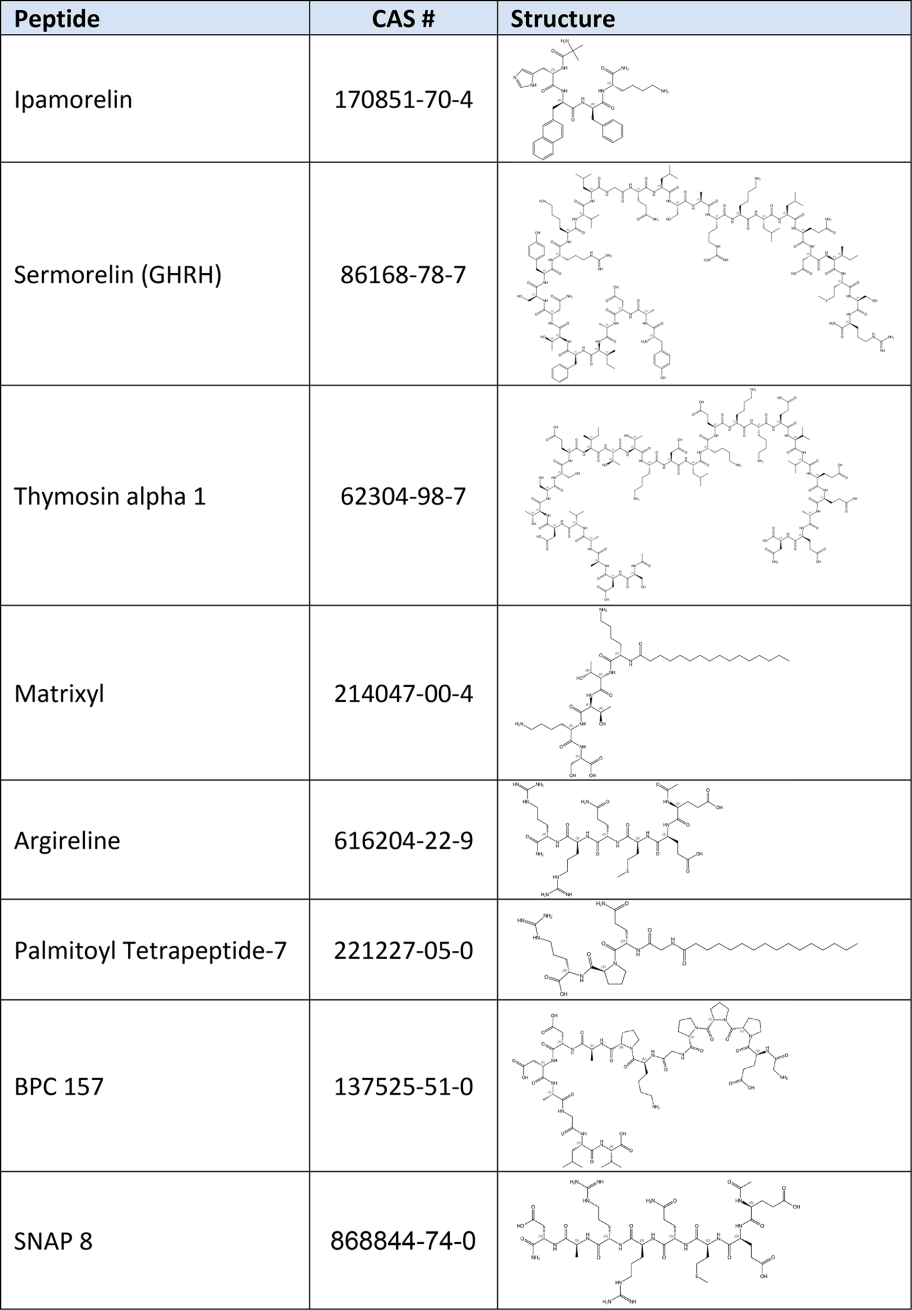
Exemplary Antiaging Peptides

**Figure 11:**
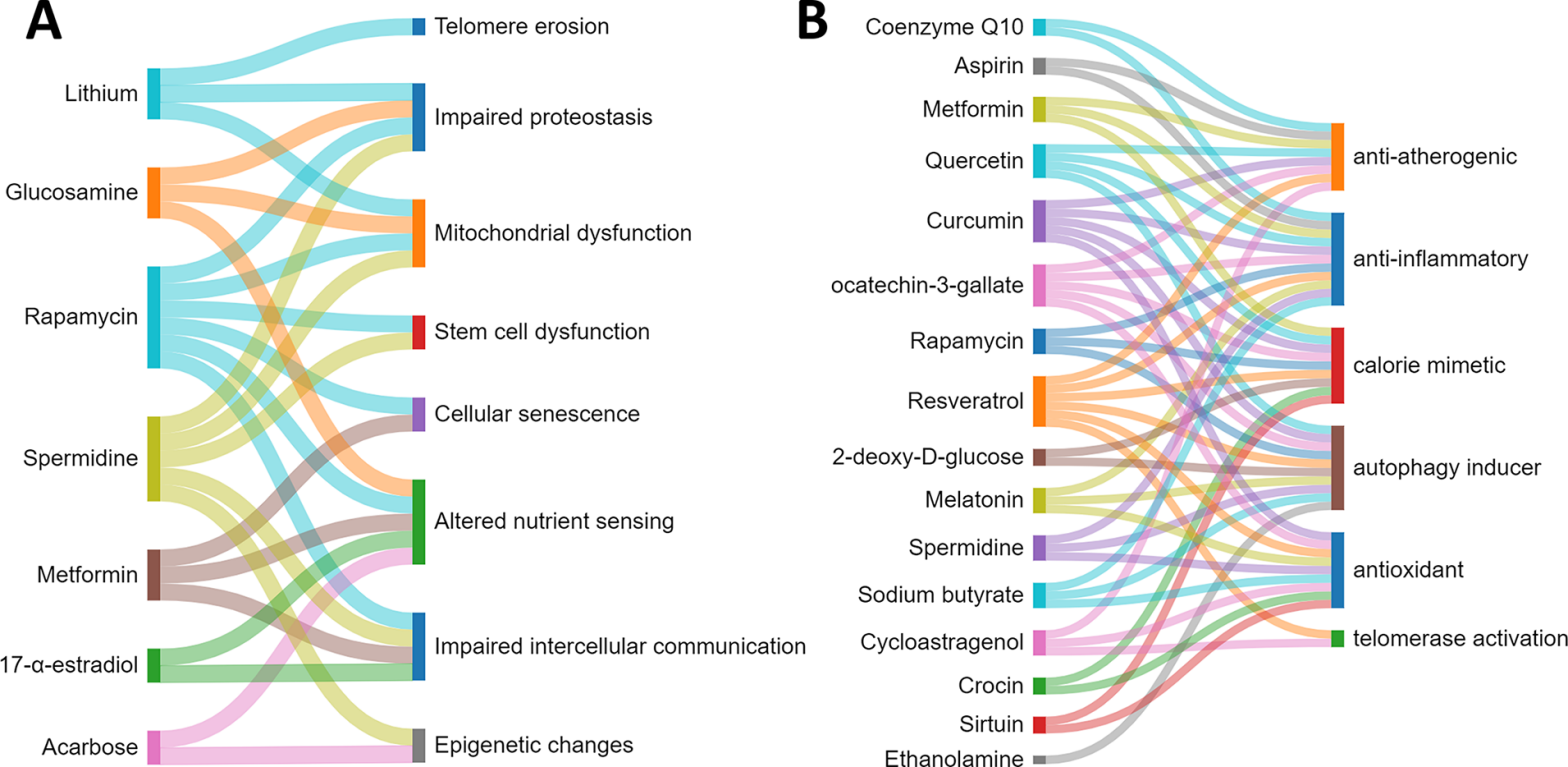
Correlations between exemplary antiaging drugs and (A) hallmarks of aging, and (B) antiaging strategies.

The mechanism of action of many antiagent agents is often related to multiple aging hallmarks and refers to several antiaging strategies. In Figure 11 we have exemplified certain correlations between some antiaging drugs, hallmarks of aging (A), and antiaging strategies (B).

In Tables 3 and 4 we have summarized the natural (Table 3) and synthetic (Table 4) antiaging agents most widely represented in the CAS Content Collection,^45,283,288−291,397−408^ complete with their chemical structures and the number of documents (journal articles and patents) in the CAS Content Collection, in which their antiaging performance has been documented and discussed. A more extensive collection of antiaging compounds represented in CAS Content Collection is provided in the Supporting Information, Tables S1 and S2.

**Table 3:**
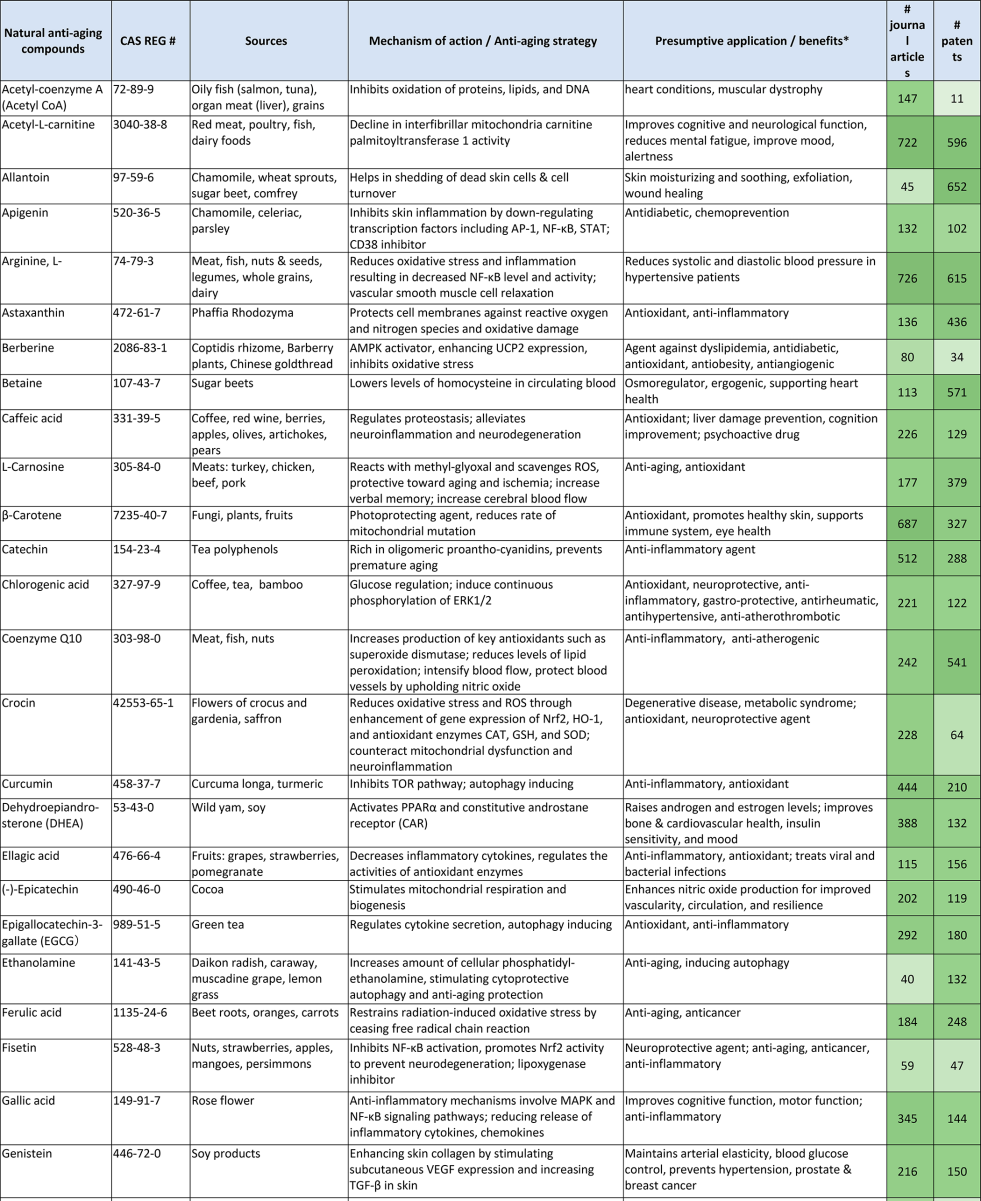

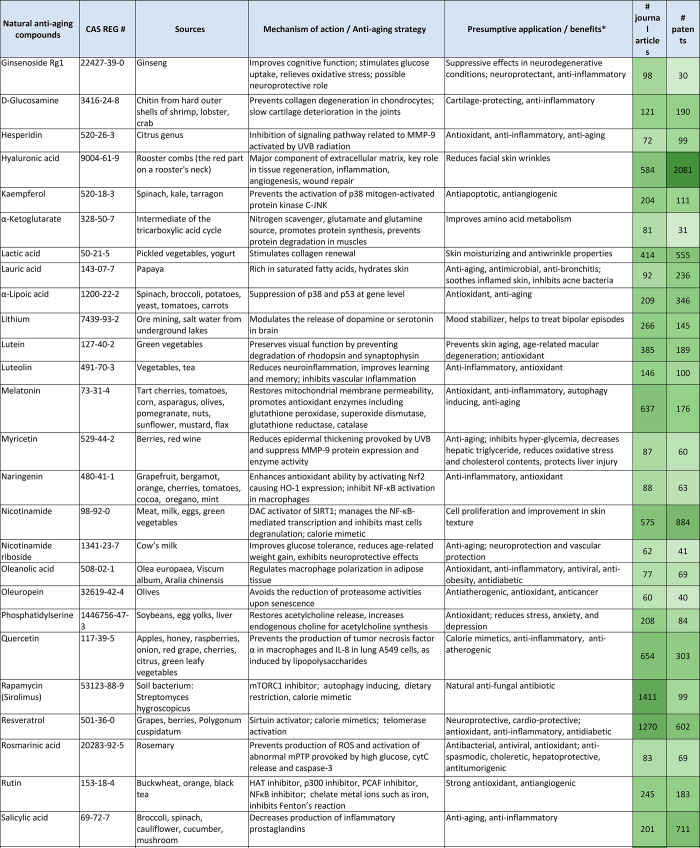

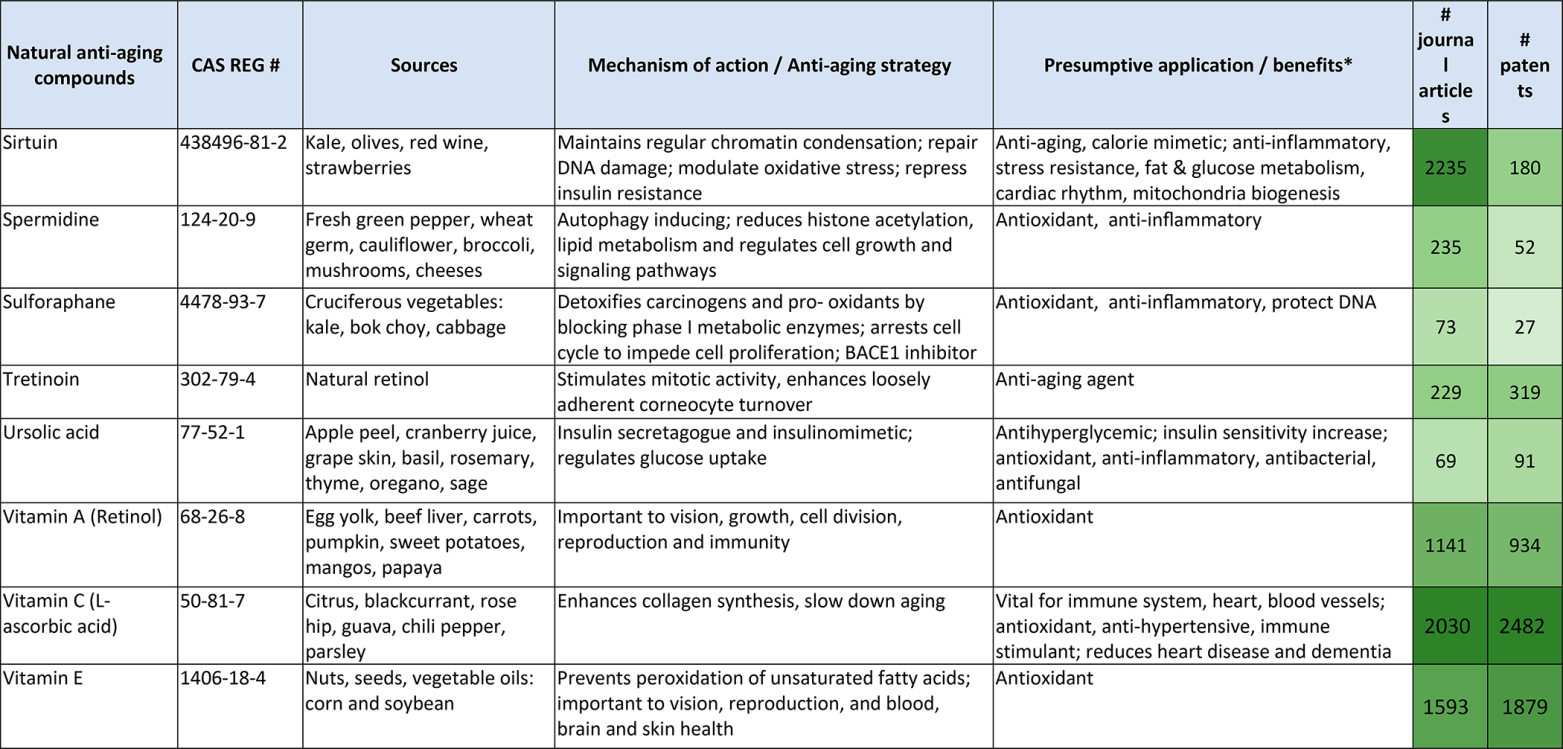
Natural Antiaging Agents Most Widely Represented in the CAS Content Collection^a^^,^^45,283,288−291,397−408^

a The asterisk indicates the listed benefits should be regarded as presumptive and not as robustly, clinically proven benefits.

**Table 4:**
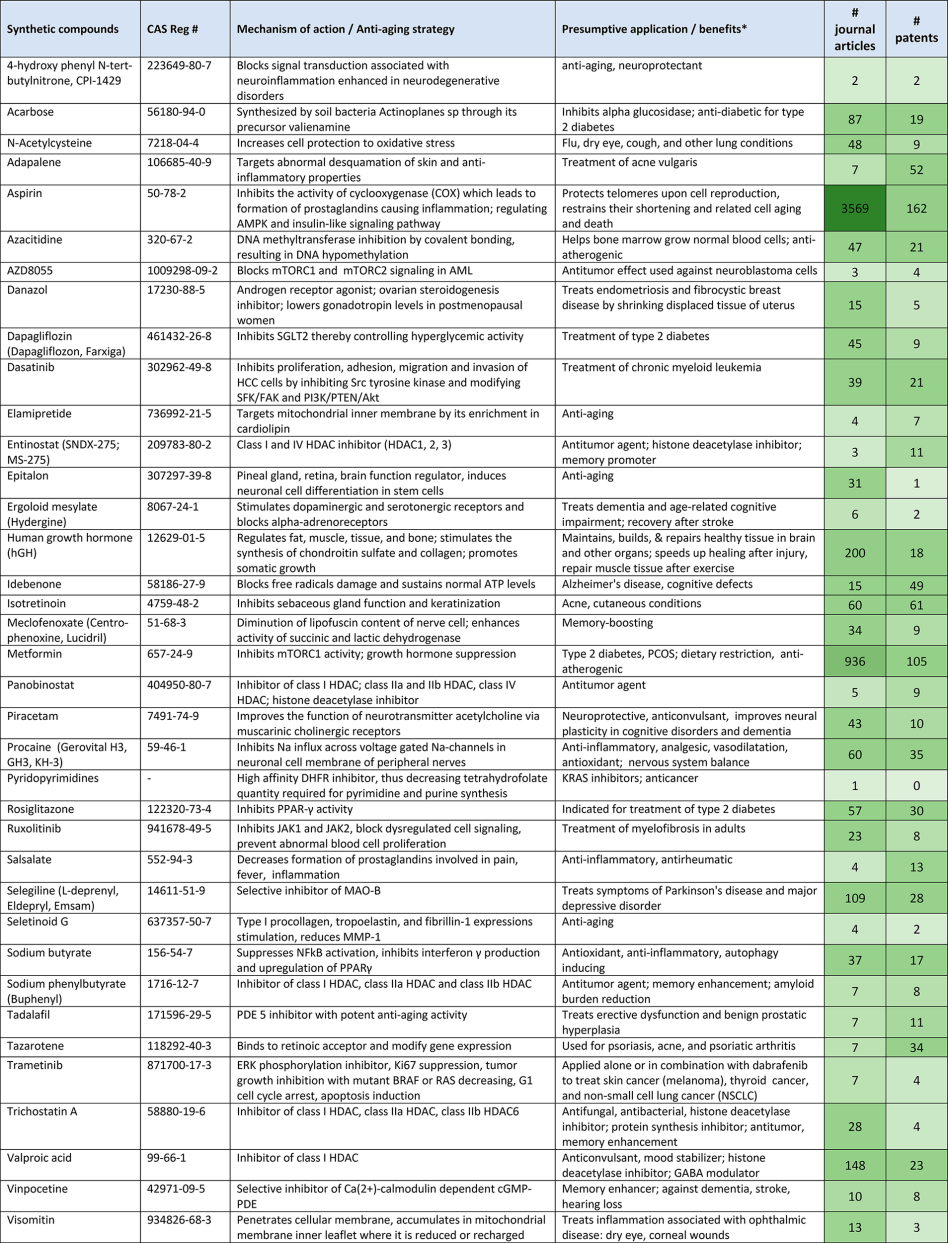
Synthetic Antiaging Agents Most Widely Represented in the CAS Content Collection^a^^,^^45,283,288−291,397−408^

a The asterisk indicates the listed benefits should be regarded as presumptive and not as robustly, clinically proven benefits.

## 5 Private Investment

**Figure 12:**
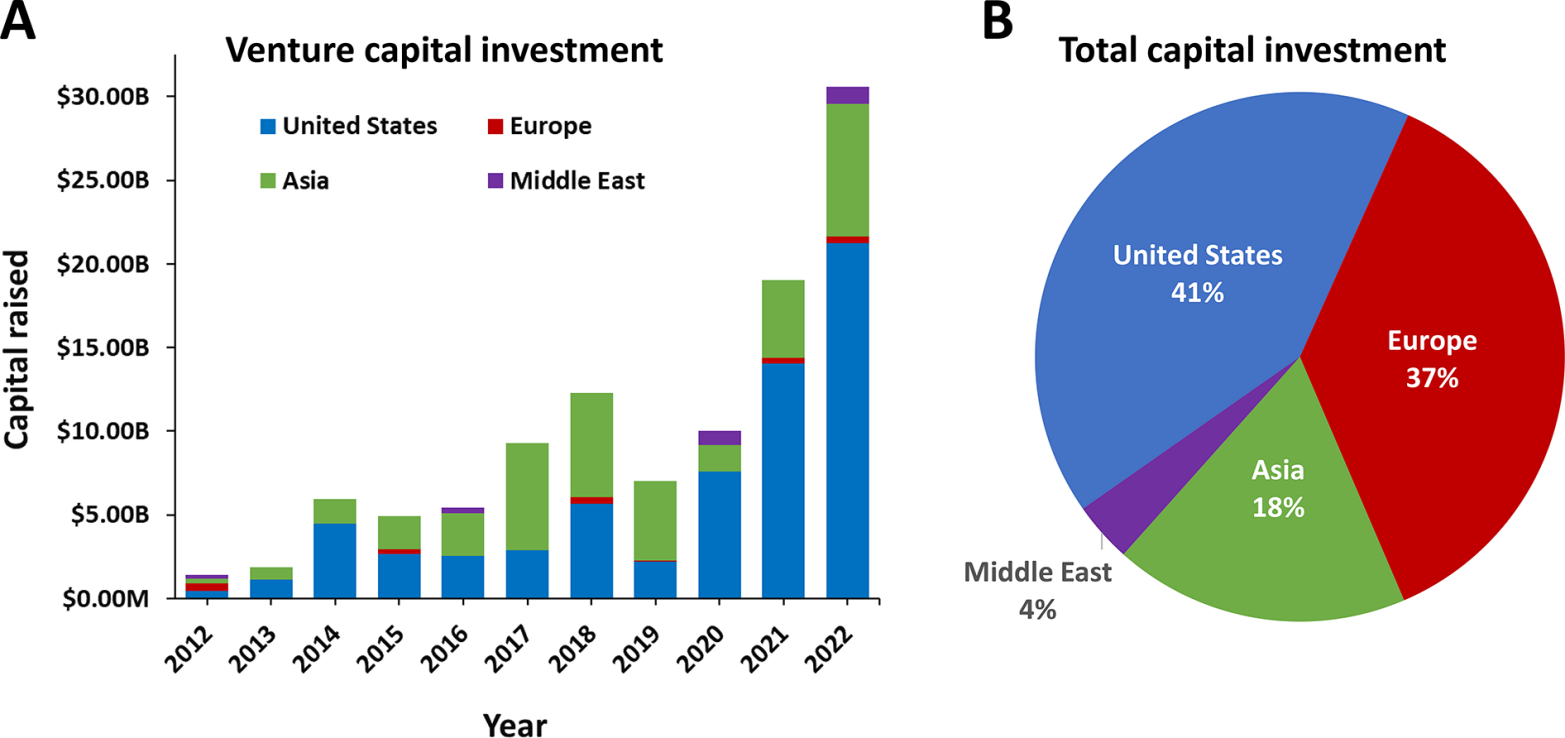
Overall capital raised of (A) venture capital investment and (B) total capital investments for the period 2012–2022 in the antiaging field [$] distributed by regions. Source: PitchBook.com.

Examining the overall global private investment activities of the antiaging field provides insight into the commercial interest into this area. Performing a search of antiaging within PitchBook,^409^ an online source for investment data, reveals the overall venture capital activities. Antiaging field refers to companies, which perform research and development of restorative treatments to prevent or treat the effects of aging and enhance lifespan. Research areas include genomic instability, telomere attrition, epigenetic alteration, loss of proteostasis, impaired nutrient sensing, mitochondrial dysfunction, cellular senescence, stem cell exhaustion, and altered intercellular communication. The search revealed capital steadily raised within this industry (Figure 12A).^409^ From 2012 through 2018, the total venture capital raised increased from $1.4B to over $12.2B. 2019 revealed the overall venture capital raised fell to just $7.0B. Capital raised continued to grow for 2019 to 2022 totaling over $30.6B in 2022 in total venture capital investment (Figure 12A). In the years 2013–2015 and 2020–2022 the investments were dominated by the United States, while in 2016–2019 investments from Asia were dominating. The venture capital investment data in this area clearly show a recent and increasing commercial interest surrounding antiaging agents, revealing its potential promise for therapeutic applications.

In addition to the venture capital investments, in 2020 there was an investment of over $97B from the European Union, for the Horizon 2020 SME Instrument.^410^ The SME Instrument supports high-risk and high-potential small and medium-sized initiatives to develop and provide new products, services, and business models able to drive economic growth. This large investment enhanced significantly the Europe contribution in the total capital investments distribution (Figure 12B).

## 6 Clinical Trials

**Figure 13:**
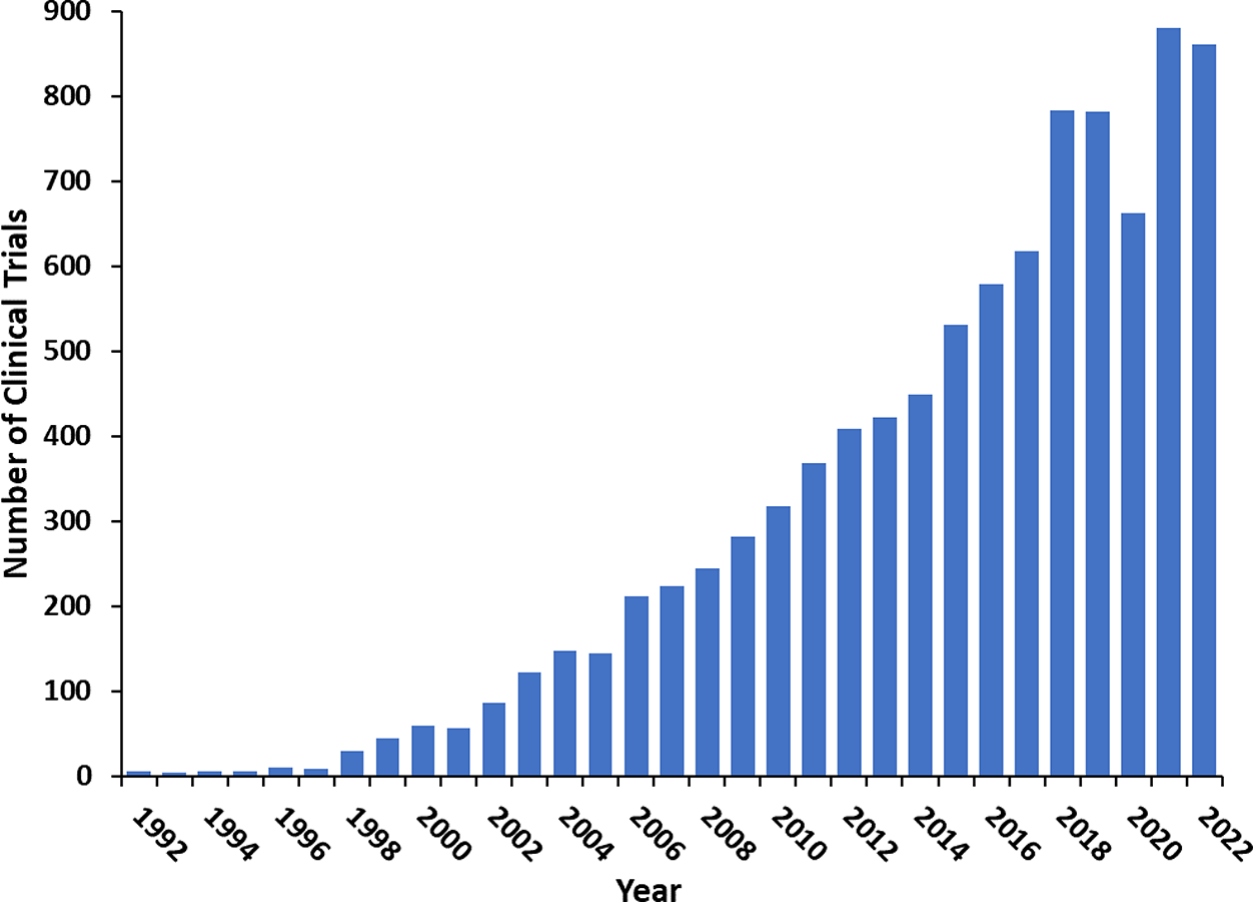
Number of therapeutic antiaging clinical trials by year.

A representative selection of therapeutic antiaging clinical trials is examined within this section to gain an overall view of the past, present, and future state of clinical development. A selection of the top 10 000 antiaging clinical trials^411^ from https://clinicaltrials.gov are examined against time, clinical trial phase, status, disease indication, and antiaging strategy. Antiaging therapeutics are well established in clinical development, with Figure 13 showing a steady growth starting in the early 1990s and continuing through 2022.

**Figure 14:**
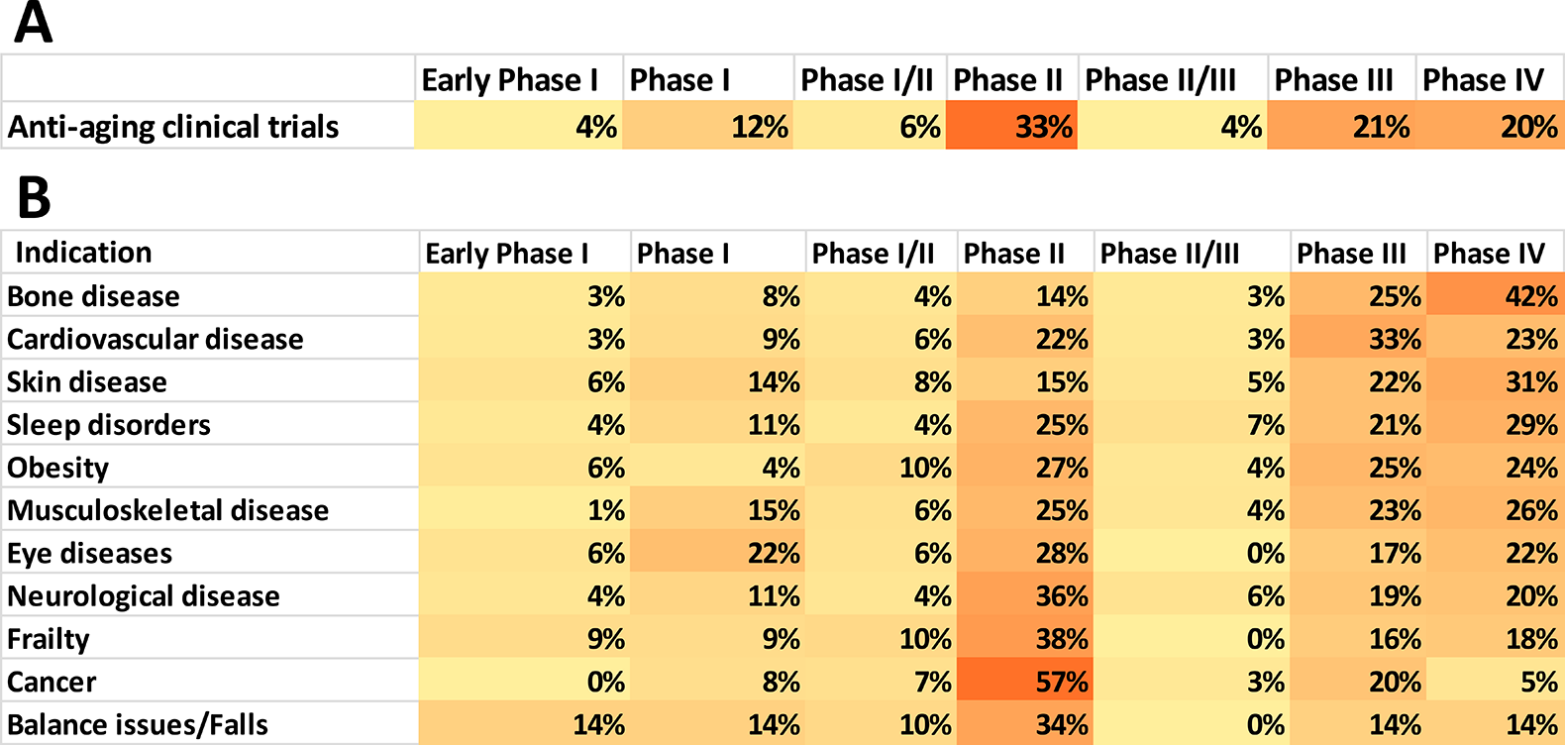
Percentage of therapeutic antiaging clinical trials in various phases for the treatment of age-related disease indications: (A) overall antiaging clinical trial phase development; (B) clinical trial phase development for specific age-related diseases.

Analysis of therapeutic antiaging clinical trial phases reveals that close to half of all trials are in phases III and IV with the other half filtering into earlier phases. Phase II trials contain the highest percentage of all categories encompassing 33% of all trials (Figure 14A). Examining clinical trials a step further, by disease indication, shows that bone, cardiovascular, and skin diseases along with sleep disorders and obesity are well established in the development pipeline having the highest percentage of clinical trials further along in phase III and phase IV clinical trials (Figure 14B). Balance disorders, cancer, frailty, along with eye and neurological disease are the indications less established in the pipeline with the largest percentage of trials in earlier phases (Figure 14B).

**Figure 15:**
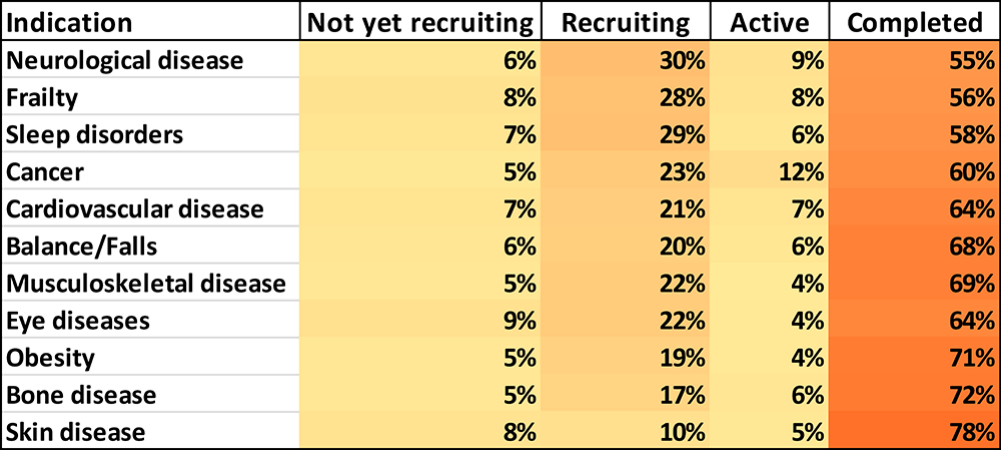
Percentage of therapeutic antiaging clinical trials in various statuses for the treatment of age-related disease indications.

Next, we review therapeutic antiaging clinical trial statuses, characterized by age-related disease indications. Current therapeutic antiaging clinical trials in early phase trials such as neurological disease, frailty, sleep disorders, and cancer also have the highest percentage in active, recruiting, and not yet recruiting statuses (Figure 15). These early phase trials are active or getting ready to be active in the pipeline. On the other hand, it is no surprise that disease indications more well established in the development pipeline such as skin, bone, and eye diseases along with obesity also contain the highest percentage of completed trials. There is however still current advancement in these areas reflected by greater than 20% of their trials in active, recruiting, or not yet recruiting status (Figure 15).

Finally, representative clinical trials examining antiaging therapeutics are highlighted in Tables 5–12 categorized by antiaging strategy. These are examined in further detail below to showcase a variety of antiaging strategies, interventions, and targeted conditions in clinical development along with their status and any published results.

**Table 5:** Highlighted mTOR Inhibition Antiaging Clinical Trials

indication	intervention	status	sponsor	NCT no.
Geographic atrophy	Rapamycin	Complete	National Eye Institute, USA	NCT01445548
Aging frailty	Rapamycin	Complete	Mayo Clinic, USA	NCT01649960
Aging/epigenetics/inflammatory mediators	Rapamycin	Active, not recruiting	The University of Texas Health Science Center, USA	NCT04608448
Age-related sarcopenia	Rapamycin|resistance exercise	Recruiting	University of Nottingham, U.K.	NCT05414292

**mTOR inhibition** is widely explored in clinical trials for antiaging therapeutics (Table 5). The National Eye Institute researched the use of rapamycin for the treatment of geographic atrophy (GA) as part of late-stage age-related macular degeneration (AMD) in phase I/II clinical trial NCT01445548. Six participants with bilateral GA were enrolled and received intravitreal sirolimus, but no benefit was detected.^412^ A following phase II study NCT01675947 enrolled 52 participants with GA for monthly intravitreal sirolimus treatment. The study was terminated early due to lack of efficacy and the adverse event of sterile endophthalmitis in three participants.^413^ It was determined that while immunosuppression may play some role in AMD, it might not be the main pathway for GA development. The Mayo Clinic also researched at the use of rapamycin in phase I clinical trial NCT01649960. They focused on rapamycin treatment effects on senescence markers and frailty in elderly subjects undergoing cardiac rehabilitation. Thirteen participants received low doses (0.5–2 mg) of rapamycin daily for 12 weeks. While correlation between some senescence markers and physical performance were observed, the primary end point measurement of frailty saw no improvement.^414^

mTOR inhibition research continues however with the University of Texas Health Science Center at San Antonio currently investigating the use of rapamycin for inflammation reduction and epigenetic reversal in an active clinical trial (NCT04608448). Subjects aged 65–95 will topically apply rapamycin 8% ointment to a forearm daily for 6 months. Both epigenetic and inflammatory markers will be measured. The University of Nottingham is recruiting for another study (NCT05414292) looking at the use of rapamycin and resistance exercise on age related muscle loss. The study aims to recruit 16 healthy male participants over 50 years old who will take a 1 mg rapamycin oral tablet daily for 16 weeks along with performing a 14-week unilateral resistance exercise program. Measurements such as changes in muscle mass, strength, power, and function will be recorded.

Targeting aging senescent cells to combat aging with **senotherapy** is currently undergoing research in clinical trials (Table 6). One such study researched the use of Carlson fish oil to improve immune senescence biomarkers CD28, CD57, on the surface of CD4+ and CD8+ T lymphocytes in aging participants 40–70 years old with HIV infection. The intervention groups received 1.6 g of ω3 fatty acids daily for 12 weeks.^415^ Published results were less than encouraging however with no significant difference in immune senescence measurements in participants.^416^ Another phase I/II clinical trial NCT04063124 researching targeting aging senescent cells was recently completed by the University of Texas Health Science Center at San Antonio. Five early stage AD patients were enrolled and received 100 mg of dasatinib and 1000 mg of quercetin every 2 weeks for 12 weeks. The outcome measurements of brain penetration for both compounds and AD and senescence biomarkers were collected.^417^ Interpretative results have yet to be published, but the results of this pilot study will guide researchers in developing a larger phase II trial researching senolytic agents for the modulation of Alzheimer’s disease progression.^418^ Lastly, we examine phase II clinical trial NCT04733534 currently recruiting 60 participants. St. Jude Children’s Research Hospital will evaluate two senolytic regimens for their cellular senescence biomarker reduction and frailty improvement in adult survivors of childhood cancer. Treatment groups will receive either oral dasatinib (100 mg/day) and quercetin (500 mg twice daily) on days 1, 2, 3, 30, 31, and 32 or oral fisetin (20 mg/kg/day) on days 1, 2, 30, and 31. Outcome measurement of walking speed and blood senescent cells (p16INK4A) levels will be recorded. If this pilot study is successful, it too will provide evidence needed for a continued phase II trial to determine further efficacy.^419^

**Table 6:** Highlighted Senotherapy Antiaging Clinical Trials

indication	intervention	status	sponsor	NCT no.
Immune senescence/inflammation	Fish oil	Complete	Rush University Medical Center, USA	NCT02102724
Alzheimer’s disease	Dasatinib and quercetin	Complete	The University of Texas Health Science Center, USA	NCT04063124
Frailty/childhood cancer	Dasatinib and quercetin|fisetin	Recruiting	St. Jude Children’s Research Hospital, USA	NCT04733534

**Hormonal replacement** trials in the clinical pipeline have shown promising results (Table 7). A large phase III clinical study (NCT00799617) sponsored by the University of Pennsylvania called the testosterone trials is a coordinated set of seven clinical trials including 788 male participants with a mean age of 72. The treatment group applied 5–15 g of AndroGel once daily for 12 months to determine the efficacy of increasing the testosterone levels of older men with low testosterone.^420^ Results from these trials are highlighted below.^421^

- Testosterone Level: increased the median testosterone level from low to normal
- Sexual Function Trial: increased sexual activity, sexual desire, and erectile function
- Physical Function Trial: increased distance walked
- Vitality Trial: slight increase in mood and depression; did not increase energy
- Anemia Trial: increased hemoglobin
- Bone Trial: increased volumetric bone mineral density and strength of the spine and hip bones
- Cardiovascular Trial: increased coronary artery noncalcified plaque

**Table 7:** Highlighted Hormonal Replacement Antiaging Clinical Trials

condition	intervention	status	sponsor	NCT no.
Andropause	Testosterone	Completed	University of Pennsylvania, USA	NCT00799617
Recurrent urinary tract infection	Estrogen	Completed	University of California, San Diego, USA	NCT01958073
Atherosclerosis	Bazedoxifene/conjugated estrogen	Recruiting	University of Southern California, USA	NCT04103476

The testosterone trials have shown that increasing testosterone levels in older men with low testosterone has documented benefits. This trial paves the way for larger trials to explore the risks and efficacy deeper and has helped influence physician decisions regarding testosterone treatment in older men.

The University of California, San Diego researched the use of estrogen with two separate drug delivery systems for the prevention of recurrent urinary tract infections (UTIs) in postmenopausal women. Their phase IV clinical trial NCT01958073 had an intervention of 0.5 g of estrogen vaginal cream 2 times weekly or an estradiol ring every 3 months for 6 months.^422^ More encouraging results were revealed in this trial with vaginal estrogen preventing UTIs in postmenopausal women who have been diagnosed with recurrent UTIs.^423^ The University of Southern California is recruiting for its phase II trial NCT04103476 which will look at the effects of a tissue selective estrogen complex therapy on the progression of atherosclerosis and cognitive decline in 360 postmenopausal women aged 45–59 years. The treatment group will receive bazedoxifene 20 mg/conjugated equine estrogen 0.45 mg for up to a total of 3 years. Measurement outcomes will include carotid artery intima-media thickness, arterial stiffness, and three composite cognitive measures to determine cognitive decline.^424^

Aging induces various gut microbiota changes especially the reduction in health promoting species. Researchers in the clinic are looking at **gut microbiota modulation** for disease treatment of various age-related indications (Table 8). A few of these studies examined the use of prebiotics and probiotics along with fecal microbiota transplant (FMT) to aid a variety of conditions such as immune function, skin health, infection, and neurological disease. One such study sponsored by Clasado discovered that their Bimuno galacto oligosaccharide (GOS) mixture product has the potential to increase *Bacteroides* and *Bifidobacterium* fecal bacteria during an early phase I clinical trial (NCT01303484). Forty participants (65–80 years old) with a dose group of 5 g/day of GOS mixture for 10 weeks revealed that supplementation with a GOS prebiotic positively affects the gut microbiota and biomarkers for immune function among the elderly.^425^

**Table 8:** Highlighted Gut Microbiota Modulation Antiaging Clinical Trials

antiaging strategy	indication	intervention	status	sponsor	NCT no.
Prebiotics	Immunosenescence	Bimuno galacto-oligosaccharide	Complete	Clasado, U.K.	NCT01303484
Probiotics	Respiratory tract infections	*Lactobacillus casei Shirota*	Complete	University Antwerp, Belgium	NCT00849277
FMT	Parkinson’s disease	FMT	Complete	University Ghent, Belgium	NCT03808389
Probiotics	Inflammation	*Bifidobacterium adolescentis* Bif-038	Recruiting	Chr Hansen, Denmark	NCT05529693

On the other hand, the University of Antwerp, Belgium, saw no immune improvement when looking at the use of *Lactobacillus casei Shirota* (LcS) probiotic for the prevention of respiratory infection and immune boost in elderly nursing home residents (NCT00849277). 737 volunteers aged 65 and older were enrolled and the treatment group received a daily fermented milk drink that contained greater than 6.5B live LcS cultures for 176 days with a flu vaccine given on day 21. The results showed no significant effect on the protection against respiratory infections or regarding flu vaccine immune response.^426^

Currently in the pipeline, Chr Hansen is recruiting for their research (NCT05529693) on the use of *Bifidobacterium adolescentis* Bif-038 on low grade inflammation biomarkers. Subjects aged 65–85 years old with low and high treatment groups of 1 and 10 billion CFU for 12 weeks will be tested and various biomarkers such as C-reactive protein and TNFα will be measured.^427^ Another method of gut microbiota modulation, FMT, is currently only approved for the treatment of recurrent *Clostridioides difficile* infection, but researchers are branching out and researching its use for other indications with a gut–brain access connection. The University of Ghent has recently completed a clinical trial researching the effect of nasojejunal FMT (NCT03808389) on subjects with Parkinson’s disease. 49 subjects aged 50–65 were enrolled, and the treatment group received donor fecal microbiota with published results forthcoming.^428^

**Caloric restriction** is well researched as an antiaging strategy with its clinical development widely established. The 2-year clinical trial NCT00427193 by Duke University enrolled over 200 people to research the effect of a 25% calorie restricted diet on aging and age-related disease processes. Outcome measures included change in core body temperature, resting metabolic rate, inflammatory marker TNFα, along with fat mass.^429^ The effect of calorie restriction included significant decreases in both inflammatory markers and cardiometabolic risk factors which suggests potential benefits for aging and age-related disease processes.^430^ The University of Alabama also researched the use of caloric restriction along with exercise for the reduction of cardiometabolic risk. Phase III clinical trial NCT00955903 enrolled 167 participants with outcome measurements of change in abdominal fat mass, cardiometabolic risk factors, and weight change. Study results show significant improvement to relative fat mass, biomarker adiponectin and leptin, and cardiometabolic risk measurements for the intervention arm.^431^

Researchers are also currently investigating how caloric restriction can affect biological aging and neurodegenerative diseases such as cognitive impairment and Alzheimer’s disease (Table 9). TruDiagnositic is conducting an active phase II trial with 50 subjects enrolled to investigate the use of Peak Human Labs calorie mimetic supplement along with a fasting mimicking diet to see their effect on biological aging. The intervention group will take the supplement for 90 days mixing in a 5-day fasting diet, three times. Outcome measurements include the epigenetic age biomarkers which will test methylation at 850 000 locations on the DNA and body max index.^432^ Another active study performed by the University of Kansas hopes to learn how the Mediterranean diet compared to a low-fat diet for 12 months affects cardiometabolic biomarkers, brain antioxidant status, brain volume, and memory in cognitive normal adults aged 65 or greater. Researchers plan to examine brain processes to understand health and how the Mediterranean diet may help treat Alzheimer’s disease in the future.^433^ The University of Genova is also currently recruiting for its phase I/II study which will investigate the use of a specific 5-day low protein fasting diet called Prolon ADTM. 40 participants will be enrolled, and the treatment group will consume the diet once a month for 12 months. Metabolic, inflammatory, and regenerative pathways will be monitored to see the diet’s effect on mild cognitive impairment and early Alzheimer’s disease.^434^

**Table 9:** Highlighted Caloric Restriction Antiaging Clinical Trials

indication	intervention	status	sponsor	NCT no.
Aging	Caloric restriction	Complete	Duke University, USA	NCT00427193
Obesity/diabetes/hypertension/hyperlipidemia	Exercise|reduced calorie diet	Complete	University of Alabama at Birmingham, USA	NCT00955903
Aging	Calorie mimetic supplement|fasting mimicking diet	Active, not recruiting	TruDiagnostic, USA	NCT04962464
Alzheimer’s disease	Mediterranean diet|study supplement|low-fat diet	Active, not recruiting	University of Kansas Medical Center, USA	NCT03841539
Cognitive impairment|early Alzheimer’s disease	Fasting-mimicking diet ProlonADTM	Recruiting	University of Genova, Italy	NCT05480358

Research on **physical exercise** as an intervention for aging indications is well established, and clinical trials are seeing promising results. The Central Arkansas Veterans Healthcare System has completed a clinical trial on the use of Wii-Fit exercises to improve unsteady gait and postural balance in 30 veterans aged 65 and over. The intervention was performed for 45 min, 3 days a week for 8 weeks.^435^ Outcome measurements of gait and balance improved significantly in the intervention group, showing that the Wii-Fit exercise program was effective.^436^ Another study from the University of Kansas Medical Center recently published results for its clinical trial (NCT04009629) researching moderately intensive aerobic exercise and its effects on brain blood flow and biological factors after exercise (15 min) in participants with a genetic risk factor for developing Alzheimer’s disease. 61 participants aged 65–80 years old with the apolipoprotein e4 (APOE4) gene were enrolled.^437^ The results revealed increases in cerebral blood flow and neurotrophic response to acute aerobic exercise for all participants regardless of APOE4 status.^438^ The long-term goal of the study team with this acquired knowledge is to create a personalized exercise prescription for the treatment of Alzheimer’s disease.

Upcoming clinical trials are also continuing to examine physical exercise and its effects on the cognitive function in the aging population with heart failure (Table 10). The Montreal Heart Institute is recruiting 218 participants aged 60 years and older to research physical exercise and cognitive training interventions on cognition and brain health in patients with heart failure for clinical trial NCT04970888. Cognitive training sessions will be 30 min and physical exercise sessions will be 60 min, three times a week for 6 months.^439^ This combined intervention method has not been widely studied, so results should be of particular interest. Another indication of post-traumatic stress disorder (PTSD) among elderly veterans is also currently in the clinical development pipeline (NCT04199182). The VA Office of Research and Development is recruiting to investigate this quickly emerging field of study. A three times a week supervised exercise program will continue for 6 months to see its effects on PTSD symptoms and related conditions such as sleep disorders among 188 older veterans.^440^

**Table 10:** Highlighted Physical Exercise Antiaging Clinical Trials

indication	intervention	status	sponsor	NCT no.
Alzheimer’s disease	Moderate intensity aerobic exercise	Complete	University of Kansas Medical Center, USA	NCT04009629
Unsteady gait|postural balance	Wii-fit exercises	Complete	Central Arkansas Veterans Healthcare System, USA	NCT02190045
Cognitive function	Cognitive training| exercise training	Recruiting	Montreal Heart Institute, Canada	NCT04970888
Post-traumatic stress disorder	Exercise training	Recruiting	VA Office of Research and Development, USA	NCT04199182

**Stem cell transplantation** has shown promising results in clinical trials for aging-related conditions (Table 11). Longeveron studied the use of allogeneic mesenchymal stem cells (allo-MSCs) for the condition of frailty in a successful clinical trial (NCT02065245). Thirty patients with a mean age of 75.5 years received either a 100-million or 200-million cell dose infusion. Significant reduction of inflammatory marker TNF-α and early and late-stage T-cells activation occurred. B cell intracellular TNF-α and physical performance among participants was also improved in both treatment groups.^441^ Longeveron also explored the use of MSCs through its biotherapeutic candidate Lomecel-B for the treatment of Alzheimer’s disease (AD) in phase I clinical trial NCT02600130. Thirty participants were enrolled with low and high dose infusion groups of 30 and 100 million cells. Significant improvement was seen for inflammatory and AD biomarkers along with neurocognitive assessments.^442^ Due to these encouraging results, Alzheimer’s disease treatment with Lomecel-B is further researched in phase II trial NCT05233774 currently recruiting participants.

**Table 11:** Highlighted Stem Cell Therapy Antiaging Clinical Trials

indication	intervention	status	sponsor	NCT no.
Frailty	Allogeneic mesenchymal stem cells	Complete	Longeveron, USA	NCT02065245
Alzheimer’s disease	Lomecel-B (allogeneic mesenchymal stem cells)	Complete	Longeveron, USA	NCT02600130
Male sexual dysfunction	Umbilical cord mesenchymal stem cell	Recruiting	Vinmec Research Institute of Stem Cell and Gene Technology, Vietnam	NCT05345418
Diminished ovarian response	Amniotic mesenchymal stem cells	Not yet recruiting	The First Affiliated Hospital with Nanjing Medical University, China	NCT04706312

The Vinmec Research Institute of Stem Cell and Gene Technology are exploring the use of MSC for male sexual dysfunction in a phase I/II clinical trial (NCT05345418). They are currently recruiting male subjects aged 50–70 years old with sexual functional deficiency. Treatment groups will receive two iv doses of 1.5 million cells/kg body weight spaced out by 3 months. Various biomarkers, testosterone levels, and sexual life quality information will be measured. The First Affiliated Hospital with Nanjing Medical University has an upcoming phase I clinical trial (NCT04706312) researching the use of amniotic mesenchymal stem cells (AMSCs) for the treatment of infertility is people with diminished ovarian response. Subjects will receive an iv injection of AMSCs and measurements recorded for ovarian function and *in vitro* fertilization such as stimulated follicles, number of oocyte retrieval, fertilization rate, etc.^443^

**Dietary supplementation** is an antiaging strategy that targets a wide range of indications (Table 12). The University of Sherbrooke, Canada, researched the use of medium chain triglycerides (MCT) combined with aerobic (AE) exercise on ketone production in a group of 20 women (prediabetic and healthy) over the age of 60 years through clinical trial NCT02678390. They discovered that MCT (30 g/day for 5 days) combined with AE (30 min) was more ketogenic in older women than MCT or AE alone.^444^ A clinical trial (NCT02446314) researching the use of two different blueberry formulations for the treatment of cognitive decline in 125 participants 65–80 years old. Treatment groups consisted of a 6-month daily regime of 450–900 mg of blueberry powder or 100 mg of blueberry extract. The results reveal that the blueberry extract intervention can improve episodic memory and cardiovascular biomarkers over 6 months. The same effects were not observed for the blueberry powder intervention or with the measurements of executive function, working memory, or mood.^445^

**Table 12:** Highlighted Dietary Supplementation Antiaging Clinical Trials

indication	intervention	status	sponsor	NCT no.
Prediabetes	MCT|aerobic exercise	Complete	University de Sherbrooke, Canada	NCT02678390
Cognitive decline	Wild blueberry powder or extract	Complete	University of Reading, U.K.	NCT02446314
Osteoarthritis, knee	Veg, bovine, fish, or chicken collagen peptide	Active, not recruiting	NovoBliss Research, India	NCT05613660
Skin laxity	WonderLab collagen tripeptide drink	Not yet recruiting	Shenzhen Precision Health Food, China	NCT05682092
Telomere shortening/vascular diseases	TA-65	Not yet recruiting	Medical College of Wisconsin, USA	NCT05598359

Several current studies are exploring the use of collagen for age-related indications. NovoBliss Research, India, is researching the effect of vegetable, bovine, fish, and chicken collagen peptide for indications such as skin elasticity, wrinkles, and hydration, hair thickness and density, along with joint pain and osteoarthritis. 125 participants are currently enrolled with treatment groups of 0.5–10 g/day.^446^ Shenzhen Precision Health Food, China, also has a registered trial (NCT05682092) researching the use of collagen peptide to increase skin moisture and elasticity. The trial is not yet recruiting but plans to enroll 70 middle aged (30–50 years old) women who will consume 25 mL twice a day for two months if in the treatment group. Lastly, we discuss Medical College of Wisconsin’s clinical trial NCT05598359 that is not yet recruiting but will be researching the use of TA-65, a purified small molecule extracted from Astragalus root. 180 participants will be recruited to investigate the use of TA-64 (250 U) taken once per day on microvascular function and blood pressure.^447^

## 7 Outlook and Perspectives

Aging is generally defined as the accumulation of detrimental changes taking place in cells and tissues with advancing age, which bring about the increased risk of disease and death. The emerging standpoint defines aging as a particularly complex, multifactorial process. Antiaging research aims to identify strategies to promote healthy aging and extend lifespan. The major perspectives in the antiaging exploration generally fall into two groups: (i) lifestyle modifications and (ii) pharmacological/genetic manipulations. More specifically, the foremost approaches in antiaging research include the following:

- Extensive current research explores the genetic basis of aging and age-related diseases and investigates the potential of **genetic interventions**, such as gene therapy, to prevent or reverse age-related damage.
- **Lifestyle interventions**, such as caloric restriction, exercise, and stress reduction, have been believed to promote healthy aging and extend lifespan. Widespread research is currently exploring the mechanisms underlying these effects and developing strategies to promote healthy behaviors.
- **Pharmaceutical interventions** explore the potential of drugs that target age-related pathways or senescent cells, to prevent or delay age-related diseases.
- **Regenerative medicine** aims to restore or replace damaged tissues and organs and has the potential to promote healthy aging and extend lifespan.
- **Social and environmental factors**, such as social support, access to healthcare, and exposure to toxins, can influence the aging process. The effects of these factors are being explored, and interventions to promote healthy aging are being currently developed.
- **Artificial intelligence** (AI) is being used to analyze large amounts of data and identify patterns that could be used to predict or prevent age-related diseases. AI is also being used to develop personalized antiaging interventions based on an individual’s genetic and lifestyle factors.

More specifically, with particular attention to **brain health** maintenance, the following antiaging lifestyle strategies can help prevent or slow down age-associated brain function decline:

- **Physical exercise** has been shown to improve brain function, increase brain volume, and reduce the risk of cognitive decline.
- **Mental stimulation:** Engaging in mentally stimulating activities can help maintain cognitive function and reduce the risk of age-related cognitive decline.
- Eating a **healthy diet** rich in fruits, vegetables, whole grains, and lean protein can help reduce inflammation and oxidative stress in the brain.
- **Stress reduction:** Chronic stress has been linked to accelerated brain aging, so finding ways to manage stress like practicing mindfulness and meditation can be beneficial.
- Staying **socially active** and connected can help maintain cognitive function and reduce the risk of cognitive decline.
- Getting adequate **sleep** is essential for brain health and has been linked to improved cognitive function and a reduced risk of cognitive decline.

All these strategies are not mutually exclusive, and antiaging research often involves a multidisciplinary approach that combines different approaches to promote healthy aging and extend lifespan. Yet, regardless of the extensive research for antiaging therapeutics, based on the general understanding that aging is malleable in diverse species, to date, no convincing evidence has been provided indicating that the administration of existing antiaging remedies can markedly slow aging or increase longevity in humans. The major roadblocks that antiaging research and development is currently facing are summarized in Table 13.

**Table 13:** Major Roadblocks in the Antiaging Research and Development

roadblocks	details
Complexity of aging	Aging is a complex process that involves multiple mechanisms and pathways, and it is difficult to identify specific targets for intervention.
Insufficiency of knowledge	Despite advances in antiaging research, there is still much to be learned about the underlying mechanisms of aging and how they contribute to age-related diseases.
Heterogeneity of aging	Aging is a heterogeneous process, and there is significant individual variability in how people age. This makes it challenging to develop personalized antiaging interventions that are effective for everyone.
Regulatory challenges	Developing and testing antiaging interventions can be challenging due to regulatory barriers and the need for long-term clinical trials to demonstrate safety and efficacy.
Cost	Developing antiaging interventions can be expensive, and there may be limited financial incentives for companies to invest in this area.
Ethical considerations	There are ethical considerations associated with antiaging interventions, such as concerns about equity and access, and the potential for unintended consequences.
Perception and stigma	There is still a stigma associated with aging and a perception that aging is an inevitable and irreversible process. This can make it challenging to attract funding and support for antiaging research and development.

Despite these multiple difficulties and complexities, antiaging research is a rapidly growing field, and researchers are working to overcome these challenges to develop effective interventions to promote healthy aging and extend lifespan. Certain important steps forward toward the understanding of the aging process have been made so that it is no more an incomprehensible issue. The extensive efforts and research activities in the antiaging strategies field has led to several important outcomes:

- Identification of **biomarkers of aging**. Researchers have identified biomarkers that can predict biological age and the risk of age-related diseases. These biomarkers can be used to develop personalized antiaging interventions and monitor the effectiveness of these interventions.
- Development of **interventions to promote healthy aging**. Antiaging research has led to the development of interventions, such as caloric restriction, exercise, and stress reduction, that can promote healthy aging and extend lifespan.
- Identification of potential **drug targets**. Researchers have identified several pathways and targets that could be targeted by drugs to prevent or delay age-related diseases.
- Development of **regenerative medicine therapies**. Antiaging research has led to the development of regenerative medicine therapies that can restore or replace damaged tissues and organs, which could have important implications for treating age-related diseases.
- **Extension of lifespan in animal models**. Antiaging interventions have been shown to extend lifespan in animal models, which provides proof-of-concept for the potential of these interventions to promote healthy aging in humans.
- Improved **understanding of the biology of aging**. Antiaging research has led to a better understanding of the biological mechanisms underlying aging and age-related diseases, which could lead to the development of new interventions and therapies.

The progress in antiaging research has shown the potential to improve health and quality of life for older adults by promoting healthy aging and delaying the onset of age-related diseases. In pursuing a solution to the aging issues, it is necessary to keep clear in mind that the goal of research on aging hallmarks and antiaging strategies is not to enhance human longevity but to enhance healthy, active longevity, free from disability and functional incapacity.^403,448^ Such understanding of aging has resulted in a shift in the approach for aging interventions from antiaging to healthy aging. Focusing on prevention may lead to new successes in achieving healthy aging.^449^

## Supporting Information Available

Table S1 listing antiaging drugs included in the CAS Content Collection (extended list); Table S2 listing ntural antiaging agents most widely represented in the CAS Content Collection including structures; Table S3 listing synthetic antiaging agents most widely represented in the CAS Content Collection including structures; Figure S1 showing yearly NIH funding for projects related to antiaging research (PDF)
