# Supplementary material for: Antiaging Strategies and Remedies: A Landscape of Research Progress and Promise
Source: ACS Chem Neurosci. 2024 Jan 12;15(3):408–46. doi: 10.1021/acschemneuro.3c00532 (PMC10853939; doi:10.1021/acschemneuro.3c00532)
Supplement: Supplementary file 1 — cn3c00532_si_001.pdf [file cn3c00532_si_001.pdf]

Anti-aging Strategies and Remedies:
A Landscape of Research Progress and Promise

Rumiana Tenchov, Janet M. Sasso, Xinmei Wang, Qiongqiong Angela Zhou*
CAS, a division of the American Chemical Society

Table S1. Representative anti-aging drugs in the CAS Content Collection

Anti-aging compounds	CAS Reg #	Number of journal articles	Number of patents	ALL
Acacetin	480-44-4	21	12	33
Acarbose	56180-94-0	87	19	106
Acetyl-coenzyme A (Acetyl	72-89-9	147	11	158
Acetyl-L-carnitine	3040-38-8	722	596	1318
Acteoside	61276-17-3	28	16	44
Adapalene	106685-40-9	7	52	59
Agmatine	306-60-5	10	4	14
Allantoin	3040-38-8	45	652	697
Allicin	539-86-6	23	46	69
Aloesin	30861-27-9	2	16	18
Anacardic acid	11034-77-8	3	8	11
Andrographolide	5508-58-7	17	15	32
Antcin M	1005344-44-4	1	2	3
Apigenin	520-36-5	132	102	234
Arginine, L-	74-79-3	726	615	1341
Aspirin	50-78-2	3569	162	3731
Astaxanthin	472-61-7	136	436	572
Astragaloside	17429-69-5	27	31	58
Azacitidine	320-67-2	47	21	68
AZD8055	1009298-09-2	3	4	7
Bacoside A	11028-00-5	3	0	3
Baicalein	491-67-8	41	37	78
Berberine	2086-83-1	80	34	114
Betaine	107-43-7	113	571	684
Betulinic acid	472-15-1	30	49	79
Butein	487-52-5	10	19	29
Caffeic acid	331-39-5	226	129	355
Carnosic acid	3650-09-7	22	36	58
Carnosine, L-	305-84-0	177	379	556
β-Carotene	7235-40-7	687	327	1014
Catalpol	2415-24-9	20	6	26
Catechin	154-23-4	512	288	800
Celastrol	34157-83-0	13	7	20
Chicoric acid	6537-80-0	8	11	19
Chlorogenic acid	327-97-9	221	122	343
Chromium picolinate	14639-25-9	10	18	28
Chrysin	480-40-0	32	39	71

Chrysophanol	481-74-3	8	8	16
Coenzyme Q10	303-98-0	242	541	783
Crocin	42553-65-1	228	64	292
Curculigoside	85643-19-2	5	1	6
Curcumin	458-37-7	444	210	654
Cycloastragenol (Telomera	78574-94-4	35	18	53
Danazol	17230-88-5	15	5	20
Dapagliflozin (Dapagliflozo	461432-26-8	45	9	54
Dasatinib	302962-49-8	39	21	60
Dehydroabietic acid	1740-19-8	6	7	13
Dehydroepiandrosterone (	53-43-0	388	132	520
2-Deoxy-D-glucose	154-17-6	83	14	97
Elamipretide	736992-21-5	4	7	11
Ellagic acid	476-66-4	115	156	271
Emodin	518-82-1	39	18	57
Entinostat (SNDX-275; MS-	209783-80-2	3	11	14
(-)-Epicatechin	490-46-0	202	119	321
Epigallocatechin-3-gallate	989-51-5	292	180	472
Epitalon	307297-39-8	31	1	32
Ergoloid mesylate (Hyderg	8067-24-1	6	2	8
17-α-Estradiol	57-91-0	24	3	27
Ethanolamine	141-43-5	40	132	172
Ferulic acid	1135-24-6	184	248	432
Fisetin	528-48-3	59	47	106
Fucoxanthin	3351-86-8	23	27	50
Galangin	548-83-4	16	11	27
Gallic acid	149-91-7	345	144	489
Garcinol	78824-30-3	4	1	5
Garlicin	2179-57-9	10	19	29
Gastrodin	62499-27-8	16	8	24
Genistein	446-72-0	216	150	366
Gingerol	58253-27-3	14	15	29
[6]-Gingerol	23513-14-6	9	9	18
Ginsenoside Rg1	22427-39-0	98	30	128
Glabridin	59870-68-7	9	85	94
Glaucarubinone	1259-86-5	2	1	3
Glipizide	29094-61-9	14	16	30
β-Guanidinopropionic acid	353-09-3	4	0	4
Glucosamine, D-	3416-24-8	121	190	311
HDTIC-1	1229519-12-3	2	0	2
HDTIC-2	1229519-13-4	2	0	2
Hesperidin	520-26-3	72	99	171
Honokiol	35354-74-6	19	31	50
Human growth hormone (I	12629-01-5	200	18	218
Huperzine A	102518-79-6	10	6	16
Hyaluronic acid	9004-61-9	584	2081	2665

Hydroxycitric acid (HCA)	27750-10-3	8	10	18
4-Hydroxy phenyl N-tert-b	223649-80-7	2	2	4
Icariin	489-32-7	38	14	52
Idebenone	58186-27-9	15	49	64
Isotretinoin	4759-48-2	60	61	121
Kaempferol	520-18-3	204	111	315
α-Ketoglutarate	328-50-7	81	31	112
Lactic acid	50-21-5	414	555	969
Lauric acid	143-07-7	92	236	328
α-Lipoic acid	1200-22-2	209	346	555
Liquiritin	551-15-5	11	17	28
Lithium	7439-93-2	266	145	411
Lutein	127-40-2	385	189	574
Luteolin	491-70-3	146	100	246
Mangiferin	4773-96-0	16	15	31
Meclofenoxate (Centrophe	51-68-3	34	9	43
Melatonin	73-31-4	637	176	813
Metformin	657-24-9	936	105	1041
Methylthiomethane (Dime	75-18-3	4	10	14
Mogroside	872869-50-6	3	9	12
Monascin	21516-68-7	4	0	4
Myricetin	529-44-2	87	60	147
N-Acetylcysteine	7218-04-4	48	9	57
Naringenin	480-41-1	88	63	151
Nicotinamide	98-92-0	575	884	1459
Nicotinamide riboside	1341-23-7	62	41	103
Nordihydroguaiaretic acid	500-38-9	28	46	74
Oleanolic acid	508-02-1	77	69	146
Oleuropein	32619-42-4	60	40	100
Oligonol	851983-55-6	6	3	9
Orientin	28608-75-5	18	10	28
Oxymatrine	16837-52-8	4	3	7
Panobinostat	404950-80-7	5	9	14
Pegvisomant	218620-50-9	3	4	7
Phosphatidylserine	-	208	84	292
Phycocyanin C	11016-15-2	24	23	47
Piracetam	7491-74-9	43	10	53
Polydatin (Piceid)	27208-80-6	26	29	55
Procaine (Gerovital H3, GH	59-46-1	60	35	95
Procyanidin B3	3567-23-9	1	2	3
Protandim	877143-83-4	3	0	3
Protocatechuic aldehyde	139-85-5	15	25	40
Pterostilbene	537-42-8	47	40	87
Puerarin	3681-99-0	32	25	57
Pyridopyrimidines	-	1	0	1
Quercetin	117-39-5	654	303	957
Quercetin caprylate	87798-94-5	3	2	5

Rapamycin (Sirolimus)	53123-88-9	1411	99	1510
Resveratrol	501-36-0	1270	602	1872
Rosiglitazone	122320-73-4	57	30	87
Rosmarinic acid	20283-92-5	83	69	152
Rutin	153-18-4	245	183	428
Ruxolitinib	941678-49-5	23	8	31
Salicylic acid	69-72-7	201	711	912
Salsalate	552-94-3	4	13	17
Sappanone A	112458-02-3	1	1	2
Selegiline (L-deprenyl, Elde	14611-51-9	109	28	137
Seletinoid G	637357-50-7	4	2	6
Sesamin	1143-70-0	27	17	44
Silymarin	65666-07-1	43	55	98
Sirtuin	438496-81-2	2235	180	2415
Sodium butyrate	156-54-7	37	17	54
Sodium phenylbutyrate (B	1716-12-7	7	8	15
Spermidine	124-20-9	235	52	287
Sulforaphane	4478-93-7	73	27	100
Tadalafil	171596-29-5	7	11	18
Tambulin	571-72-2	2	0	2
Tazarotene	118292-40-3	7	34	41
Tectorigenin	548-77-6	3	6	9
Tetrahydrocurcumin	36062-04-1	24	37	61
Theaflavin	4670-05-7	26	14	40
Theanine	3081-61-6	32	52	84
Tiliroside	20316-62-5	7	5	12
Trametinib	871700-17-3	7	4	11
Tretinoin	302-79-4	229	319	548
Trichostatin A	58880-19-6	28	4	32
Tyrosol	501-94-0	45	16	61
Urolithin A	1143-70-0	37	12	49
Ursolic acid	77-52-1	69	91	160
Valproic acid	99-66-1	148	23	171
Vinpocetine	42971-09-5	10	8	18
Visomitin	934826-68-3	13	3	16
Vitamin A (Rretinol)	68-26-8	1141	934	2075
Vitamin C (L-ascorbic acid)	50-81-7	2030	2482	4512
Vitamin E	1406-18-4	1593	1879	3472
Vitexin	3681-93-4	29	16	45
Vorinostat	149647-78-9	22	21	43

Anti-aging Strategies and Remedies: A Landscape of Research Progress and Promise

Rumiana Tenchov, Janet M. Sasso, Xinmei Wang, Qiongqiong Angela Zhou*

CAS, a division of the American Chemical Society

Table S2. Natural anti-aging agents most widely represented in the CAS Content Collection including structures [1-18]

Natural anti-aging compounds	CAS REG #	Sources	Mechanism of action / Anti-aging strategy	Application / Benefits	Structure	Number of journal articles	Number of patents
Acetyl-coenzyme A (Acetyl CoA)	72-89-9	Oily fish (salmon, tuna), organ meat (liver), grains	Inhibits oxidation of proteins, lipids, and DNA	heart conditions, muscular dystrophy	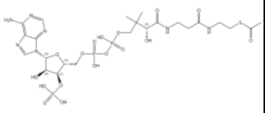	147	11
Acetyl-L-carnitine	3040-38-8	Red meat, poultry, fish, dairy foods	Decline in interfibrillar mitochondria carnitine palmitoyltransferase 1 activity	Improves cognitive and neurological function, reduces mental fatigue, improve mood, alertness	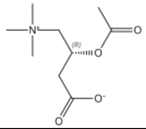	722	596
Allantoin	97-59-6	Chamomile, wheat sprouts, sugar beet, comfrey	Helps in shedding of dead skin cells & cell turnover	Skin moisturizing and soothing, exfoliation, wound healing	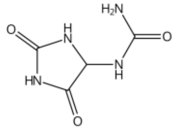	45	652
Apigenin	520-36-5	Chamomile, celeriac, parsley	Inhibits skin inflammation by down-regulating transcription factors including AP-1, NF-κB, STAT; CD38 inhibitor	Antidiabetic, chemoprevention	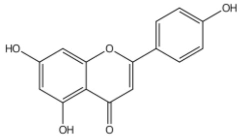	132	102
Arginine, L-	74-79-3	Meat, fish, nuts & seeds, legumes, whole grains, dairy	Reduces oxidative stress and inflammation resulting in decreased NF-κB level and activity; vascular smooth muscle cell relaxation	Reduces systolic and diastolic blood pressure in hypertensive patients	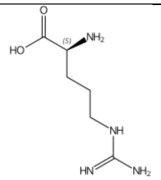	726	615

Astaxanthin	472-61-7	Phaffia Rhodozyma	Protects cell membranes against reactive oxygen and nitrogen species and oxidative damage	Antioxidant, anti-inflammatory	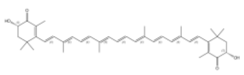	136	436
Berberine	2086-83-1	Coptidis rhizome, Barberry plants, Chinese goldthread	AMPK activator, enhancing UCP2 expression, inhibits oxidative stress	Agent against dyslipidemia, antidiabetic, antioxidant, antiobesity, antiangiogenic	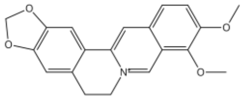	80	34
Betaine	107-43-7	Sugar beets	Lowers levels of homocysteine in circulating blood	Osmoregulator, ergogenic, supporting heart health	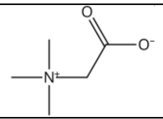	113	571
Caffeic acid	331-39-5	Coffee, red wine, berries, apples, olives, artichokes, pears	Regulates proteostasis; alleviates neuroinflammation and neurodegeneration	Antioxidant; liver damage prevention, cognitive function improvement; psychoactive drug	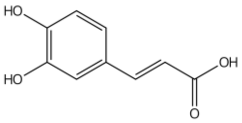	226	129
L-Carnosine	305-84-0	Meats: turkey, chicken, beef, pork	Reacts with methyl-glyoxal and scavenges ROS thus protective toward aging and ischemia; increase verbal episodic memory; increase of cerebral blood flow	Anti-aging, antioxidant	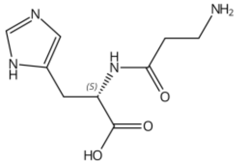	177	379
β-Carotene	7235-40-7	Fungi, plants, fruits	Photoprotecting agent, reduces rate of mitochondrial mutation	Antioxidant agent, promotes healthy skin, supports immune system, eye health and vision	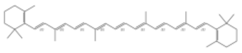	687	327
Catechin	154-23-4	Tea polyphenols	Rich in oligomeric proantho-cyanidins, thus prevents premature aging	Anti-inflammatory agent	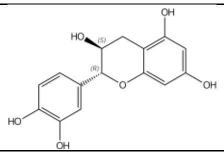	512	288
Chlorogenic acid	327-97-9	Coffee, tea, bamboo	Glucose regulation; induce continuous phosphorylation of ERK1/2	Antioxidant, neuroprotective, anti-inflammatory, gastro-protective, antirheumatic, antihypertensive, anti-atherothrombotic	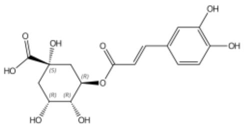	221	122

Coenzyme Q10	303-98-0	Meat, fish, nuts	Increases production of key antioxidants such as superoxide dismutase; reduces levels of lipid peroxidation; intensify blood flow, protect blood vessels by upholding nitric oxide	Anti-inflammatory, anti-atherogenic	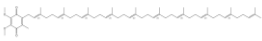	242	541
Crocin	42553-65-1	Flowers of crocus and gardenia, saffron	Reduces oxidative stress and ROS through enhancement of gene expression of Nrf2, HO-1, and antioxidant enzymes CAT, GSH, and SOD; counteract oxidative stress, mitochondrial dysfunction and neuroinflammation	Degenerative disease, metabolic syndrome; antioxidant, neuroprotective agent	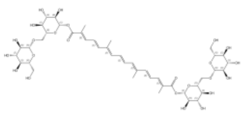	228	64
Curcumin	458-37-7	Curcuma longa, turmeric	Inhibits TOR pathway; autophagy inducing	Anti-inflammatory, antioxidant	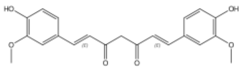	444	210
Dehydroepiandro-sterone (DHEA)	53-43-0	Wild yam, soy	Activates PPARα and constitutive androstane receptor (CAR)	Raises androgen and estrogen levels; improves bone & cardiovascular health, insulin sensitivity, and mood	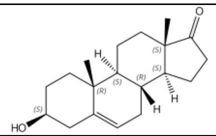	388	132
Ellagic acid	476-66-4	Fruits: grapes, strawberries, pomegranate	Decreases amount of inflammatory cytokines, regulates the activities of antioxidant enzymes	Anti-inflammatory, antioxidant; treats viral and bacterial infections	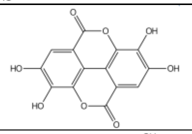	115	156
(-)-Epicatechin	490-46-0	Cocoa	Stimulates mitochondrial respiration and biogenesis	Enhances nitric oxide production for improved vascularity, circulation, and resilience	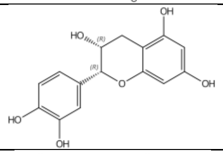	202	119
Epigallocatechin-3-gallate (EGCG)	989-51-5	Green tea	Regulates cytokine secretion, autophagy inducing	Antioxidant, anti-inflammatory	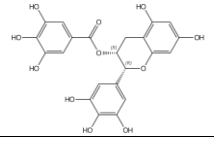	292	180
Ethanolamine	141-43-5	Daikon radish, caraway, muscadine grape, lemon grass	Increases amount of cellular phosphatidylethanolamine, thus stimulating cytoprotective autophagy and anti-aging protection	Anti-aging, inducing autophagy	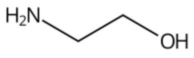	40	132

Ferulic acid	1135-24-6	Beet roots, oranges, carrots	Restrains radiation-induced oxidative stress by ceasing free radical chain reaction	Anti-aging, anticancer	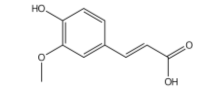	184	248
Fisetin	528-48-3	Nuts, strawberries, apples, mangoes, persimmons	Inhibits NF-κB activation, promotes Nrf2 activity to prevent neurodegeneration; lipoxygenase inhibitor	Neuroprotective agent; anti-aging, anticancer, anti-inflammatory	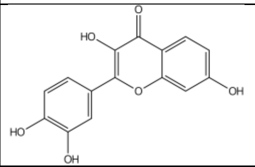	59	47
Gallic acid	149-91-7	Rose flower	Anti-inflammatory mechanisms involve MAPK and NF-κB signaling pathways; reducing release of inflammatory cytokines, chemokines	Improves cognitive function, motor function; anti-inflammatory	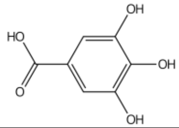	345	144
Genistein	446-72-0	Soy products	Enhancing skin collagen by stimulating subcutaneous VEGF expression and increasing TGF-β in skin	Maintains arterial elasticity, blood glucose control, prevents hypertension, prostate & breast cancer	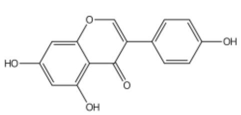	216	150
Ginsenoside Rg1	22427-39-0	Ginseng	Improves cognitive function; stimulates glucose uptake, relieves oxidative stress; possible neuroprotective role	Suppressive effects in neurodegenerative conditions; neuroprotectant, anti-inflammatory	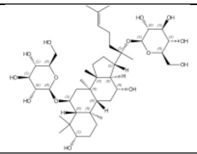	98	30
D-Glucosamine	3416-24-8	Chitin from hard outer shells of shrimp, lobster, crab	Prevents collagen degeneration in chondrocytes; slow cartilage deterioration in the joints	Cartilage-protecting, anti-inflammatory	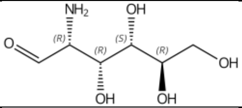	121	190
Hesperidin	520-26-3	Citrus genus	Inhibition of signaling pathway related to MMP-9 activated by UVB radiation	Antioxidant, anti-inflammatory, anti-aging	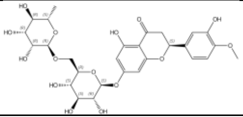	72	99
Hyaluronic acid	9004-61-9	Rooster combs (the red part on a rooster's neck)	Major component of extracellular matrix, key role in tissue regeneration, inflammation, angiogenesis, wound repair	Reduces facial skin wrinkles	N/A	584	2081
Kaempferol	520-18-3	Spinach, kale, tarragon	Prevents the activation of p38 mitogen-activated protein kinase C-JNK	Antiapoptotic, antiangiogenic	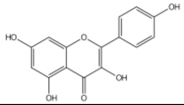	204	111

α-Ketoglutarate	328-50-7	Intermediate of the tricarboxylic acid cycle	Nitrogen scavenger, glutamate and glutamine source, promotes protein synthesis, prevents protein degradation in muscles	Improves amino acid metabolism	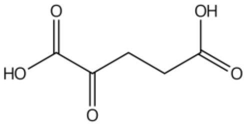	81	31
Lactic acid	50-21-5	Pickled vegetables, yogurt	Stimulates collagen renewal	Skin moisturizing and antiwrinkle properties	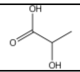	414	555
Lauric acid	143-07-7	Papaya	Rich in saturated fatty acids, hydrates skin	Anti-aging, antimicrobial, anti-bronchitis; soothes inflamed skin, inhibits acne bacteria	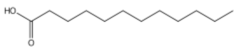	92	236
α-Lipoic acid	1200-22-2	Spinach, broccoli, potatoes, yeast, tomatoes, carrots	Suppression of p38 and p53 at gene level	Antioxidant, anti-aging	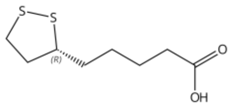	209	346
Lithium	7439-93-2	Ore mining, salt water from underground lakes	Modulates the release of dopamine or serotonin in brain	Mood stabilizer, helps to treat bipolar episodes	Li	266	145
Lutein	127-40-2	Green vegetables	Preserves visual function by preventing degradation of rhodopsin and synaptophysin	Prevents skin aging, age-related macular degeneration; antioxidant	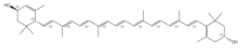	385	189
Luteolin	491-70-3	Vegetables, tea	Reduces neuroinflammation and improves learning and memory; inhibits vascular inflammation	Anti-inflammatory, antioxidant	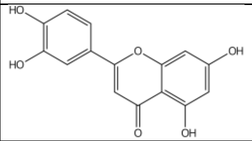	146	100
Melatonin	73-31-4	Tart cherries, tomatoes, corn, asparagus, olives, pomegranate, nuts, sunflower, mustard, flax seeds	Restores mitochondrial membrane permeability, promotes antioxidant enzymes including glutathione peroxidase, superoxide dismutase, glutathione reductase, catalase	Antioxidant, anti-inflammatory, autophagy inducing, anti-aging	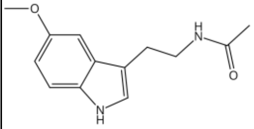	637	176
Myricetin	529-44-2	Berries, red wine	Reduces epidermal thickening provoked by UVB and suppress MMP-9 protein expression and enzyme activity	Anti-aging; inhibits hyper-glycemia, decreases hepatic triglyceride, reduces oxidative stress and cholesterol contents, protects liver injury	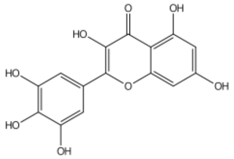	87	60

Naringenin	480-41-1	Grapefruit, bergamot, orange, cherries, tomatoes, cocoa, oregano, mint	Enhances antioxidant ability by activating Nrf2 causing HO-1 expression; inhibit NF-κB activation in macrophages	Anti-inflammatory, antioxidant	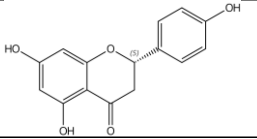	88	63
Nicotinamide	98-92-0	Meat, milk, eggs, green vegetables	DAC activator of SIRT1; manages the NF-κB-mediated transcription and inhibits mast cells degranulation; calorie mimetic	Cell proliferation and improvement in skin texture	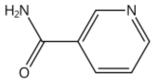	575	884
Nicotinamide riboside	1341-23-7	Cow's milk	Improves glucose tolerance, reduces age-related weight gain, exhibits neuroprotective effects	Anti-aging; neuroprotection and vascular protection	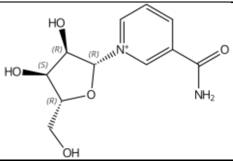	62	41
Oleanolic acid	508-02-1	Olea europaea, Viscum album, Aralia chinensis	Regulates macrophage polarization in adipose tissue	Antioxidant, anti-inflammatory, antiviral, anti-obesity, antidiabetic	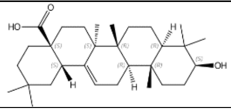	77	69
Oleuropein	32619-42-4	Olives	Avoids the reduction of proteasome activities upon senescence	Antiatherogenic, antioxidant, anticancer	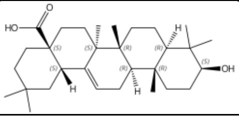	60	40
Phosphatidylserine	1446756-47-3	Soybeans, egg yolks, liver	Restores acetylcholine release, increases endogenous choline for de novo acetylcholine synthesis	Antioxidant; reduces stress, anxiety, and depression	N/A	208	84
Quercetin	117-39-5	Apples, honey, raspberries, onion, red grape, cherries, citrus, green leafy vegetables	Prevents the production of tumor necrosis factor α in macrophages and IL-8 in lung A549 cells, as induced by lipopolysaccharides	Calorie mimetics, anti-inflammatory, anti-atherogenic	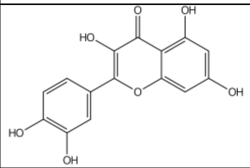	654	303
Rapamycin (Sirolimus)	53123-88-9	Soil bacterium: Streptomyces hygroscopicus	mTORC1 inhibitor; autophagy inducing, dietary restriction, calorie mimetic	Natural anti-fungal antibiotic	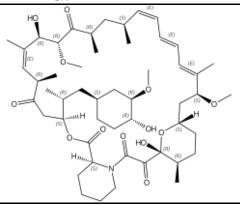	1411	99
Resveratrol	501-36-0	Grapes, berries, Polygonum cuspidatum	Sirtuin activator; calorie mimetics; telomerase activation	Neuroprotective, cardio-protective; antioxidant, anti-inflammatory, antidiabetic	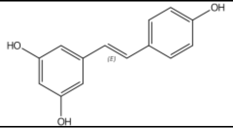	1270	602

Rosmarinic acid	20283-92-5	Rosemary	Prevents production of ROS and activation of abnormal mPTP provoked by high glucose, cytC release and caspase-3	Antibacterial, antiviral, antioxidant; anti-spasmodic, choleretic, hepatoprotective, antitumorigenic	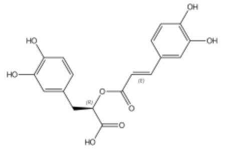	83	69
Rutin	153-18-4	Buckwheat, orange, black tea	HAT inhibitor, p300 inhibitor, PCAF inhibitor, NFκB inhibitor; chelate metal ions such as iron, inhibits Fenton's reaction	Strong antioxidant, antiangiogenic	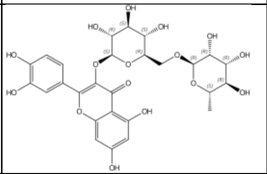	245	183
Salicylic acid	69-72-7	Broccoli, spinach, cauliflower, cucumber, mushroom	Decreases production of inflammatory prostaglandins	Anti-aging, anti-inflammatory	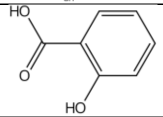	201	711
Sirtuin	438496-81-2	Kale, olives, red wine, strawberries	Maintains regular chromatin condensation; repair DNA damage; modulate oxidative stress; repress insulin resistance	Anti-aging, calorie mimetic; anti-inflammatory, stress resistance, fat & glucose metabolism, cardiac rhythm, mitochondria biogenesis	N/A	2235	180
Spermidine	124-20-9	Fresh green pepper, wheat germ, cauliflower, broccoli, mushrooms, cheeses	Autophagy inducing; reduces histone acetylation, lipid metabolism and regulates cell growth and signaling pathways	Antioxidant, anti-inflammatory	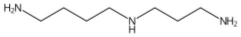	235	52
Sulforaphane	4478-93-7	Cruciferous vegetables: kale, bok choy, cabbage	Detoxifies carcinogens and pro-oxidants by blocking phase I metabolic enzymes; arrests cell cycle to impede cell proliferation; BACE1 inhibitor	Antioxidant, anti-inflammatory, protect DNA	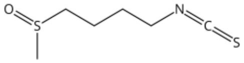	73	27
Tretinoin	302-79-4	Natural retinol	Stimulates mitotic activity, enhances loosely adherent corneocyte turnover	Anti-aging agent	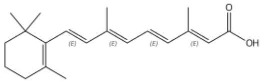	229	319
Ursolic acid	77-52-1	Apple peel, cranberry juice, grape skin, basil, rosemary, thyme, oregano, sage	Insulin secretagogue and insulinomimetic; regulates glucose uptake	Antihyperglycemic; insulin sensitivity increase; antioxidant, anti-inflammatory, antibacterial, antifungal	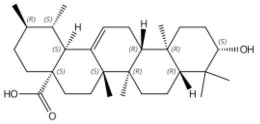	69	91

Vitamin A (Retinol)	68-26-8	Egg yolk, beef liver, carrots, pumpkin, sweet potatoes, mangos, papaya	Important to vision, growth, cell division, reproduction and immunity	Antioxidant	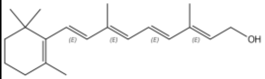	1141	934
Vitamin C (L-ascorbic acid)	50-81-7	Citrus, blackcurrant, rose hip, guava, chili pepper, parsley	Enhances collagen synthesis, slow down aging	Vital for immune system, heart, blood vessels; antioxidant, anti-hypertensive, immune stimulant; reduces heart disease and dementia risk	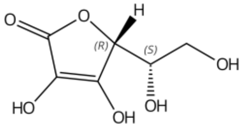	2030	2482
Vitamin E	1406-18-4	Nuts, seeds, vegetable oils: corn and soybean	Prevents peroxidation of unsaturated fatty acids; important to vision, reproduction, and blood, brain and skin health	Antioxidant	N/A	1593	1879

Table S3. Synthetic anti-aging agents most widely represented in the CAS Content Collection including structures [1-18]

Synthetic compounds	CAS Reg #	Mechanism of action / Anti-aging strategy	Application / Benefits	Structure	Number of journal articles	Number of patents
4-hydroxy phenyl N-tert-butylnitrone, CPI-1429	223649-80-7	Blocks signal transduction associated with neuroinflammation enhanced in neurodegenerative disorders	anti-aging, neuroprotectant	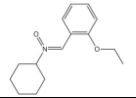	2	2
Acarbose	56180-94-0	Synthesized by soil bacteria Actinoplanes sp through its precursor valienamine	Inhibits alpha glucosidase; anti-diabetic for type 2 diabetes	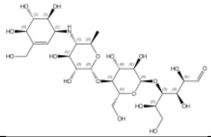	87	19
N-Acetylcysteine	7218-04-4	Increases cell protection to oxidative stress	Flu, dry eye, cough, and other lung conditions	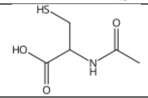	48	9
Adapalene	106685-40-9	Targets abnormal desquamation of skin and anti-inflammatory properties	Treatment of acne vulgaris	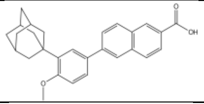	7	52
Aspirin	50-78-2	Inhibits the activity of cyclooxygenase (COX) which leads to formation of prostaglandins causing inflammation; regulating AMPK and insulin-like signaling pathway	Protects telomeres upon cell reproduction, restrains their shortening and related cell aging and death	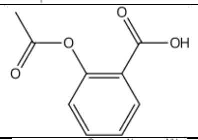	3569	162
Azacitidine	320-67-2	DNA methyltransferase inhibition by covalent bonding, resulting in DNA hypomethylation	Helps bone marrow grow normal blood cells; anti-atherogenic	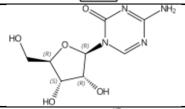	47	21
AZD8055	1009298-09-2	Blocks mTORC1 and mTORC2 signaling in AML	Antitumor effect used against neuroblastoma cells	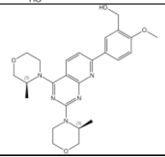	3	4
Danazol	17230-88-5	Androgen receptor agonist; ovarian steroidogenesis inhibitor; lowers gonadotropin levels in postmenopausal women	Treats endometriosis and fibrocystic breast disease by shrinking displaced tissue of uterus	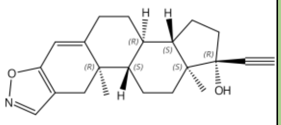	15	5
Dapagliflozin (Dapagliflozon, Farxiga)	461432-26-8	Inhibits SGLT2 thereby controlling hyperglycemic activity	Treatment of type 2 diabetes	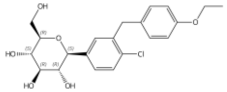	45	9

Dasatinib	302962-49-8	Inhibits proliferation, adhesion, migration and invasion of HCC cells by inhibiting Src tyrosine kinase and modifying SFK/FAK and PI3K/PTEN/Akt	Treatment of chronic myeloid leukemia	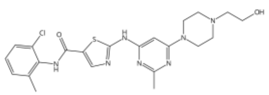	39	21
Elamipretide	736992-21-5	Targets mitochondrial inner membrane by its enrichment in cardiolipin	Anti-aging	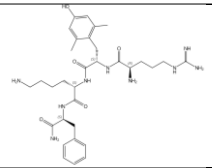	4	7
Entinostat (SNDX-275; MS-275)	209783-80-2	Class I and IV HDAC inhibitor (HDAC1, 2, 3)	Antitumor agent; histone deacetylase inhibitor; memory promoter	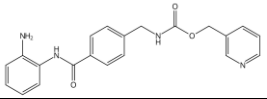	3	11
Epitalon	307297-39-8	Pineal gland, retina, brain function regulator, induces neuronal cell differentiation in stem cells	Anti-aging	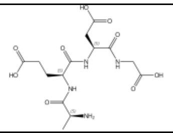	31	1
Ergoloid mesylate (Hydergine)	8067-24-1	Stimulates dopaminergic and serotonergic receptors and blocks alpha-adrenoreceptors	Treats dementia and age-related cognitive impairment; recovery after stroke	N/A	6	2
Human growth hormone (hGH)	12629-01-5	Regulates fat, muscle, tissue, and bone; stimulates the synthesis of chondroitin sulfate and collagen; promotes somatic growth	Maintains, builds, and repairs healthy tissue in brain and other organs; speeds up healing after injury and repair muscle tissue after exercise	N/A	200	18
Idebenone	58186-27-9	Blocks free radicals damage and sustains normal ATP levels	Alzheimer's disease, cognitive defects	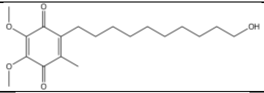	15	49
Isotretinoin	4759-48-2	Inhibits sebaceous gland function and keratinization	Acne, cutaneous conditions	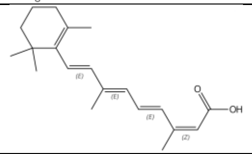	60	61
Meclofenoxate (Centrophenoxine, Lucidril)	51-68-3	Diminution of lipofuscin content of nerve cell; enhances activity of succinic and lactic dehydrogenase	Memory-boosting	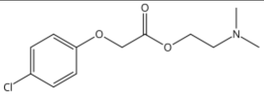	34	9
Metformin	657-24-9	Inhibits mTORC1 activity; growth hormone suppression	Type 2 diabetes, PCOS; dietary restriction, anti-atherogenic	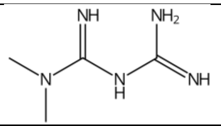	936	105

Panobinostat	404950-80-7	Inhibitor of class I HDAC; class IIa and IIb HDAC, class IV HDAC; histone deacetylase inhibitor	Antitumor agent	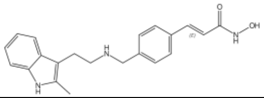	5	9
Piracetam	7491-74-9	Improves the function of neurotransmitter acetylcholine via muscarinic cholinergic receptors	Neuroprotective, anticonvulsant, improves neural plasticity in cognitive disorders and dementia	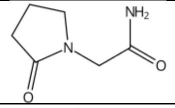	43	10
Procaine (Gerovital H3, GH3, KH-3)	59-46-1	Inhibits Na influx across voltage gated Na-channels in neuronal cell membrane of peripheral nerves	Anti-inflammatory, analgesic, vasodilatation, antioxidant; nervous system balance	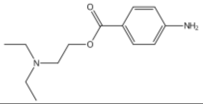	60	35
Pyridopyrimidines	-	High affinity DHFR inhibitor, thus decreasing tetrahydrofolate quantity required for pyrimidine and purine synthesis	KRAS inhibitors; anticancer	N/A	1	0
Rosiglitazone	122320-73-4	Inhibits PPAR-γ activity	Indicated for treatment of type 2 diabetes	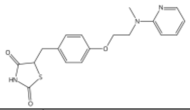	57	30
Ruxolitinib	941678-49-5	Inhibits JAK1 and JAK2, block dysregulated cell signaling, prevent abnormal blood cell proliferation	Treatment of myelofibrosis in adults	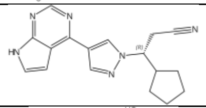	23	8
Salsalate	552-94-3	Decreases formation of prostaglandins involved in pain, fever, inflammation	Anti-inflammatory, antirheumatic	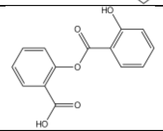	4	13
Selegiline (L-deprenyl, Eldepryl, Emsam)	14611-51-9	Selective inhibitor of MAO-B	Treats symptoms of Parkinson's disease and major depressive disorder	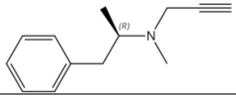	109	28
Seletinoid G	637357-50-7	Type I procollagen, tropoelastin, and fibrillin-1 expressions stimulation, reduces MMP-1	Anti-aging	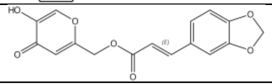	4	2
Sodium butyrate	156-54-7	Suppresses NFkB activation, inhibits interferon γ production and upregulation of PPARγ	Antioxidant, anti-inflammatory, autophagy inducing	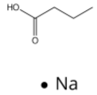	37	17
Sodium phenylbutyrate (Buphenyl)	1716-12-7	Inhibitor of class I HDAC, class IIa HDAC and class IIb HDAC	Antitumor agent; memory enhancement; amyloid burden reduction	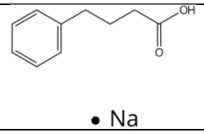	7	8

Tadalafil	171596-29-5	PDE 5 inhibitor with potent anti-aging activity	Treats erective dysfunction and benign prostatic hyperplasia	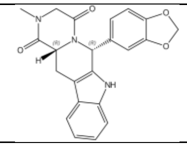	7	11
Tazarotene	118292-40-3	Binds to retinoic acceptor and modify gene expression	Used for psoriasis, acne, and psoriatic arthritis	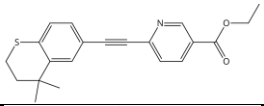	7	34
Trametinib	871700-17-3	ERK phosphorylation inhibitor, Ki67 suppression, tumor growth inhibition with mutant BRAF or RAS decreasing, G1 cell cycle arrest, apoptosis induction	Applied alone or in combination with dabrafenib to treat skin cancer (melanoma), thyroid cancer, and non-small cell lung cancer (NSCLC)	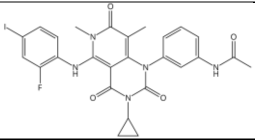	7	4
Trichostatin A	58880-19-6	Inhibitor of class I HDAC, class IIa HDAC, class IIb HDAC6	Antifungal, antibacterial, histone deacetylase inhibitor; protein synthesis inhibitor; antitumor, memory enhancement	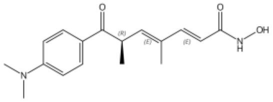	28	4
Valproic acid	99-66-1	Inhibitor of class I HDAC	Anticonvulsant, mood stabilizer; histone deacetylase inhibitor; GABA modulator; memory enhancement; CDK5 inactivation	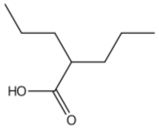	148	23
Vinpocetine	42971-09-5	Selective inhibitor of Ca(2+)-calmodulin dependent cGMP-PDE	Memory enhancer; against dementia, stroke, hearing loss	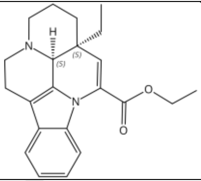	10	8
Visomitin	934826-68-3	Penetrates cellular membrane, accumulates in mitochondrial membrane inner leaflet where it is reduced or recharged	Treats inflammation associated with ophthalmic disease: dry eye, corneal wounds	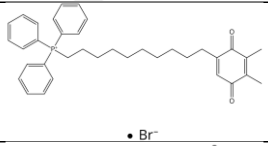	13	3
Vorinostat	149647-78-9	Inhibitor of class I HDAC, class IIb HDAC	Antitumor, memory enhancement	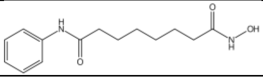	22	21

REFERENCES

1. Vaiserman, A. M., Lushchak, O. V.,Koliada, A. K., Anti-aging pharmacology: Promises and pitfalls. *Ageing Res Rev* **2016,** *31*, 9-35.
2. Shen, C. Y., Jiang, J. G., Yang, L., Wang, D. W.,Zhu, W., Anti-ageing active ingredients from herbs and nutraceuticals used in traditional Chinese medicine: pharmacological mechanisms and implications for drug discovery. *Br J Pharmacol* **2017,** *174*, 1395-1425.
3. Corrêa, R. C. G., Peralta, R. M., Haminiuk, C. W. I., Maciel, G. M., Bracht, A.,Ferreira, I., New phytochemicals as potential human anti-aging compounds: Reality, promise, and challenges. *Crit Rev Food Sci Nutr* **2018,** *58*, 942-957.
4. Liu, L., Guo, P., Wang, P., Zheng, S., Qu, Z.,Liu, N., The Review of Anti-aging Mechanism of Polyphenols on Caenorhabditis elegans. *Front Bioeng Biotechnol* **2021,** *9*, 635768.
5. Martel, J., Ojcius, D. M., Ko, Y. F., Chang, C. J.,Young, J. D., Antiaging effects of bioactive molecules isolated from plants and fungi. *Med. Res. Rev.* **2019,** *39*, 1515-1552.
6. Partridge, L., Fuentealba, M.,Kennedy, B. K., The quest to slow ageing through drug discovery. *Nature Reviews Drug Discovery* **2020,** *19*, 513-532.
7. Blagosklonny, M. V., An anti-aging drug today: from senescence-promoting genes to anti-aging pill. *Drug Discov. Today* **2007,** *12*, 218-224.
8. Yodhaanjali, J. R., Surya, R., Sumukha, K. C., Harini, G., Deshpande, G., Chandan, S.,Achar, R. R., Chapter 10 - Antiaging drugs, candidates, and food supplements: the journey so far. In *Anti-Aging Drug Discovery on the Basis of Hallmarks of Aging*, Singh, S. K.; Lin, C.-L.; Mishra, S. K., Eds. Academic Press: 2022; pp 191-239.
9. Ding, A. J., Zheng, S. Q., Huang, X. B., Xing, T. K., Wu, G. S., Sun, H. Y., Qi, S. H.,Luo, H. R., Current Perspective in the Discovery of Anti-aging Agents from Natural Products. *Nat Prod Bioprospect* **2017,** *7*, 335-404.
10. Sinclair, D. A.,Guarente, L., Small-Molecule Allosteric Activators of Sirtuins. *Annual Review of Pharmacology and Toxicology* **2014,** *54*, 363-380.
11. Wang, T.-y., Li, Q.,Bi, K.-s., Bioactive flavonoids in medicinal plants: Structure, activity and biological fate. *Asian Journal of Pharmaceutical Sciences* **2018,** *13*, 12-23.
12. Cătană, C.-S., Atanasov, A. G.,Berindan-Neagoe, I., Natural products with anti-aging potential: Affected targets and molecular mechanisms. *Biotechnol. Adv.* **2018,** *36*, 1649-1656.
13. Klimova, B., Novotny, M.,Kuca, K., Anti-Aging Drugs - Prospect of Longer Life? *Curr. Med. Chem.* **2018,** *25*, 1946-1953.
14. Arora, B. P., Anti-aging medicine. *Indian J. Plast. Surg.* **2008,** *41*, S130-133.
15. Pilcher, H. Anti-ageing pills are real, and some of us are taking them without knowing it. https://www.sciencefocus.com/the-human-body/anti-ageing-medication-health/ (accessed Apr 11, 2023).
16. Wesinka Top Ten Life Extension Drugs. https://www.esculape.com/bricabrac/toptenlifeextensiondrugs.html (accessed Apr 11, 2023).
17. The 14 Best Anti-Aging Vitamins and Supplements. https://www.healthline.com/nutrition/anti-aging-supplements (accessed Apr 11, 2023).
18. Thanapairoje, K., Junsiritrakhoon, S., Wichaiyo, S., Osman, M. A.,Supharattanasitthi, W., Anti-ageing effects of FDA-approved medicines: a focused review. *J. Basic Clin. Physiol. Pharmacol.* **2023**.

Anti-aging Strategies and Remedies: A Landscape of Research Progress and Promise

Rumiana Tenchov, Janet M. Sasso, Xinmei Wang, Qiongqiong Angela Zhou*

CAS, a division of the American Chemical Society

2540 Olentangy River Rd, Columbus, OH 43202, USA

Figure S1. Yearly NIH funding for projects related to anti-aging research.

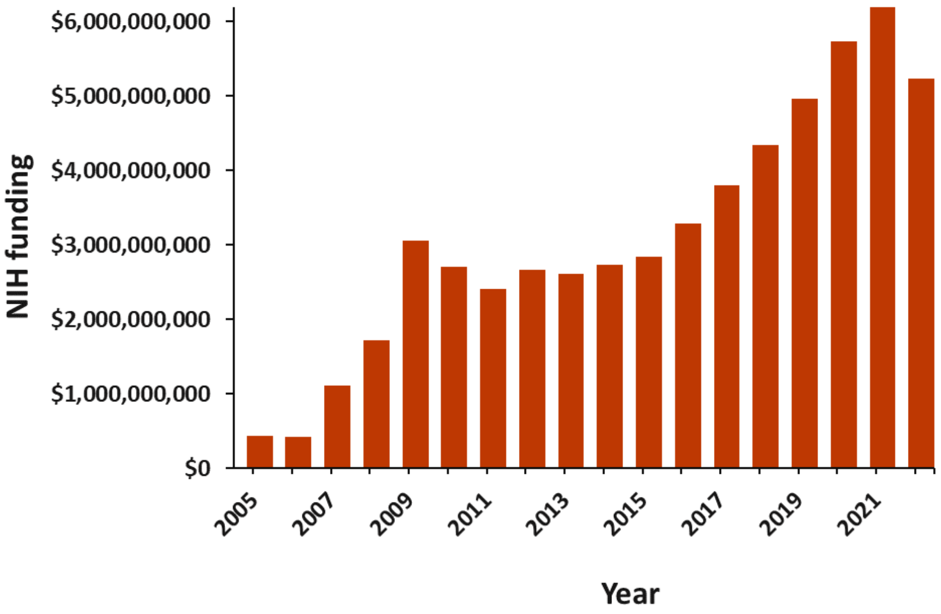

Figure S1. Yearly NIH funding for projects related to anti-aging research.
